# Associations between sleep traits and colorectal cancer: a mendelian randomization analysis

**DOI:** 10.3389/fonc.2025.1416243

**Published:** 2025-02-06

**Authors:** Xiangyue Meng, Enshuo Fan, Dan Lv, Yongjing Yang, Shixin Liu

**Affiliations:** ^1^ Department of Traditional Chinese Medicine, Changchun University of Chinese Medicine, Changchun, China; ^2^ Department of Radiotherapy, Jilin Cancer Hospital, Changchun, China

**Keywords:** colorectal cancer, chronotype, sleep duration, insomnia, daytime sleepiness, daytime napping, Mendelian randomization

## Abstract

**Background:**

Although many researches have shown a relationship between sleeping habits and the risk of developing colorectal cancer (CRC), there is a lack of data from randomized controlled trials (RCTs) to support this point. Hence, this study used Mendelian randomization (MR) to robustly assess whether five primary sleep characteristics are directly linked with the risk of CRC occurrence.

**Methods:**

In the performed study, the main Mendelian randomization analysis was conducted using approaches such as Inverse Variance Weighting (IVW), MR Egger, and weighted median method. To this end, five genetically independent variants associated with the sleep-related characteristics (chronotype, sleep duration, insomnia, daytime napping, and daytime fatigue) were identified and used as instrumental variables. Publicly accessible GWAS (Genome-Wide Association Study) data were used to identify these variants to investigate the putative causal relationships between sleep traits and CRC. Additionally, we conducted sensitivity analyses to minimize possible biases and verify the consistency of our results.

**Results:**

Mendelian randomization analyses showed that an morning chronotype reduces the risk of CRC with the IVW method, hence, odds ratio (OR) of 1.21 and 95% confidence interval (CI) of 0.67-0.93, which is statistically significant at P = 5.74E-03. Conversely, no significant evidence was found to suggest that sleep duration, insomnia, daytime napping, or daytime sleepiness have a direct causal impact on CRC risk according to the IVW analysis.

**Conclusions:**

Findings from our Mendelian randomization analyses suggest that an individual’s chronotype may contribute to an increased risk of CRC. It is advisable for individuals to adjust their sleep patterns as a preventative measure against CRC.

## Introduction

CRC occupies the third position in terms of prevalence among all cancers and is the second most common cause of death related to cancer (1). Data from 2023 show that CRC was diagnosed in approximately 153,020 individuals, resulting in 52,550 deaths. The evolution of surgical interventions and advancements in systemic treatments have enhanced the five-year survival rate for CRC, which has risen from 50% to 65% in various European regions (2). Despite an overarching decrease in CRC’s incidence and mortality rates, there’s an alarming uptrend in its occurrence among those under 50 years of age (3). Epidemiological studies have consistently identified several lifestyle and dietary elements as potential risk factors for CRC, such as extended periods of sitting, smoking, high alcohol consumption, and diets predominated by red or processed meats (4).

The investigation into the relationship between various lifestyle habits and the risk of cancer has intensified among researchers. Beyond the traditionally acknowledged risk factors such as physical activity levels, dietary habits, tobacco usage, alcohol consumption, and body weight, recent studies have identified sleep patterns—including the amount of sleep and the body’s natural sleep-wake cycle—as contributing factors to cancer risk, particularly with breast cancer highlighted in the literature (5, 6). Yet, the limited exploration of how sleep duration and insomnia related to colorectal cancer has remained unclear, with only a handful of observational studies addressing this connection (7–9). Moreover, research delving into the genetic underpinnings of the impact of sleep traits on colorectal cancer is exceedingly rare.

MR represents a statistical methodology that employs genetic variants as instrumental variables for probing the potential causal relationships between exposures and outcomes, utilizing data from observational studies (10). This approach emulates the conditions of a randomized clinical trial through the principle that genetic variants are randomly allocated at the moment of conception (11). This methodology boasts significant advantages. First, it utilizes genetic variation as a form of ‘natural experimentation’, where the random distribution of alleles at conception inherently disconnects these genetic factors from the environmental and lifestyle variables that frequently obscure the true relationships in observational research. Second, because the sequence and progression of a disease do not alter an individual’s germline genetic composition, MR effectively navigates around the pitfalls of reverse causation and confounding variables (12). Thus, MR is recognized as a potent and reliable strategy for elucidating causal links.

In an effort to expand upon previous research, this investigation employed Mendelian randomization, analyzing data from a comprehensive genetic study examining sleep patterns and GWAS concerning colorectal cancer. By exploring the potential causal relationships between sleep behaviors and the risk of colorectal cancer, this research aims to contribute towards the development of more precise prevention and treatment strategies for this disease.

## Materials and methods

### Data sources

Data pertaining to sleep traits and colorectal cancer have been compiled and made accessible online (See **Table 1**). This study did not necessitate ethical approval or informed consent, as it exclusively utilized data from previously published sources.

**Table 1:** Summary of genome-wide association studies (GWAS) datasets in our study.

Phenotype	Author, published year	Consortium	Sample size	PMID	Population
Chronotype	Jones SE et al, 2019 (14)	UKB	403,195	30696823	European
Sleep duration	Dashti HS et al, 2019 (15)	UKB	446,118	30846698	European
Insomnia	Jansen PR et al, 2019 (17)	UKB and 23andMe	1,331,010	30804565	European
Daytime sleepiness	Wang H et al, 2019 (18)	UKB	452,071	31409809	European
Daytime napping	Dashti HS et al,2021 (13)	UKB	452,663	33568662	European
Colorectal cancer	2023	FinnGen	314,193	NA	European

### Exposure

#### Daytime napping

A daytime nap refers to a brief period of sleep occurring during daylight hours. Summary data on the habit of napping were derived from a GWAS that included 452,633 adults of European descent registered in the UK Biobank (13). Participants were surveyed about their napping habits through a questionnaire that inquired, *“Do you engage in daytime napping?”* with the options for responses being *“yes”* or *“no”*.

#### Chronotype

The term chronotype refers to an individual’s inclination to either go to bed and wake up early or stay up late and rise later, with variations between these two extremes, which are also known as circadian preferences. Genetic associations related to chronotype were derived from published GWAS involving 403,195 individuals of European ancestry who were part of the UK Biobank (14). Participants provided information regarding their chronotype by answering the question “Do you consider yourself to be?” with several possible responses, including “Definitely a ‘morning’ person,” “More of a ‘morning’ than ‘evening’ person,” “More of an ‘evening’ than ‘morning’ person,” “Definitely an ‘evening’ person,” “Do not know,” or “Prefer not to answer”.

#### Sleep duration

The assessment of sleep duration within the UK Biobank involved 446,118 participants of European descent. This parameter was evaluated by querying participants on their total sleep hours over a 24-hour period, requiring responses in whole numbers. On average, participants reported 7.2 hours of sleep daily. For analytical purposes, sleep duration was treated as a continuous variable, which facilitated the categorization into two distinct groups: those with short sleep duration (less than 7 hours) and those experiencing long sleep duration (more than 9 hours). Furthermore, an interval defining normal sleep duration was established for those with 7 to less than 9 hours of sleep (15).

#### Insomnia

Insomnia, a prevalent sleep disturbance, manifests through challenges in initiating sleep or premature awakenings with subsequent inability to return to sleep, significantly deteriorating individuals’ quality of life (16). Analysis of GWAS summary statistics from a combined cohort of 1,331,010 participants, encompassing individuals from the UK Biobank and the 23andMe database, has elucidated a genetic predisposition to insomnia (17). The evaluation of insomnia-related symptoms necessitates that participants respond to the query, “Do you experience difficulty in initiating sleep at night, or find yourself waking up during the night?”

#### Daytime sleepiness

Insights into the link between daytime sleepiness and genetic factors were derived through analysis of GWAS data, which encompassed a cohort of 452,071 individuals of European ancestry registered with the UK Biobank (18). To evaluate daytime sleepiness, subjects provided responses to queries concerning unintentional sleep episodes, their alertness during the day, and the effort required to stave off sleep while engaged in work or educational activities.

### Outcome

The collection of genetic data related to CRC was facilitated through the FinnGen consortium, recognized as one of the preeminent genetic databases across Europe (https://www.finngen.fi/en). This comprehensive study, undertaken within the FinnGen project’s scope, involved the participation of 6,509 individuals diagnosed with CRC, juxtaposed against a substantial cohort of 287,137 controls. For researchers and interested parties, the dataset was made accessible through a specific link, designated in **Table 1**, enabling detailed examination and further analysis.

### Study design

In the context of MR studies, it is imperative to adhere to three critical presuppositions (19): Firstly, there should be a robust association between the genetic markers and the exposures (namely, sleep traits). Secondly, these genetic markers must not be influenced by any potential confounding variables. Thirdly, the relationship between the genetic markers and the outcome (in this case, colorectal cancer or CRC) should be mediated exclusively through the exposures (again, sleep traits). This methodology was also recently employed to explore the impact of sleep characteristics on the likelihood of developing various conditions, such as liver cancer and Systemic Lupus Erythematosus (20).

### Selection of genetic instruments

In our study to pinpoint optimal IVs for investigating the influence of sleep traits, we meticulously followed a structured protocol. Initially, we sifted through GWAS data to identify significant SNPs, adhering to stringent criteria (P-value less than 5 × 10^-8^ and r^2^ less than 0.1), ensuring only the most relevant genetic markers were considered. To control for the influence of weak IVs and potential distortion of results, the F-statistic formula, F = R^2^(n - k - 1)/k(1 - R^2^), was used, where ‘n’ is the total number of individuals in the exposure group, ‘k’ is the number of IVs used, and R^2^ is the proportion of variance in the exposure An F-value less than 10 was considered as a poor relationship between the IVs and the exposure which might result in the bias in the analysis. To maintain the independence of the chosen IVs, we checked LD among the SNPs, with the r^2^ threshold set to below 0.001 and the clumping distance of 1Mb (21), hence preventing common genetic signals. Finally, we meticulously chose SNPs for sleep traits, which coincided with allele frequencies at CRC outcomes, leaving out any palindromic SNPs to prevent uncertainty. When situations arose where direct SNPs linked with the exposure were absent in the outcome dataset, we performed substitute SNPs that had a high linkage disequilibrium (R^2^ greater than 0.8) with pertinent traits, improving the strength and perinatal of our instrumental variable selection.

### MR analysis

In the main MR analysis, we used the IVW approach. This approach involves a regression analysis in which the effect of SNPs on the outcome is plotted against their effect on the exposure, ignoring the intercept and using the inverse of the variance of the outcome as weights. Within the IVW framework, it is important to eliminate SNPs that display pleiotropic behavior. This exclusion is critical since the existence of horizontal pleiotropy violates one of the MR basic premises—no horizontal pleiotropic effects. These effects distort the causal inference derived from the MR analysis hence resulting in the identification of wrong causative links (22).

The MR-Egger approach is unique in the sense that it incorporates an intercept in the weighted linear regression analysis, using this intercept to measure the degree of horizontal pleiotropic effects (23). The IVW method, unlike the MR-Egger approach, is useful when genetic variants present with directional pleiotropy. It requires that pleiotropic effects remain uncorrelated with variant to exposure association and also assumes the absence of measurement error. However, it should be pointed out that these method is limited concerning the power of the other methods.

Method of weighted median (WM) requires cause to be linked to at least more than half variables considered effective. This approach, in combination with MR-Egger, helps to improve the estimates given by the IVW procedure.

### Sensitivity analysis

A comprehensive sensitivity analysis was performing in order to guarantee the validity and consistency of the results of our research. This method was developed to reveal any hidden biases, with special emphasis on gene pleiotropy and data consistency differences. Our analysis employed two sophisticated methods: and the MR-PRESSO technique and MR-Egger regression. The two approaches are equally good at managing issues that arise from horizontal pleiotropy. Particularly, MR-PRESSO method has a feature that allows the detection of outlier SNPs that can be removed from the analysis. That is why the method is suitable for the detailed evaluation of the influence of isolative SNPs on the results of the study. This stage also provides an opportunity for a critical comparison of the initial results and those which have been corrected for the presence of outlying values (22). Cochran’s Q test was used to further investigate the consistency of SNP estimates, and hence any estimate variances. Another important element of our methodology is the ‘leave-one-out’ sensitivity analysis which, systematically dropped all IVs to test its individual contribution to the aggregated MR estimates. Should the omission of any IV have a significant impact on the MR estimates as compared to the pooled data, the overall results are said to be sensitive to that IV and accordingly, the findings should be interpreted with caution.

We carefully identified secondary phenotypes connected to each SNP specified as an IV using the PhenoScanner to minimize the effect of possible horizontal pleiotropy from the confounding variables. This step entailed elimination of SNPs that could introduce bias in the association of sleep traits and CRC risk factors, subsequently improving the specificity of the analysis (24).

### Statistics analysis

In the present research, we applied the approach of Two-Sample Mendelian Randomization (TSMR) to examine the potential causative relationship between sleep characteristics and CRC. This analytical process was facilitated through the application of R software, version 4.4.0, making use of specialized packages: Two Sample MR, version 0.5.6 and MR-PRESSO, version 2.1 (25). The statistical significance threshold for our study was P value less than 0.05 which implied a relationship existed between the variables of interest and the health outcome that was being investigated.

## Result

**Figure 1:**
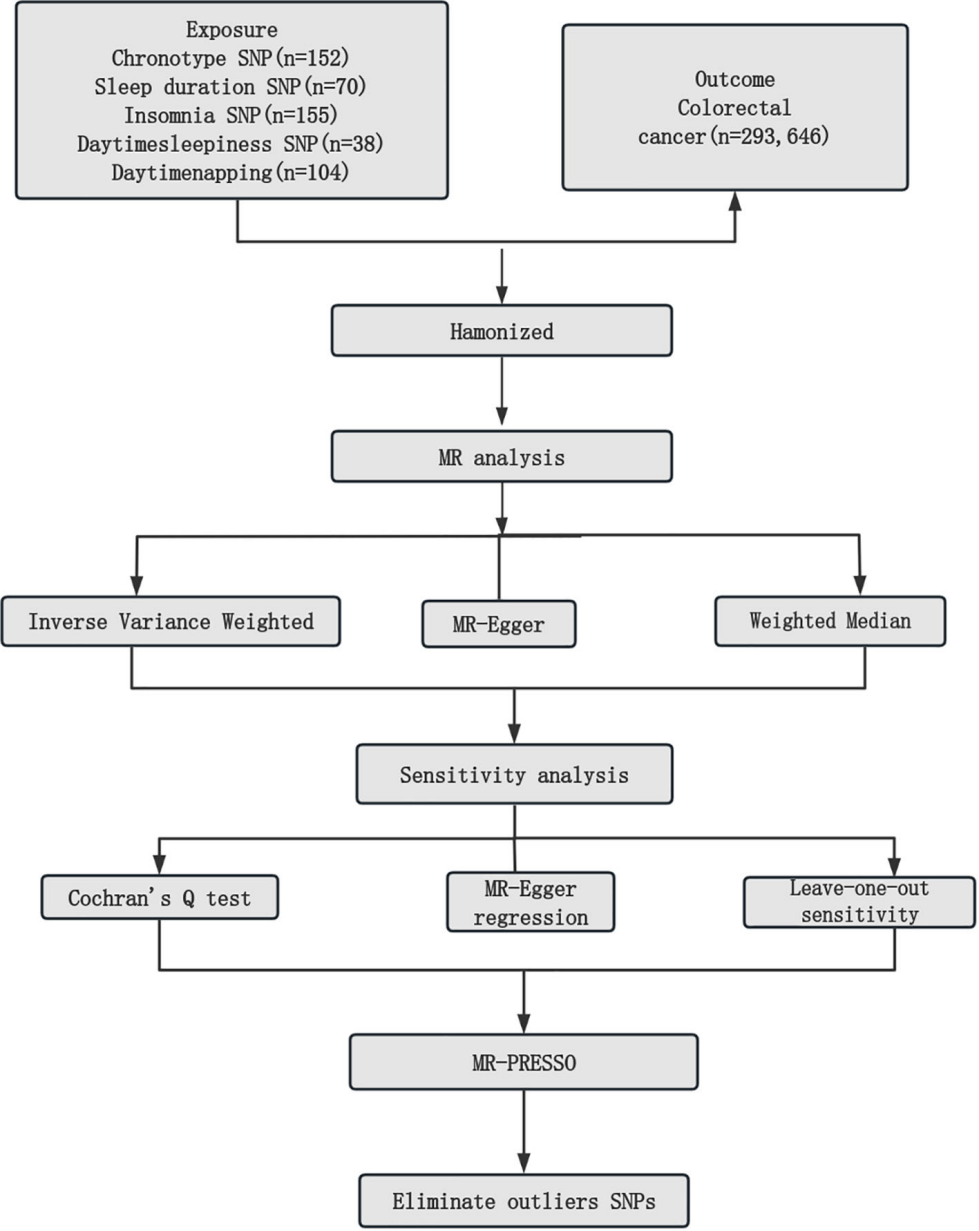
Mendelian randomization study workflow. MR, Mendelian randomization; SNPs, single nucleotide polymorphisms; MR-PRESSO, Mendelian Randomization Pleiotropy Residual Sum and Outlier.

**Figure 2:**
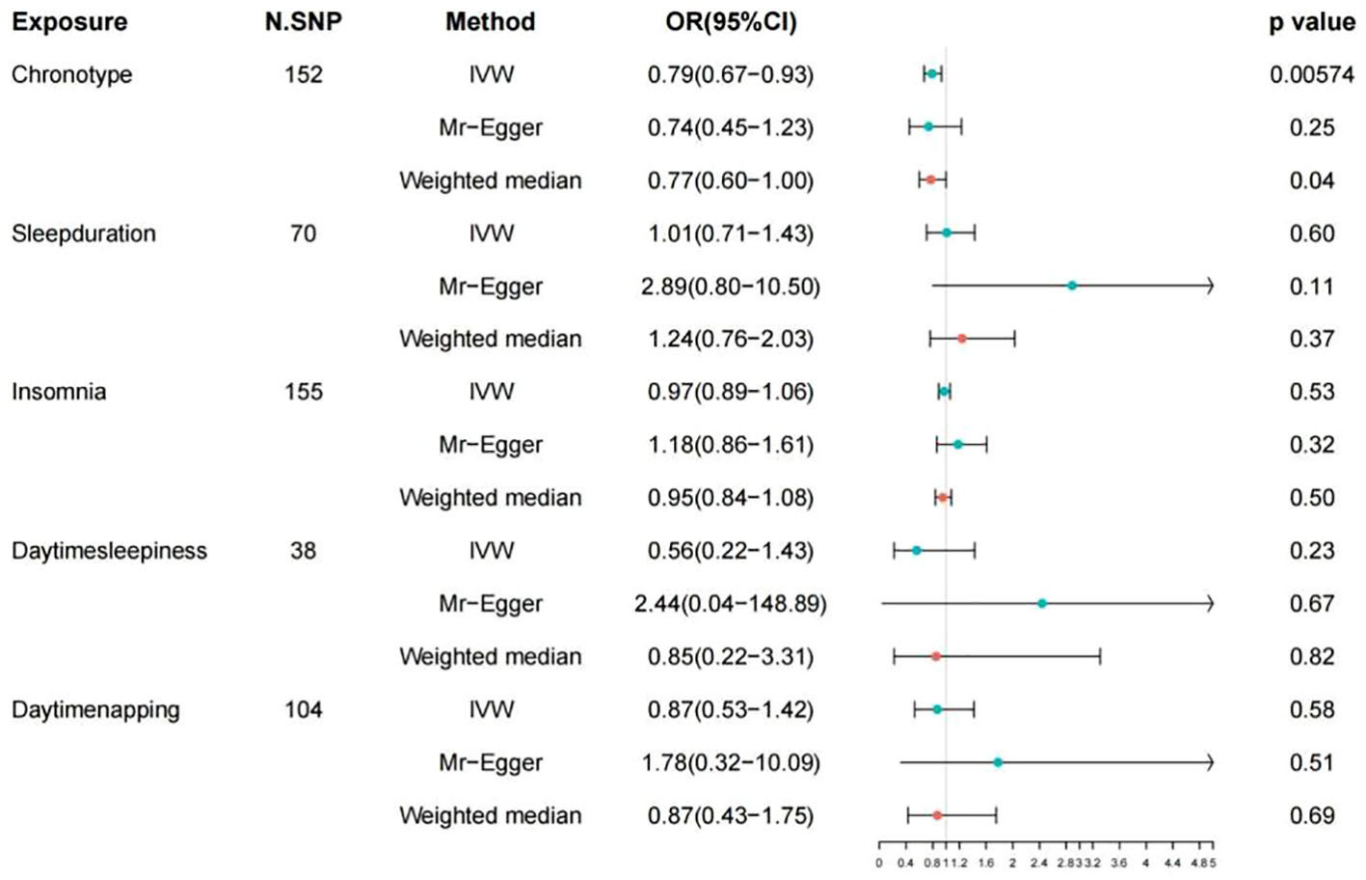
Associations of genetic liability to 5 sleep traits with risk of colorectal cancer in FinnGen study. OR, odd ratio; CI, confidence interval.

The detailed descriptions of the IVs are provided across **Supplementary Tables S1**-**S5**. **Supplementary Table S6** elaborates on the specifics of the instrument variables, including their Beta coefficients, standard errors (SE), and P values. The layout of the research methodology is depicted in **Figure 1**. The MR findings are comprehensively presented in **Table 2** and visualized in **Figure 2**.

### Chronotype and CRC

**Figure 3:**
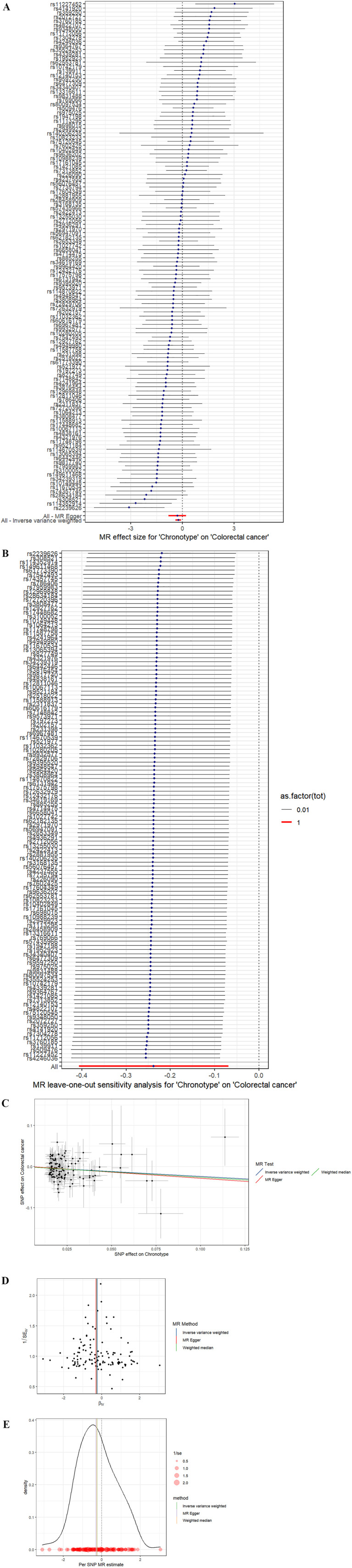
**(A)** Forest plot. The vertical axis represents the number assigned to each SNP, while the horizontal axis represents the confidence interval. **(B)** Leave-one-out sensitivity analysis. Circles indicate MR estimates for chronotype on CRC using IVW fixed effect method if each single nucleotide polymorphism was omitted in turn. The bars indicate the CI. **(C)** Scatter plot. The x-axis represents the effect of SNPs on exposure, and the y-axis represents the effect of SNPs on outcomes. The slope is less than 0, indicating that the exposure factor is a favorable factor for the outcome. **(D)** funnel plot showing the extent of heterogeneity among the individual Wald ratio estimates. **(E)** Density plot. The abscissa indicates the range of SNPs, the ordinate represents the probability at the corresponding SNP value point.

In the study spearheaded by Jones et al. (14),an initial pool of 15,155 SNPs was identified, each possessing genome-wide significance (P < 5 × 10^-8^) for potential use as IVs. The process of refining this list involved the removal of 15,002 SNPs due to their LD with other genetic variants, along with the exclusion of a duplicate SNP (rs73581564). Additionally, in the phase dedicated to correlating IVs with the outcomes, two SNPs were omitted owing to the lack of corresponding outcome data, and a further duplicate SNP (rs10520176) was eliminated. In the task of synchronizing the exposure and outcome datasets, 26 SNPs were disregarded for their palindromic nature. Consequently, a concise selection of 123 SNPs was finalized for inclusion in the MR analysis. The findings from the IVW analysis suggested a notable positive link between chronotype and the risk of CRC (Odds Ratio: 0.79, 95% CI: 0.67–0.93, P= 5.74E-03), as illustrated in **Table 2** and **Figure 3**. The MR-Egger regression analysis (intercept P=0.81) did not reveal any significant horizontal pleiotropic effects. Furthermore, the MR-PRESSO method identified no outlier SNPs (P = 0.711), as documented in **Table 1**, and the Cochrane Q test indicated an absence of heterogeneity among the SNPs (Q = 123.72, P = 0.41), also in **Table 1**. Employing the leave-one-out strategy underscored the resilience of the overall MR findings, even after the sequential exclusion of individual SNPs. **Supplementary Table S1** meticulously documents the IV details, affirming no presence of weak instrumental variable bias given that the F statistics for each SNP exceeded 10. The associations persisted after correction for multiple testing.

**Table 2:** MR analysis for the causality of sleep traits with the risk of CRC.

Exposure/Outcome	Nsnp	Methods	*OR* (95%*CI*)	*SE*	*P* value	Horizontal pleiotropy						Heterogeneity		
						MR-Egger regression			MR-PRESSO			Cochran’s Q	Q_df	P value
						Egger intercept	*SE*	P value	Global test P value	Global test RSSobs	Outliers			
Chronotype/CRC	152	IVW	0.79 (0.67-0.93)	0.09	5.74E-03	1.37E-03	5.67E-03	0.81	0.42	125.67	_	123.72	121	0.41
		MR Egger	0.74 (0.45-1.23)	0.26	0.25							123.67	120	0.39
		Weighted median	0.77 (0.60-1.00)	0.13	0.04									
Sleep duration/CRC	70	IVW	1.01 (0.71-1.43)	0.18	0.97	-0.02	0.01	0.10	0.20	68.34	_	66.27	57	0.19
		MR Egger	2.89 (0.80-10.50)	0.66	0.11							63.13	56	0.24
		Weighted median	1.24 (0.76-2.03)	0.24	0.37									
Insomnia/CRC	155	IVW	0.97 (0.89-1.06)	0.04	0.53	-8.71E-03	7.13E-03	0.22	0.03	164.44	rs769449 rs910187 rs1580173	162.10	129	0.03
		MR Egger	1.18 (0.86-1.61)	0.16	0.32							160.23	128	0.03
		Weighted median	0.95 (0.84-1.08)	0.06	0.45									
insomnia(after the removal of the outlier)/CRC	155	IVW	0.97 (0.89-1.06)	0.04	0.50	-7.31E-03	6.82E-03	0.29	0.13	146.96	_	144.79	127	0.13
		MR Egger	1.18 (0.86-1.61)	0.15	0.40							143.48	126	0.14
		Weighted median	0.95 (0.84-1.08)	0.06	0.45									
Daytime sleepiness/CRC	38	IVW	0.56 (0.22-1.43)	0.48	0.23	-0.01	0.02	0.48	0.72	28.47	_	26.89	32	0.72
		MR Egger	2.44 (0.04-148.89)	2.10	0.67							26.37	31	0.70
		Weighted median	0.85 (0.22-3.31)	0.69	0.82									
Daytime napping/CRC	104	IVW	0.87 (0.53-1.42)	0.25	0.58	-7.13E-03	8.44E-03	0.40	0.27	87.84	_	85.55	78	0.26
		MR Egger	1.78 (0.32-10.09)	0.88	0.51							84.77	77	0.26
		Weighted median	0.87 (0.43-1.75)	0.35	0.69									

*The result of recalculation after removing outliers.MR-PRESSO, MR-Pleiotropy Residual Sum and Outlier method. OR, odds ratio; CI, confidence interval; IVW, inverse-variance weighted. P-value corrected for False Discovery Rate.

### Sleep duration

In the study led by Dashti et al. (15), an initial collection of 7,924 SNPs, each achieving the threshold for genome-wide significance (P < 5 × 10^-8^), was gathered. The refinement process, which included a clumping strategy to reduce redundancy and the exclusion of duplicate SNPs, SNPs lacking associated outcome data, and palindromic SNPs, ultimately narrowed the pool to 58 SNPs for further investigation. The analysis conducted using the IVW method revealed no statistically significant causal relationship between sleep duration and the risk of CRC (Odds Ratio: 1.01, 95% CI: 0.71–1.43, P = 0.97), as detailed in **Table 2** and illustrated in **Supplementary Figures S1**. Additionally, both the MR-Egger intercept test (intercept P = 0.10) and the MR-PRESSO approach (P = 0.20) found no evidence of horizontal pleiotropy influencing the IV-outcome relationship, as reported in **Table 2**. The global Q statistic further confirmed the lack of significant heterogeneity among the SNPs (Q=66.27, P = 0.19), as mentioned in **Table 2**. With F-statistics for all SNPs exceeding 10, the analysis indicated a robust set of instrumental variables, free from the concern of weak instrumental variable bias.

### Insomnia

From an initial selection of 228 SNPs known to be linked with insomnia, a rigorous filtering process was applied. This process led to the removal of 73 SNPs due to LD and an additional 2 SNPs were excluded for lacking relevant outcome data. Further refinement was made by eliminating 22 palindromic SNPs during the harmonization of exposure and outcome data. Ultimately, 131 SNPs were deemed suitable for inclusion as IVs in the two-sample MR analysis. The F-statistics from this set of IVs indicated no significant risk of weak instrumental bias, as detailed in **Supplementary Table S3**. The IVW analysis revealed no statistically significant association between insomnia and the risk of CRC (Odds Ratio: 0.97, 95% CI: 0.89–1.06, P = 0.53), as shown in **Table 1** and **Supplementary Figure S2**. Despite this, the global Q statistic pointed to heterogeneity among the results (Q=160.23, P = 0.03). The MR-Egger method did not suggest the presence of horizontal pleiotropy (intercept P=0.22), while the MR-PRESSO method detected it (P = 0.03), identifying rs769449, rs910187, and rs1580173 as outliers. Removal of these outlier SNPs did not alter the findings (IVW, Odds Ratio: 0.97, 95% CI: 0.89–1.06, P = 0.50) from **Table 2**, but it did resolve the heterogeneity (P=0.14), underscoring the minimal evidence of weak instrument bias.

### Daytime sleepiness

In the course of examining the potential link between daytime sleepiness and colorectal cancer risk, our initial screening highlighted 5,657 SNPs significantly correlated (P < 5 × 10^-8^). A meticulous filtering procedure was then employed to exclude SNPs compromised by LD, resulting in the dismissal of 5,619 SNPs and an additional pair of duplicates. Furthermore, SNPs exhibiting palindromic characteristics were also excluded, leaving a total of 33 SNPs qualified for inclusion in the MR analysis. The subsequent analysis, utilizing IVW models, revealed no substantial link between daytime sleepiness and the likelihood of developing colorectal cancer (Odds Ratio: 0.56, 95% CI: 0.22–1.43, P = 0.23), as detailed in **Table 1** and depicted in **Supplementary Figure S3**. The MR-Egger intercept examination suggested no evidence of horizontal pleiotropy, with P-values exceeding 0.05. Additionally, the application of the MR-PRESSO test (P=0.72) identified no outliers, while the Cochran Q test demonstrated a lack of heterogeneity across the findings (Q=26.89, P=0.72). A comprehensive account of the instrumental variables is available in **Supplementary Table S4**. The F statistics for these genetic instruments exceeded 10, underscoring their robustness.

### Daytime napping

In the study conducted by Dashti et al. (13),an initial selection of 12,211 SNPs, each with a P-value less than 5 × 10^-8^, was identified for their significant association with daytime napping habits. Following this, seven SNPs found to be duplicates within the exposure dataset were excluded. Furthermore, a substantial number of variants, specifically 12,107 out of the 12,211, were removed due to LD with other SNPs in the set. Additionally, SNPs lacking corresponding outcome data and those identified as palindromic were also excluded from the analysis. This rigorous screening process resulted in the retention of 79 SNPs for subsequent two-sample MR analysis. The study found no causal relationship between daytime napping and colorectal cancer (CRC), as indicated by an odds ratio (OR) of 0.87 and a 95% confidence interval (CI) of (0.53–1.42), with a P-value of 0.58. Both the MR-Egger regression analysis, which yielded an intercept P-value of 0.40, and the MR-PRESSO method, with a P-value of 0.27, confirmed the lack of horizontal pleiotropy in the instrumental variables related to the examined outcomes. Moreover, the analysis showed no substantial heterogeneity in the link between daytime napping and CRC, with a Q value of 85.55 and a P-value of 0.26. The specifics of the instrumental variables utilized are provided in **Supplementary Table S5**. In addition, the F statistics for each single nucleotide polymorphism (SNP) effectively ruled out any weak instrumental variable bias, as all F statistics were above 10.

### Other analyses

In an effort to examine associations between 424 SNPs identified in the primary MR analysis and potential confounding factors, researchers turned to the PhenoScanner database. This detailed review pinpointed five potential confounders: BMI, frequency of alcohol consumption, history of smoking, and overall height, which are elaborated in **Supplementary Table S7**. By systematically excluding SNPs linked to these confounders with significant genome-wide associations, the integrity of the MR findings, along with the results from sensitivity analyses, remained robust and aligned with earlier reports. These outcomes, reinforcing the consistency of the analyses, are thoroughly documented in **Supplementary Table S8** and illustrated across **Supplementary Figures S5-S9**.

## Discussion

This investigation assessed the potential causal links between five sleep-related traits—chronotype, daytime napping, sleep duration, daytime sleepiness, and insomnia—and the risk of CRC. Findings indicated a significant positive correlation between chronotype and CRC incidence. Conversely, analyses did not demonstrate causal associations between CRC risk and other sleep traits such as daytime napping, the length of sleep, levels of daytime sleepiness, or the presence of insomnia.

Poor sleep quality is recognized as a potential contributor to cancer incidence and mortality. Previous research highlighted a correlation between the sleep deficit resulting from shift work and increased risks of type 2 diabetes, coronary heart disease, stroke, and cancer (26). A comprehensive population-based analysis revealed that night shift work elevates prostate cancer risk by disrupting circadian rhythms. Research from multiple regions has established an association between working night shifts and an elevated risk of breast cancer, particularly for tumors that test positive for estrogen receptor, progesterone receptor, and human epidermal growth factor receptor 2 (27, 28). Additionally, there’s an established connection between circadian rhythm disruptions and gastrointestinal cancers. These rhythms play crucial roles in regulating cell growth, immune equilibrium, gut barrier function, microbial equilibrium, and metabolic processes. Disorders in circadian rhythms lead to alterations in associated genes (CLOCK, PER, BMAL1) (29). Furthermore, sleep disturbances have been associated with cancer progression (30), with a study by Lin et al. revealing a significantly higher colorectal cancer prevalence among individuals with sleep disorders (31). As the most prevalent sleep disturbance, insomnia’s relationship with cancer risk was explored by Lin et al., showing that individuals with insomnia had a notably increased cancer risk, suggesting insomnia could serve as an early indicator of cancer development (32). Research also identified a relationship between insomnia and CRC incidents, where less frequent insomnia corresponded with a reduced CRC risk (33). Findings also indicated that longer sleep durations (8 hours and ≥9 hours) heighten colorectal cancer risk, with men facing a higher risk than women in cases of prolonged sleep (34). Additionally, extended napping has been linked to increased mortality among CRC survivors, with both napping frequency and duration correlating with elevated colorectal cancer risks (27, 35). Current data also point to sleep-disordered breathing and obstructive sleep apnea (OSA) as factors raising cancer incidence. A national cohort study established an association between obstructive sleep apnea and CRC (36). Chronotype, determined by biological circadian and sleep-wake rhythms, and influenced by work and social stress, may contribute to cancer development if evening chronotypes disrupt circadian rhythms due to misalignment with individual lifestyle behaviors (37).

The linkage between sleep disruptions and tumor development encompasses various molecular mechanisms. Primarily, sleep disturbances in individuals with cancer trigger an inflammatory response. These disruptions are recognized for initiating oxidative stress and systemic inflammation, which culminate in endothelial dysfunction and reduced oxygen supply to tissues. Such states provoke changes in sympathetic nervous system activity, immune responses, and the regulation of genes involved in cancer development (38). Additionally, melatonin, which governs sleep-wake cycles, plays a critical role in the carcinogenic impact associated with sleep disorders (39). This hormone has been shown to inhibit tumor expansion through multiple mechanisms. Specifically, melatonin promotes apoptosis in cancer cells, curtails their proliferation, and influences angiogenesis and metastasis. It also adjusts immune responses, impacts oncogenic signaling pathways, and offers antioxidative benefits (40). Disruptions in sleep patterns interfere with the normal release of melatonin.

Melatonin has been identified as a significant factor in preventing and slowing down the progression of CRC by inhibiting tumor cell proliferation and promoting cell death. Research has highlighted melatonin’s role in CRC prevention and treatment through its influence on lipid metabolism and the composition of the gut microbiome (41). According to Kvietkauskas et al., optimal levels of melatonin and glycine can diminish the growth of CRC liver metastases by exhibiting antiangiogenic properties (42). Therefore, melatonin emerges as a potential adjunctive therapy for advanced CRC. Moreover, the relationship between sleep disturbances and cancer progression is further explained through the lens of various hormones such as growth hormones, prolactin, dopamine, estrogen, leptin, and ghrelin. Ghrelin, in particular, is implicated in tumor advancement and reduced survival rates (43), while leptin is known to stimulate the production of pro-inflammatory cytokines TNF-α and IL-6, thereby facilitating cancer cell proliferation (44). Earlier research indicates that insufficient or suboptimal sleep can compromise immune functionality by inhibiting the release of hormones critical for immune stimulation, such as growth hormone, prolactin, and dopamine (45). The complex interplay between the HPA axis and the sympathetic nervous system is crucial for the maintenance of regular sleep patterns, which subsequently influence immune system responses. In our research, we could not confirm direct associations between daytime napping, feelings of sleepiness during the day, insomnia, sleep duration, and the incidence of colorectal cancer (CRC). Nonetheless, it is plausible that these sleep behaviors could have an indirect effect on the risk of developing CRC via the mechanisms mentioned above.

Our research offers several distinct advantages. At the forefront, it is the first of its kind to delve into the genetic relationships between a comprehensive spectrum of sleep patterns and CRC risk. This approach not only addresses the limitations present in prior observational and cross-sectional studies but also significantly enhances the breadth of research within this domain. To ensure the robustness of our findings, we meticulously selected genetic instruments from a vast pool of published genetic associations, utilizing large-scale GWAS to minimize the influence of potential weak instrument bias. Moreover, through detailed sensitivity and heterogeneity analyses, and a methodical evaluation of possible confounders, the study upholds the integrity and reliability of its conclusions. A pivotal aspect of our methodology was the use of the MR-PRESSO technique, which effectively identified and eliminated any potential bias in our Mendelian Randomization results due to the pleiotropic effects associated with sleep traits, further solidifying the validity of our findings.

While our research provides valuable insights, it also encounters certain limitations. Primarily, the dataset predominantly originates from European GWAS, involving participants from the UK Biobank who are generally more educated and healthier. This raises questions about the applicability of our findings across diverse populations. There might have been an overlap among participants, with daytime sleepiness potentially encompassing daytime napping, which, along with overlapping samples in the two-sample MR analysis, could have exaggerated the outcomes. Secondly, the reliance on self-reported questionnaires for gathering sleep-related data might have introduced biases. Future endeavors could benefit from incorporating device-measured sleep parameters. Thirdly, the absence of large-scale studies focused on specific age groups or genders precluded an in-depth analysis of these variables. Additionally, our application of a two-sample MR analysis, based on two extensive GWAS datasets, presupposes a linear association between sleep traits and colorectal cancer risk, not accounting for possible non-linear dynamics or stratified effects. Lastly, the study did not explore other sleep-related factors such as snoring, OSA, and overall sleep quality that may influence colorectal cancer risk. These aspects will be addressed as reliable data become available.

## Conclusion

In conclusion, our MR findings suggest that an individual’s chronotype has a contributory role in the development of CRC and propose that modifying sleep habits could serve as a preventive measure against CRC. While the study acknowledges the possibility of minor effects that it could not exclude, it underscores the need for more comprehensive MR analyses or extensive RCTs in the future to verify the influence of sleep traits on CRC risk. Concurrently, ongoing research aims to unravel the underlying biological mechanisms linking sleep-related characteristics with CRC.

## Data Availability

The original contributions presented in the study are included in the article/**Supplementary Material**. Further inquiries can be directed to the corresponding author.

## References

[1] Sung H Ferlay J Siegel RL Laversanne M Soerjomataram I Jemal A . Global cancer statistics 2020: GLOBOCAN estimates of incidence and mortality worldwide for 36 cancers in 185 countries. CA: Cancer J Clin. (2021) 71:209–49. doi: 10.3322/caac.21660 33538338

[2] Siegel RL Wagle NS Cercek A Smith RA Jemal A . Colorectal cancer statistics, 2023. CA: Cancer J Clin. (2023) 73:233–54. doi: 10.3322/caac.21772 36856579

[3] Sung H Siegel RL Rosenberg PS Jemal A . Emerging cancer trends among young adults in the USA: analysis of a population-based cancer registry. Lancet Public Health. (2019) 4:e137–47. doi: 10.1016/s2468-2667(18)30267-6 30733056

[4] Bray F Ferlay J Soerjomataram I Siegel RL Torre LA Jemal A . Global cancer statistics 2018: GLOBOCAN estimates of incidence and mortality worldwide for 36 cancers in 185 countries. CA: Cancer J Clin. (2018) 68:394–424. doi: 10.3322/caac.21492 30207593

[5] Huang BH Duncan MJ Cistulli PA Nassar N Hamer M Stamatakis E . Sleep and physical activity in relation to all-cause, cardiovascular disease and cancer mortality risk. Br J Sports Med. (2022) 56:718–24. doi: 10.1136/bjsports-2021-104046 34187783

[6] Richmond RC Anderson EL Dashti HS Jones SE Lane JM Strand LB . Investigating causal relations between sleep traits and risk of breast cancer in women: mendelian randomization study. BMJ (Clinical Res ed). (2019) 365:l2327. doi: 10.1136/bmj.l2327 PMC6592406 31243001

[7] Papantoniou K Castaño-Vinyals G Espinosa A Turner MC Martín-Sánchez V Casabonne D . Sleep duration and napping in relation to colorectal and gastric cancer in the MCC-Spain study. Sci Rep. (2021) 11:11822. doi: 10.1038/s41598-021-91275-3 34083698 PMC8175745

[8] Jiao L Duan Z Sangi-Haghpeykar H Hale L White DL El-Serag HB . Sleep duration and incidence of colorectal cancer in postmenopausal women. Br J Cancer. (2013) 108:213–21. doi: 10.1038/bjc.2012.561 PMC3553538 23287986

[9] Yoon K Shin CM Han K Jung JH Jin EH Lim JH . Risk of cancer in patients with insomnia: Nationwide retrospective cohort study (2009-2018). PloS One. (2023) 18:e0284494. doi: 10.1371/journal.pone.0284494 37083623 PMC10121030

[10] Sekula P Del Greco MF Pattaro C Köttgen A . Mendelian randomization as an approach to assess causality using observational data. J Am Soc Nephrol: JASN. (2016) 27:3253–65. doi: 10.1681/asn.2016010098 PMC5084898 27486138

[11] Sanderson E Glymour MM Holmes MV Kang H Morrison J Munafò MR . Mendelian randomization. Nat Rev Methods Primers. (2022) 2. doi: 10.1038/s43586-021-00092-5 PMC7614635 37325194

[12] Skrivankova VW Richmond RC Woolf BAR Yarmolinsky J Davies NM Swanson SA . Strengthening the reporting of observational studies in epidemiology using mendelian randomization: the STROBE-MR statement. Jama. (2021) 326:1614–21. doi: 10.1001/jama.2021.18236 34698778

[13] Dashti HS Daghlas I Lane JM Huang Y Udler MS Wang H . Genetic determinants of daytime napping and effects on cardiometabolic health. Nat Commun. (2021) 12:900. doi: 10.1038/s41467-020-20585-3 33568662 PMC7876146

[14] Jones SE Lane JM Wood AR van Hees VT Tyrrell J Beaumont RN . Genome-wide association analyses of chronotype in 697,828 individuals provides insights into circadian rhythms. Nat Commun. (2019) 10:343. doi: 10.1038/s41467-018-08259-7 30696823 PMC6351539

[15] Dashti HS Jones SE Wood AR Lane JM van Hees VT Wang H . Genome-wide association study identifies genetic loci for self-reported habitual sleep duration supported by accelerometer-derived estimates. Nat Commun. (2019) 10:1100. doi: 10.1038/s41467-019-08917-4 30846698 PMC6405943

[16] Perlis ML Posner D Riemann D Bastien CH Teel J Thase M . Insomnia. Lancet (London England). (2022) 400:1047–60. doi: 10.1016/s0140-6736(22)00879-0 36115372

[17] Jansen PR Watanabe K Stringer S Skene N Bryois J Hammerschlag AR . Genome-wide analysis of insomnia in 1,331,010 individuals identifies new risk loci and functional pathways. Nat Genet. (2019) 51:394–403. doi: 10.1038/s41588-018-0333-3 30804565

[18] Wang H Lane JM Jones SE Dashti HS Ollila HM Wood AR . Genome-wide association analysis of self-reported daytime sleepiness identifies 42 loci that suggest biological subtypes. Nat Commun. (2019) 10:3503. doi: 10.1038/s41467-019-11456-7 31409809 PMC6692391

[19] Lawlor DA . Commentary: Two-sample Mendelian randomization: opportunities and challenges. Int J Epidemiol. (2016) 45:908–15. doi: 10.1093/ije/dyw127 PMC5005949 27427429

[20] Sang N Gao RC Zhang MY Wu ZZ Wu ZG Wu GC . Causal relationship between sleep traits and risk of systemic lupus erythematosus: A two-sample mendelian randomization study. Front Immunol. (2022) 13:918749. doi: 10.3389/fimmu.2022.918749 35784289 PMC9248809

[21] Chang CC Chow CC Tellier LC Vattikuti S Purcell SM Lee JJ . Second-generation PLINK: rising to the challenge of larger and richer datasets. GigaScience. (2015) 4:7. doi: 10.1186/s13742-015-0047-8 25722852 PMC4342193

[22] Verbanck M Chen CY Neale B Do R . Detection of widespread horizontal pleiotropy in causal relationships inferred from Mendelian randomization between complex traits and diseases. Nat Genet. (2018) 50:693–8. doi: 10.1038/s41588-018-0099-7 PMC6083837 29686387

[23] Bowden J Davey Smith G Burgess S . Mendelian randomization with invalid instruments: effect estimation and bias detection through Egger regression. Int J Epidemiol. (2015) 44:512–25. doi: 10.1093/ije/dyv080 PMC4469799 26050253

[24] Staley JR Blackshaw J Kamat MA Ellis S Surendran P Sun BB . PhenoScanner: a database of human genotype-phenotype associations. Bioinf (Oxford England). (2016) 32:3207–9. doi: 10.1093/bioinformatics/btw373 PMC5048068 27318201

[25] Hemani G Zheng J Elsworth B Wade KH Haberland V Baird D . The MR-Base platform supports systematic causal inference across the human phenome. eLife. (2018) 7. doi: 10.7554/eLife.34408 PMC5976434 29846171

[26] Kecklund G Axelsson J . Health consequences of shift work and insufficient sleep. BMJ (Clinical Res ed). (2016) 355:i5210. doi: 10.1136/bmj.i5210 27803010

[27] Papantoniou K Castaño-Vinyals G Espinosa A Aragonés N Pérez-Gómez B Burgos J . Night shift work, chronotype and prostate cancer risk in the MCC-Spain case-control study. Int J Cancer. (2015) 137:1147–57. doi: 10.1002/ijc.29400 25530021

[28] Cordina-Duverger E Menegaux F Popa A Rabstein S Harth V Pesch B . Night shift work and breast cancer: a pooled analysis of population-based case-control studies with complete work history. Eur J Epidemiol. (2018) 33:369–79. doi: 10.1007/s10654-018-0368-x 29464445

[29] Mogavero MP DelRosso LM Fanfulla F Bruni O Ferri R . Sleep disorders and cancer: State of the art and future perspectives. Sleep Med Rev. (2021) 56:101409. doi: 10.1016/j.smrv.2020.101409 33333427

[30] Wu Zheng SM Chen JW Huang YM Chen WM Wu SY . Effect of sleep disorders on the risks of cancers and site-specific cancers. Sleep Med. (2022) 100:254–61. doi: 10.1016/j.sleep.2022.08.014 36122507

[31] Lin CL Liu TC Wang YN Chung CH Chien WC . The association between sleep disorders and the risk of colorectal cancer in patients: A population-based nested case-control study. In Vivo (Athens Greece). (2019) 33:573–9. doi: 10.21873/invivo.11513 PMC6506283 30804144

[32] Senaratna CV Perret JL Lodge CJ Lowe AJ Campbell BE Matheson MC . Prevalence of obstructive sleep apnea in the general population: A systematic review. Sleep Med Rev. (2017) 34:70–81. doi: 10.1016/j.smrv.2016.07.002 27568340

[33] Chen J Chen N Huang T Huang N Zhuang Z Liang H . Sleep pattern, healthy lifestyle and colorectal cancer incidence. Sci Rep. (2022) 12:18317. doi: 10.1038/s41598-022-21879-w 36316431 PMC9622719

[34] Zhang X Giovannucci EL Wu K Gao X Hu F Ogino S . Associations of self-reported sleep duration and snoring with colorectal cancer risk in men and women. Sleep. (2013) 36:681–8. doi: 10.5665/sleep.2626 PMC3624822 23633750

[35] Xiao Q Arem H Pfeiffer R Matthews C . Prediagnosis sleep duration, napping, and mortality among colorectal cancer survivors in a large US cohort. Sleep. (2017) 40. doi: 10.1093/sleep/zsx010 PMC5806565 28329353

[36] Chen CY Hu JM Shen CJ Chou YC Tian YF Chen YC . Increased incidence of colorectal cancer with obstructive sleep apnea: a nationwide population-based cohort study. Sleep Med. (2020) 66:15–20. doi: 10.1016/j.sleep.2019.02.016 31785565

[37] Erren TC Morfeld P Foster RG Reiter RJ Groß JV Westermann IK . Sleep and cancer: Synthesis of experimental data and meta-analyses of cancer incidence among some 1,500,000 study individuals in 13 countries. Chronobiol Int. (2016) 33:325–50. doi: 10.3109/07420528.2016.1149486 27003385

[38] Martínez C Juarranz Y Gutiérrez-Cañas I Carrión M Pérez-García S Villanueva-Romero R . A clinical approach for the use of VIP axis in inflammatory and autoimmune diseases. Int J Mol Sci. (2019) 21. doi: 10.3390/ijms21010065 PMC6982157 31861827

[39] Viswanathan AN Schernhammer ES . Circulating melatonin and the risk of breast and endometrial cancer in women. Cancer Lett. (2009) 281:1–7. doi: 10.1016/j.canlet.2008.11.002 19070424 PMC2735793

[40] Park SY Jang WJ Yi EY Jang JY Jung Y Jeong JW . Melatonin suppresses tumor angiogenesis by inhibiting HIF-1alpha stabilization under hypoxia. J Pineal Res. (2010) 48:178–84. doi: 10.1111/j.1600-079x.2009.00742.x 20449875

[41] Pan S Guo Y Hong F Xu P Zhai Y . Therapeutic potential of melatonin in colorectal cancer: Focus on lipid metabolism and gut microbiota. *Biochimica et biophysica acta* . Mol Basis Dis. (2022) 1868:166281. doi: 10.1016/j.bbadis.2021.166281 34610472

[42] Kvietkauskas M Zitkute V Leber B Strupas K Stiegler P Schemmer P . Dietary melatonin and glycine decrease tumor growth through antiangiogenic activity in experimental colorectal liver metastasis. Nutrients. (2021) 13. doi: 10.3390/nu13062035 PMC8231877 34199311

[43] Chopin L Walpole C Seim I Cunningham P Murray R Whiteside E . Ghrelin and cancer. Mol Cell Endocrinol. (2011) 340:65–9. doi: 10.1016/j.mce.2011.04.013 21616120

[44] Walker WH 2nd Borniger JC . Molecular mechanisms of cancer-Induced sleep disruption. Int J Mol Sci. (2019) 20. doi: 10.3390/ijms20112780 PMC6600154 31174326

[45] Lange T Dimitrov S Born J . Effects of sleep and circadian rhythm on the human immune system. Ann New York Acad Sci. (2010) 1193:48–59. doi: 10.1111/j.1749-6632.2009.05300.x 20398008

