# Supplementary material for: Associations between sleep traits and colorectal cancer: a mendelian randomization analysis
Source: Front Oncol. 2025 Feb 6;15:1416243. doi: 10.3389/fonc.2025.1416243 (PMC11839420; doi:10.3389/fonc.2025.1416243)
Supplement: Supplementary file 1 [file DataSheet1.docx]

**Supplementary materials**

**Supplementary Table 1.** The summary information for instrumental variables of chronotype.

| **Sort** | **Phenotype** | **SNP** | **effect_allele** | **other_allele** | **beta** | **se** | **pval** |
| --- | --- | --- | --- | --- | --- | --- | --- |
| 1 | Chronotype | rs61773390 | G | T | -0.0340792 | 0.00337553 | 1.20E-23 |
| 2 | Chronotype | rs17448682 | C | T | -0.0221528 | 0.00318247 | 2.20E-12 |
| 3 | Chronotype | rs3816454 | T | G | 0.020082 | 0.00278156 | 4.90E-13 |
| 4 | Chronotype | rs12140153 | G | T | 0.0340109 | 0.00468803 | 1.60E-13 |
| 5 | Chronotype | rs7547493 | A | G | -0.0376299 | 0.00350319 | 3.90E-27 |
| 6 | Chronotype | rs6658041 | G | A | -0.0152482 | 0.00274124 | 1.70E-08 |
| 7 | Chronotype | rs11588913 | G | A | 0.0154525 | 0.00273856 | 1.50E-08 |
| 8 | Chronotype | rs72720396 | A | G | -0.0271261 | 0.00318521 | 8.40E-18 |
| 9 | Chronotype | rs4949980 | A | G | -0.0321893 | 0.00539402 | 2.70E-09 |
| 10 | Chronotype | rs17575798 | G | A | 0.0225553 | 0.00339377 | 4.20E-11 |
| 11 | Chronotype | rs11587758 | G | A | -0.0241103 | 0.0027375 | 5.50E-19 |
| 12 | Chronotype | rs140206235 | C | T | -0.0553854 | 0.00996801 | 2.30E-08 |
| 13 | Chronotype | rs975025 | C | T | 0.0321062 | 0.00502525 | 1.30E-10 |
| 14 | Chronotype | rs509476 | T | C | 0.1137 | 0.0078575 | 5.70E-47 |
| 15 | Chronotype | rs13011556 | C | G | -0.0227424 | 0.00316075 | 1.10E-12 |
| 16 | Chronotype | rs2712056 | C | T | -0.022175 | 0.00345992 | 5.10E-11 |
| 17 | Chronotype | rs75120545 | C | T | -0.0613917 | 0.00828587 | 1.10E-13 |
| 18 | Chronotype | rs786406 | A | G | -0.0235784 | 0.00293536 | 1.90E-16 |
| 19 | Chronotype | rs10495976 | A | T | -0.0178512 | 0.00276698 | 5.10E-11 |
| 20 | Chronotype | rs7602425 | C | T | -0.031168 | 0.00509212 | 8.50E-10 |
| 21 | Chronotype | rs359250 | G | T | -0.0168642 | 0.0027885 | 9.20E-10 |
| 22 | Chronotype | rs812925 | C | G | -0.0205641 | 0.00280786 | 6.90E-13 |
| 23 | Chronotype | rs2311837 | T | C | -0.0151285 | 0.00272441 | 3.60E-08 |
| 24 | Chronotype | rs2422413 | T | C | -0.0162894 | 0.0027689 | 3.60E-09 |
| 25 | Chronotype | rs10520176 | T | C | 0.0231498 | 0.00268932 | 1.40E-17 |
| 26 | Chronotype | rs28380327 | A | T | 0.0205097 | 0.00277698 | 2.90E-13 |
| 27 | Chronotype | rs1947198 | C | T | -0.0241964 | 0.00409707 | 1.80E-09 |
| 28 | Chronotype | rs197273 | A | G | 0.0158713 | 0.00269376 | 1.30E-08 |
| 29 | Chronotype | rs1064213 | G | A | -0.019141 | 0.00268602 | 1.30E-12 |
| 30 | Chronotype | rs114670539 | C | T | -0.0342784 | 0.00580825 | 4.60E-09 |
| 31 | Chronotype | rs57435966 | C | T | 0.0549152 | 0.0047647 | 2.60E-30 |
| 32 | Chronotype | rs114870822 | G | A | -0.0697522 | 0.0120259 | 2.00E-09 |
| 33 | Chronotype | rs62182135 | C | A | 0.0179439 | 0.00284795 | 3.70E-10 |
| 34 | Chronotype | rs149611468 | T | C | 0.0776374 | 0.0125983 | 8.50E-10 |
| 35 | Chronotype | rs6442446 | A | G | 0.0174738 | 0.00297308 | 3.10E-09 |
| 36 | Chronotype | rs9817740 | A | G | 0.0179438 | 0.00326142 | 2.00E-08 |
| 37 | Chronotype | rs13059636 | A | G | -0.0182698 | 0.00270236 | 1.60E-11 |
| 38 | Chronotype | rs114848860 | A | T | -0.0555837 | 0.00872612 | 3.60E-10 |
| 39 | Chronotype | rs11712056 | T | C | 0.0200896 | 0.00270384 | 1.60E-13 |
| 40 | Chronotype | rs13316611 | G | T | -0.0178305 | 0.0030789 | 1.70E-08 |
| 41 | Chronotype | rs11924337 | G | C | -0.0190701 | 0.00301826 | 2.20E-10 |
| 42 | Chronotype | rs957501 | T | A | 0.0154211 | 0.00284661 | 3.00E-08 |
| 43 | Chronotype | rs1800828 | C | G | 0.0176235 | 0.00308462 | 1.70E-08 |
| 44 | Chronotype | rs13065394 | G | T | 0.017504 | 0.00296404 | 3.00E-09 |
| 45 | Chronotype | rs885255 | T | C | 0.0169773 | 0.00283418 | 4.40E-09 |
| 46 | Chronotype | rs9831488 | A | G | -0.0183592 | 0.00284523 | 8.40E-11 |
| 47 | Chronotype | rs3850174 | T | A | 0.0194471 | 0.00309938 | 6.80E-10 |
| 48 | Chronotype | rs2239626 | T | C | -0.0202758 | 0.00292696 | 2.30E-12 |
| 49 | Chronotype | rs231398 | G | A | 0.0207867 | 0.00367183 | 8.40E-09 |
| 50 | Chronotype | rs28634184 | C | T | 0.0176363 | 0.00310689 | 2.80E-08 |
| 51 | Chronotype | rs7691121 | C | G | 0.0220069 | 0.00317772 | 5.60E-12 |
| 52 | Chronotype | rs4339281 | A | G | 0.0223188 | 0.00404047 | 3.20E-08 |
| 53 | Chronotype | rs4241964 | T | G | -0.020735 | 0.00270449 | 1.10E-14 |
| 54 | Chronotype | rs10067113 | C | T | -0.016251 | 0.00277635 | 6.30E-09 |
| 55 | Chronotype | rs7701529 | A | T | -0.0188448 | 0.00317247 | 3.60E-09 |
| 56 | Chronotype | rs11748798 | G | T | 0.0203788 | 0.00309922 | 5.20E-11 |
| 57 | Chronotype | rs56947091 | C | T | -0.0191881 | 0.00268737 | 6.00E-13 |
| 58 | Chronotype | rs12055234 | G | A | -0.0188521 | 0.00286239 | 4.00E-11 |
| 59 | Chronotype | rs286805 | A | T | 0.0143071 | 0.00269548 | 4.50E-08 |
| 60 | Chronotype | rs1027742 | A | G | 0.0183119 | 0.00306482 | 2.80E-09 |
| 61 | Chronotype | rs4246036 | C | T | 0.0237433 | 0.00295896 | 2.40E-15 |
| 62 | Chronotype | rs7735794 | G | A | -0.0201147 | 0.00340433 | 4.70E-09 |
| 63 | Chronotype | rs9395520 | C | T | -0.0231272 | 0.00291912 | 2.10E-15 |
| 64 | Chronotype | rs521977 | T | G | 0.0159787 | 0.00286438 | 2.10E-08 |
| 65 | Chronotype | rs4714475 | G | T | 0.0190271 | 0.00317856 | 1.60E-09 |
| 66 | Chronotype | rs62405438 | T | A | -0.0201979 | 0.00350204 | 6.30E-09 |
| 67 | Chronotype | rs2653349 | A | G | 0.0387249 | 0.00327448 | 5.20E-32 |
| 68 | Chronotype | rs2881955 | C | T | -0.0161216 | 0.00300229 | 4.30E-08 |
| 69 | Chronotype | rs60616179 | A | G | 0.0375809 | 0.00594669 | 1.10E-10 |
| 70 | Chronotype | rs827749 | T | C | -0.0236615 | 0.00286775 | 1.00E-16 |
| 71 | Chronotype | rs9364767 | T | G | 0.0158538 | 0.00269933 | 5.40E-09 |
| 72 | Chronotype | rs9348050 | T | C | 0.016638 | 0.00269048 | 7.50E-10 |
| 73 | Chronotype | rs10280205 | T | C | 0.0174497 | 0.00291239 | 1.40E-09 |
| 74 | Chronotype | rs17161045 | T | C | 0.0206395 | 0.0028029 | 7.20E-14 |
| 75 | Chronotype | rs6967481 | C | T | -0.0206433 | 0.00269925 | 6.90E-15 |
| 76 | Chronotype | rs2968511 | C | G | -0.0174599 | 0.00291928 | 4.90E-09 |
| 77 | Chronotype | rs202157 | C | T | 0.0255486 | 0.0029433 | 4.10E-18 |
| 78 | Chronotype | rs4729854 | T | A | 0.0287186 | 0.00275036 | 3.30E-25 |
| 79 | Chronotype | rs1113295 | T | G | 0.0156591 | 0.00270408 | 6.80E-09 |
| 80 | Chronotype | rs2971970 | T | G | 0.0188272 | 0.00325601 | 6.00E-09 |
| 81 | Chronotype | rs35524253 | G | A | -0.015862 | 0.00281259 | 5.80E-09 |
| 82 | Chronotype | rs11786306 | G | C | -0.0183309 | 0.00283083 | 8.70E-11 |
| 83 | Chronotype | rs13255030 | A | G | 0.0162198 | 0.00273214 | 2.80E-09 |
| 84 | Chronotype | rs12541362 | A | T | -0.0213568 | 0.00282446 | 3.50E-14 |
| 85 | Chronotype | rs769066 | T | C | -0.0219436 | 0.00348289 | 3.20E-10 |
| 86 | Chronotype | rs4321976 | T | C | 0.0232959 | 0.00324034 | 3.10E-13 |
| 87 | Chronotype | rs3100052 | A | G | 0.0170795 | 0.00276058 | 6.90E-10 |
| 88 | Chronotype | rs3808477 | C | T | -0.0215613 | 0.00299116 | 2.80E-13 |
| 89 | Chronotype | rs34340407 | T | C | -0.0184548 | 0.0031152 | 4.30E-09 |
| 90 | Chronotype | rs6477309 | C | T | -0.0174399 | 0.00286276 | 8.00E-10 |
| 91 | Chronotype | rs308521 | T | C | 0.0199718 | 0.00275427 | 3.70E-13 |
| 92 | Chronotype | rs62553781 | C | T | 0.0504004 | 0.00734656 | 1.20E-11 |
| 93 | Chronotype | rs4838161 | T | G | 0.0161104 | 0.00270516 | 2.20E-09 |
| 94 | Chronotype | rs10988239 | C | T | 0.0186334 | 0.00273304 | 1.60E-11 |
| 95 | Chronotype | rs34619169 | G | A | -0.0169119 | 0.00291571 | 3.80E-09 |
| 96 | Chronotype | rs28458909 | C | T | 0.0432803 | 0.00407749 | 2.60E-26 |
| 97 | Chronotype | rs4948547 | C | A | 0.0190042 | 0.00307909 | 1.20E-09 |
| 98 | Chronotype | rs10823233 | T | C | -0.0151686 | 0.00270212 | 4.30E-08 |
| 99 | Chronotype | rs3808964 | G | T | -0.0157096 | 0.00279601 | 2.70E-08 |
| 100 | Chronotype | rs72632979 | A | G | 0.021581 | 0.00357606 | 1.20E-09 |
| 101 | Chronotype | rs10742179 | A | G | 0.0182967 | 0.00306465 | 1.50E-09 |
| 102 | Chronotype | rs4141920 | G | A | 0.0155932 | 0.00270207 | 4.60E-09 |
| 103 | Chronotype | rs11032362 | G | A | -0.0395538 | 0.00467579 | 4.80E-17 |
| 104 | Chronotype | rs34239319 | G | T | -0.0280114 | 0.00443834 | 3.90E-10 |
| 105 | Chronotype | rs3168135 | G | A | 0.0207725 | 0.0031468 | 2.90E-11 |
| 106 | Chronotype | rs11227452 | G | A | -0.0194632 | 0.00358768 | 4.60E-08 |
| 107 | Chronotype | rs4237555 | C | T | -0.0164771 | 0.00269332 | 6.00E-10 |
| 108 | Chronotype | rs4936291 | A | G | -0.0179295 | 0.00283312 | 3.80E-10 |
| 109 | Chronotype | rs74357745 | A | G | 0.026546 | 0.00412156 | 8.70E-11 |
| 110 | Chronotype | rs7977610 | A | T | 0.0160414 | 0.0028718 | 2.10E-08 |
| 111 | Chronotype | rs12811046 | A | G | 0.0169679 | 0.00270358 | 6.20E-10 |
| 112 | Chronotype | rs7313852 | G | A | 0.029649 | 0.00271587 | 2.70E-27 |
| 113 | Chronotype | rs77384811 | G | C | 0.0247483 | 0.00372778 | 2.80E-11 |
| 114 | Chronotype | rs7959983 | T | C | -0.0195258 | 0.00272849 | 9.50E-13 |
| 115 | Chronotype | rs7304278 | A | G | -0.020255 | 0.00302264 | 2.20E-11 |
| 116 | Chronotype | rs80097534 | G | T | 0.0281025 | 0.00455453 | 9.30E-10 |
| 117 | Chronotype | rs9597250 | C | A | 0.0213068 | 0.00343801 | 6.90E-10 |
| 118 | Chronotype | rs9573971 | A | G | 0.0727759 | 0.00743443 | 3.60E-22 |
| 119 | Chronotype | rs17267683 | C | G | 0.0197414 | 0.00316651 | 2.70E-10 |
| 120 | Chronotype | rs9521184 | T | C | 0.0148855 | 0.00270389 | 3.00E-08 |
| 121 | Chronotype | rs1952923 | G | A | 0.016877 | 0.00305963 | 3.60E-08 |
| 122 | Chronotype | rs698015 | C | T | -0.0161876 | 0.0028566 | 8.80E-09 |
| 123 | Chronotype | rs7148842 | C | T | 0.0153777 | 0.00278312 | 1.80E-08 |
| 124 | Chronotype | rs10149448 | A | G | 0.0164465 | 0.00275282 | 2.70E-09 |
| 125 | Chronotype | rs12432176 | C | A | -0.0161131 | 0.00278357 | 6.50E-09 |
| 126 | Chronotype | rs59986227 | C | G | -0.0178401 | 0.00310217 | 8.30E-09 |
| 127 | Chronotype | rs9932577 | C | A | 0.0166195 | 0.00271482 | 6.80E-10 |
| 128 | Chronotype | rs12927162 | A | G | 0.0286498 | 0.00300067 | 6.80E-22 |
| 129 | Chronotype | rs1421085 | T | C | -0.0275621 | 0.00274075 | 1.50E-23 |
| 130 | Chronotype | rs4784655 | G | C | 0.0211306 | 0.00288752 | 1.50E-13 |
| 131 | Chronotype | rs17604349 | G | A | 0.0257422 | 0.00351443 | 1.70E-13 |
| 132 | Chronotype | rs2518022 | T | C | 0.0403075 | 0.00485089 | 4.80E-17 |
| 133 | Chronotype | rs3760185 | C | T | 0.0241586 | 0.0031496 | 2.60E-14 |
| 134 | Chronotype | rs226090 | C | T | 0.0208783 | 0.00359523 | 4.10E-09 |
| 135 | Chronotype | rs28647842 | G | C | 0.0160844 | 0.00269439 | 2.00E-09 |
| 136 | Chronotype | rs72829706 | A | G | 0.0407112 | 0.00693598 | 5.10E-09 |
| 137 | Chronotype | rs114352914 | G | A | -0.0197917 | 0.00344154 | 7.90E-09 |
| 138 | Chronotype | rs2949923 | A | G | -0.016206 | 0.00270197 | 2.10E-09 |
| 139 | Chronotype | rs62082401 | C | G | -0.0256287 | 0.00342253 | 1.60E-14 |
| 140 | Chronotype | rs4239386 | T | A | 0.0218962 | 0.00285562 | 1.00E-14 |
| 141 | Chronotype | rs12969848 | C | T | -0.0218632 | 0.00270301 | 1.30E-15 |
| 142 | Chronotype | rs9962650 | C | G | -0.0173129 | 0.00272797 | 1.60E-10 |
| 143 | Chronotype | rs9964420 | C | A | 0.0284801 | 0.00293643 | 8.60E-22 |
| 144 | Chronotype | rs56076457 | C | T | -0.0162984 | 0.00269462 | 1.30E-09 |
| 145 | Chronotype | rs10402849 | C | T | -0.0192609 | 0.00335946 | 8.90E-09 |
| 146 | Chronotype | rs9636202 | G | A | 0.0183734 | 0.00305534 | 7.50E-10 |
| 147 | Chronotype | rs11670534 | C | T | 0.0209755 | 0.00362802 | 8.50E-09 |
| 148 | Chronotype | rs78095690 | T | C | -0.0163477 | 0.00272137 | 1.40E-09 |
| 149 | Chronotype | rs6131942 | A | G | -0.0175437 | 0.00273235 | 9.40E-11 |
| 150 | Chronotype | rs2072727 | T | C | 0.016352 | 0.00271514 | 8.50E-10 |
| 151 | Chronotype | rs139911 | C | T | 0.0233915 | 0.00273231 | 2.30E-17 |
| 152 | Chronotype | rs4822107 | G | A | -0.015528 | 0.00270621 | 9.60E-09 |

SNP, single nucleotide polymorphism; se, standard error

**Supplementary Table 2.** The summary information for instrumental variables of sleep duration.

| **Sort** | **Phenotype** | **SNP** | **effect_allele** | **other_allele** | **beta** |  | **se** | **pval** |
| --- | --- | --- | --- | --- | --- | --- | --- | --- |
| 1 | Sleep duration | rs915416 | C | G | 0.0192587 |  | 0.00249452 | 9.90E-15 |
| 2 | Sleep duration | rs269054 | T | A | -0.0136431 |  | 0.00229327 | 2.10E-09 |
| 3 | Sleep duration | rs61796569 | C | T | -0.0154442 |  | 0.00256443 | 1.50E-09 |
| 4 | Sleep duration | rs12567114 | G | A | -0.0148307 |  | 0.0025403 | 4.30E-09 |
| 5 | Sleep duration | rs62120041 | T | C | 0.0261113 |  | 0.00457497 | 9.60E-09 |
| 6 | Sleep duration | rs374153 | C | T | 0.0176119 |  | 0.00310308 | 9.10E-09 |
| 7 | Sleep duration | rs2717076 | C | T | -0.0184107 |  | 0.00234091 | 3.10E-15 |
| 8 | Sleep duration | rs75539574 | A | C | -0.0362482 |  | 0.00406522 | 6.90E-19 |
| 9 | Sleep duration | rs7556815 | G | A | -0.0407248 |  | 0.00274023 | 1.30E-49 |
| 10 | Sleep duration | rs35662245 | T | A | -0.0145968 |  | 0.00239263 | 1.40E-09 |
| 11 | Sleep duration | rs11885663 | C | T | -0.0162176 |  | 0.00261811 | 8.60E-10 |
| 12 | Sleep duration | rs10173260 | T | C | -0.0128368 |  | 0.00231322 | 2.90E-08 |
| 13 | Sleep duration | rs112230981 | A | G | 0.0315275 |  | 0.00522787 | 2.20E-09 |
| 14 | Sleep duration | rs17732997 | C | G | 0.0129345 |  | 0.0022882 | 1.20E-08 |
| 15 | Sleep duration | rs7644809 | T | C | 0.0130621 |  | 0.00230148 | 1.60E-08 |
| 16 | Sleep duration | rs13088093 | T | G | -0.0162722 |  | 0.00240235 | 7.00E-12 |
| 17 | Sleep duration | rs2192528 | A | G | 0.0133687 |  | 0.0022692 | 2.70E-09 |
| 18 | Sleep duration | rs17427571 | A | G | 0.0138255 |  | 0.00243544 | 1.30E-08 |
| 19 | Sleep duration | rs35531607 | T | C | -0.0128402 |  | 0.00227278 | 1.50E-08 |
| 20 | Sleep duration | rs13109404 | T | G | 0.0312035 |  | 0.00440829 | 1.40E-12 |
| 21 | Sleep duration | rs365663 | A | G | 0.0146291 |  | 0.00227857 | 1.00E-10 |
| 22 | Sleep duration | rs465700 | C | G | 0.0225148 |  | 0.00367362 | 1.40E-09 |
| 23 | Sleep duration | rs56372231 | C | T | -0.0169439 |  | 0.00239981 | 2.20E-12 |
| 24 | Sleep duration | rs11567976 | C | T | -0.0128042 |  | 0.00228516 | 2.10E-08 |
| 25 | Sleep duration | rs151014368 | G | A | -0.0160924 |  | 0.00281958 | 9.10E-09 |
| 26 | Sleep duration | rs34556183 | A | G | 0.0169228 |  | 0.00252323 | 2.30E-11 |
| 27 | Sleep duration | rs113113059 | T | C | 0.0161406 |  | 0.00273732 | 8.40E-09 |
| 28 | Sleep duration | rs9382445 | T | C | 0.014536 |  | 0.00233387 | 4.80E-10 |
| 29 | Sleep duration | rs2231265 | A | G | -0.014955 |  | 0.00269917 | 2.70E-08 |
| 30 | Sleep duration | rs9345234 | A | C | -0.0130117 |  | 0.00229893 | 1.80E-08 |
| 31 | Sleep duration | rs34731055 | C | T | -0.0194603 |  | 0.00294778 | 3.70E-11 |
| 32 | Sleep duration | rs2079070 | C | G | 0.0175475 |  | 0.00256638 | 7.50E-12 |
| 33 | Sleep duration | rs7806045 | T | C | 0.0147916 |  | 0.00262577 | 1.40E-08 |
| 34 | Sleep duration | rs4841498 | C | T | 0.0139954 |  | 0.00226922 | 1.20E-09 |
| 35 | Sleep duration | rs73219758 | G | A | 0.0164012 |  | 0.00249526 | 5.60E-11 |
| 36 | Sleep duration | rs10973207 | G | T | -0.0204339 |  | 0.00312394 | 6.00E-11 |
| 37 | Sleep duration | rs1776776 | T | C | 0.019963 |  | 0.00341071 | 4.90E-09 |
| 38 | Sleep duration | rs12246842 | A | G | 0.0133949 |  | 0.00227425 | 3.90E-09 |
| 39 | Sleep duration | rs10761674 | C | T | 0.0123329 |  | 0.00226591 | 4.20E-08 |
| 40 | Sleep duration | rs11190970 | G | A | 0.015379 |  | 0.00282318 | 4.60E-08 |
| 41 | Sleep duration | rs7915425 | T | C | 0.0190638 |  | 0.00298959 | 2.00E-10 |
| 42 | Sleep duration | rs1517572 | A | C | -0.0146443 |  | 0.00229467 | 1.50E-10 |
| 43 | Sleep duration | rs4592416 | A | G | -0.0146828 |  | 0.0022701 | 9.30E-11 |
| 44 | Sleep duration | rs11039544 | G | A | 0.018389 |  | 0.00307361 | 1.90E-09 |
| 45 | Sleep duration | rs174560 | T | C | -0.0135751 |  | 0.00243675 | 2.80E-08 |
| 46 | Sleep duration | rs12791153 | A | T | -0.0235481 |  | 0.0042169 | 1.90E-08 |
| 47 | Sleep duration | rs1553132 | A | G | -0.0145068 |  | 0.00258447 | 2.50E-08 |
| 48 | Sleep duration | rs1939455 | G | T | 0.0204253 |  | 0.00356118 | 1.20E-08 |
| 49 | Sleep duration | rs1079727 | T | C | -0.0182922 |  | 0.00310347 | 5.30E-09 |
| 50 | Sleep duration | rs3751046 | A | G | -0.0194129 |  | 0.00320818 | 1.10E-09 |
| 51 | Sleep duration | rs34354917 | C | A | 0.0137464 |  | 0.00250081 | 3.90E-08 |
| 52 | Sleep duration | rs4767550 | A | G | -0.0143001 |  | 0.0023096 | 6.30E-10 |
| 53 | Sleep duration | rs6575005 | T | C | 0.0155637 |  | 0.00264172 | 4.40E-09 |
| 54 | Sleep duration | rs10483350 | A | G | -0.017369 |  | 0.0028676 | 1.50E-09 |
| 55 | Sleep duration | rs61985058 | C | T | -0.0185938 |  | 0.00322893 | 1.30E-08 |
| 56 | Sleep duration | rs55658675 | C | T | 0.0131415 |  | 0.00236892 | 2.00E-08 |
| 57 | Sleep duration | rs11621908 | C | T | 0.0240951 |  | 0.00416298 | 5.60E-09 |
| 58 | Sleep duration | rs8038326 | A | G | 0.0159204 |  | 0.0025407 | 2.80E-10 |
| 59 | Sleep duration | rs3095508 | C | A | 0.0153518 |  | 0.00230437 | 3.10E-11 |
| 60 | Sleep duration | rs11643715 | C | G | -0.013895 |  | 0.00249658 | 3.20E-08 |
| 61 | Sleep duration | rs9937053 | G | A | 0.0169348 |  | 0.00229041 | 1.20E-13 |
| 62 | Sleep duration | rs7198661 | T | C | 0.0133829 |  | 0.00227449 | 3.90E-09 |
| 63 | Sleep duration | rs3027234 | C | T | 0.0151536 |  | 0.00270628 | 2.30E-08 |
| 64 | Sleep duration | rs205024 | C | T | -0.0138261 |  | 0.00232711 | 3.90E-09 |
| 65 | Sleep duration | rs8072993 | T | G | -0.0174776 |  | 0.00282169 | 4.20E-10 |
| 66 | Sleep duration | rs147114641 | C | A | 0.0159801 |  | 0.00270379 | 3.10E-09 |
| 67 | Sleep duration | rs2696429 | G | A | 0.0168884 |  | 0.00270763 | 4.00E-10 |
| 68 | Sleep duration | rs12607679 | T | C | 0.0201387 |  | 0.00259284 | 8.30E-15 |
| 69 | Sleep duration | rs10421649 | T | A | -0.0132975 |  | 0.00229462 | 6.90E-09 |
| 70 | Sleep duration | rs2072727 | T | C | 0.0132425 |  | 0.00228506 | 7.90E-09 |

**Supplementary Table 3.** The summary information for instrumental variables of insomnia.

| **Sort** | **Phenotype** | **SNP** | **effect_allele** | **other_allele** | **beta** | **se** | **pval** |
| --- | --- | --- | --- | --- | --- | --- | --- |
| 1 | Insomnia | rs113851554 | T | G | 0.206 | 0.206 | 1.56E-51 |
| 2 | Insomnia | rs9527083 | G | A | 0.076 | 0.006 | 1.61E-32 |
| 3 | Insomnia | rs10947428 | C | T | 0.068 | 0.007 | 9.06E-21 |
| 4 | Insomnia | rs6119267 | G | C | 0.06 | 0.006 | 2.32E-20 |
| 5 | Insomnia | rs62158170 | A | G | 0.066 | 0.007 | 1.20E-19 |
| 6 | Insomnia | rs55972276 | A | C | 0.073 | 0.009 | 4.19E-17 |
| 7 | Insomnia | rs2431108 | C | T | 0.053 | 0.006 | 7.83E-17 |
| 8 | Insomnia | rs8180817 | G | C | 0.049 | 0.006 | 1.83E-16 |
| 9 | Insomnia | rs118166957 | T | C | 0.068 | 0.008 | 1.95E-16 |
| 10 | Insomnia | rs13135092 | G | A | 0.089 | 0.011 | 2.53E-16 |
| 11 | Insomnia | rs35322724 | A | C | 0.049 | 0.006 | 3.75E-16 |
| 12 | Insomnia | rs4702 | G | A | 0.048 | 0.006 | 6.78E-16 |
| 13 | Insomnia | rs16903122 | T | C | 0.055 | 0.007 | 9.04E-16 |
| 14 | Insomnia | rs77641763 | T | C | 0.071 | 0.009 | 6.53E-15 |
| 15 | Insomnia | rs1927902 | T | C | 0.053 | 0.007 | 1.15E-14 |
| 16 | Insomnia | rs62264767 | A | C | 0.065 | 0.008 | 1.63E-14 |
| 17 | Insomnia | rs1620977 | A | G | 0.052 | 0.007 | 2.27E-14 |
| 18 | Insomnia | rs1015438 | A | G | 0.058 | 0.008 | 2.51E-14 |
| 19 | Insomnia | rs17643634 | C | T | 0.06 | 0.008 | 1.34E-13 |
| 20 | Insomnia | rs28582096 | G | A | 0.054 | 0.007 | 1.74E-13 |
| 21 | Insomnia | rs694786 | C | T | 0.044 | 0.006 | 1.97E-13 |
| 22 | Insomnia | rs12912299 | C | T | 0.043 | 0.006 | 4.42E-13 |
| 23 | Insomnia | rs7214267 | G | A | 0.044 | 0.006 | 5.09E-13 |
| 24 | Insomnia | rs314281 | C | T | 0.043 | 0.006 | 6.03E-13 |
| 25 | Insomnia | rs11605348 | G | A | 0.045 | 0.006 | 7.01E-13 |
| 26 | Insomnia | rs4981170 | G | A | 0.054 | 0.008 | 7.33E-13 |
| 27 | Insomnia | rs9931543 | T | C | 0.048 | 0.007 | 1.11E-12 |
| 28 | Insomnia | rs524859 | G | A | 0.044 | 0.006 | 1.48E-12 |
| 29 | Insomnia | rs72657797 | C | T | 0.056 | 0.008 | 1.52E-12 |
| 30 | Insomnia | rs60565673 | G | T | 0.043 | 0.006 | 1.59E-12 |
| 31 | Insomnia | rs10761240 | G | A | 0.043 | 0.006 | 2.12E-12 |
| 32 | Insomnia | rs66674044 | T | A | 0.06 | 0.009 | 2.18E-12 |
| 33 | Insomnia | rs12666306 | A | G | 0.042 | 0.006 | 2.24E-12 |
| 34 | Insomnia | rs10800992 | T | C | 0.042 | 0.006 | 3.84E-12 |
| 35 | Insomnia | rs1031654 | C | A | 0.051 | 0.007 | 3.88E-12 |
| 36 | Insomnia | rs10947690 | G | A | 0.047 | 0.007 | 4.04E-12 |
| 37 | Insomnia | rs9889282 | C | A | 0.042 | 0.006 | 4.70E-12 |
| 38 | Insomnia | rs7044885 | G | C | 0.041 | 0.006 | 5.67E-12 |
| 39 | Insomnia | rs3774751 | G | T | 0.041 | 0.006 | 7.32E-12 |
| 40 | Insomnia | rs61921611 | C | T | 0.044 | 0.006 | 7.84E-12 |
| 41 | Insomnia | rs715338 | A | G | 0.041 | 0.006 | 7.85E-12 |
| 42 | Insomnia | rs830716 | C | G | 0.045 | 0.007 | 8.68E-12 |
| 43 | Insomnia | rs13010288 | G | T | 0.06 | 0.009 | 9.26E-12 |
| 44 | Insomnia | rs12983032 | G | A | 0.043 | 0.006 | 1.07E-11 |
| 45 | Insomnia | rs8180457 | C | T | 0.056 | 0.008 | 1.12E-11 |
| 46 | Insomnia | rs908668 | T | C | 0.05 | 0.007 | 1.41E-11 |
| 47 | Insomnia | rs79693059 | G | C | 0.073 | 0.011 | 1.61E-11 |
| 48 | Insomnia | rs12991815 | C | G | 0.04 | 0.006 | 3.02E-11 |
| 49 | Insomnia | rs12310246 | A | G | 0.045 | 0.007 | 4.74E-11 |
| 50 | Insomnia | rs55772859 | A | C | 0.042 | 0.006 | 4.82E-11 |
| 51 | Insomnia | rs6808140 | T | C | 0.039 | 0.006 | 5.35E-11 |
| 52 | Insomnia | rs2286729 | A | G | 0.07 | 0.011 | 5.37E-11 |
| 53 | Insomnia | rs11803128 | G | A | 0.041 | 0.006 | 6.85E-11 |
| 54 | Insomnia | rs28611339 | T | G | 0.058 | 0.009 | 8.46E-11 |
| 55 | Insomnia | rs35110063 | A | G | 0.039 | 0.006 | 8.82E-11 |
| 56 | Insomnia | rs6888135 | A | C | 0.038 | 0.006 | 1.21E-10 |
| 57 | Insomnia | rs6562066 | T | C | 0.039 | 0.006 | 1.38E-10 |
| 58 | Insomnia | rs6967168 | G | T | 0.044 | 0.007 | 1.39E-10 |
| 59 | Insomnia | rs2389631 | C | A | 0.04 | 0.006 | 2.03E-10 |
| 60 | Insomnia | rs116466468 | T | C | 0.044 | 0.007 | 2.11E-10 |
| 61 | Insomnia | rs1064939 | A | T | 0.13 | 0.02 | 2.16E-10 |
| 62 | Insomnia | rs17223714 | A | G | 0.046 | 0.007 | 2.44E-10 |
| 63 | Insomnia | rs1038093 | T | C | 0.039 | 0.006 | 2.47E-10 |
| 64 | Insomnia | rs224029 | C | T | 0.039 | 0.006 | 2.51E-10 |
| 65 | Insomnia | rs2089358 | C | T | 0.041 | 0.007 | 2.75E-10 |
| 66 | Insomnia | rs8076183 | C | T | 0.038 | 0.006 | 2.75E-10 |
| 67 | Insomnia | rs671985 | G | A | 0.038 | 0.006 | 2.79E-10 |
| 68 | Insomnia | rs12251016 | T | A | 0.039 | 0.006 | 3.89E-10 |
| 69 | Insomnia | rs4592425 | T | G | 0.04 | 0.006 | 4.31E-10 |
| 70 | Insomnia | rs823247 | C | T | 0.037 | 0.006 | 5.25E-10 |
| 71 | Insomnia | rs73671843 | G | A | 0.056 | 0.009 | 5.49E-10 |
| 72 | Insomnia | rs17005118 | A | G | 0.042 | 0.007 | 6.13E-10 |
| 73 | Insomnia | rs4767645 | G | T | 0.037 | 0.006 | 6.47E-10 |
| 74 | Insomnia | rs6019663 | T | C | 0.04 | 0.007 | 6.47E-10 |
| 75 | Insomnia | rs4502882 | C | T | 0.039 | 0.006 | 7.96E-10 |
| 76 | Insomnia | rs1530938 | A | G | 0.036 | 0.006 | 8.82E-10 |
| 77 | Insomnia | rs3184470 | G | A | 0.038 | 0.006 | 9.73E-10 |
| 78 | Insomnia | rs1147852 | A | G | 0.039 | 0.006 | 9.94E-10 |
| 79 | Insomnia | rs72899452 | T | C | 0.074 | 0.012 | 1.00E-09 |
| 80 | Insomnia | rs6756610 | C | G | 0.037 | 0.006 | 1.14E-09 |
| 81 | Insomnia | rs62068188 | T | C | 0.049 | 0.008 | 1.18E-09 |
| 82 | Insomnia | rs6702604 | G | A | 0.037 | 0.006 | 1.30E-09 |
| 83 | Insomnia | rs2388840 | G | A | 0.037 | 0.006 | 1.37E-09 |
| 84 | Insomnia | rs324017 | A | C | 0.039 | 0.007 | 1.61E-09 |
| 85 | Insomnia | rs12520974 | C | T | 0.036 | 0.006 | 1.69E-09 |
| 86 | Insomnia | rs62429521 | A | C | 0.051 | 0.008 | 1.78E-09 |
| 87 | Insomnia | rs11090039 | A | G | 0.039 | 0.007 | 1.82E-09 |
| 88 | Insomnia | rs6734957 | G | T | 0.042 | 0.007 | 1.82E-09 |
| 89 | Insomnia | rs6465151 | T | C | 0.056 | 0.009 | 1.90E-09 |
| 90 | Insomnia | rs2221119 | C | G | 0.036 | 0.006 | 2.00E-09 |
| 91 | Insomnia | rs12605642 | T | G | 0.035 | 0.006 | 2.13E-09 |
| 92 | Insomnia | rs11149313 | A | G | 0.04 | 0.007 | 2.38E-09 |
| 93 | Insomnia | rs2598293 | T | C | 0.035 | 0.006 | 2.48E-09 |
| 94 | Insomnia | rs4709655 | C | T | 0.054 | 0.009 | 3.09E-09 |
| 95 | Insomnia | rs214934 | T | A | 0.038 | 0.006 | 3.16E-09 |
| 96 | Insomnia | rs492858 | C | T | 0.066 | 0.011 | 3.46E-09 |
| 97 | Insomnia | rs7040224 | A | G | 0.037 | 0.006 | 4.24E-09 |
| 98 | Insomnia | rs34967082 | A | G | 0.035 | 0.006 | 4.34E-09 |
| 99 | Insomnia | rs35539975 | A | G | 0.042 | 0.007 | 4.49E-09 |
| 100 | Insomnia | rs6589988 | G | A | 0.038 | 0.006 | 4.70E-09 |
| 101 | Insomnia | rs10758593 | G | A | 0.036 | 0.006 | 4.90E-09 |
| 102 | Insomnia | rs4664299 | C | T | 0.041 | 0.007 | 4.95E-09 |
| 103 | Insomnia | rs11588755 | G | A | 0.035 | 0.006 | 5.14E-09 |
| 104 | Insomnia | rs7402939 | C | T | 0.036 | 0.006 | 5.19E-09 |
| 105 | Insomnia | rs871994 | A | C | 0.035 | 0.006 | 5.50E-09 |
| 106 | Insomnia | rs1289939 | C | T | 0.041 | 0.007 | 6.00E-09 |
| 107 | Insomnia | rs1536053 | C | T | 0.038 | 0.006 | 6.04E-09 |
| 108 | Insomnia | rs4788203 | G | A | 0.035 | 0.006 | 6.32E-09 |
| 109 | Insomnia | rs7475916 | G | C | 0.037 | 0.006 | 6.70E-09 |
| 110 | Insomnia | rs701394 | G | A | 0.036 | 0.006 | 6.83E-09 |
| 111 | Insomnia | rs2838787 | G | A | 0.036 | 0.006 | 7.65E-09 |
| 112 | Insomnia | rs10944696 | G | A | 0.038 | 0.007 | 7.99E-09 |
| 113 | Insomnia | rs10898940 | A | C | 0.034 | 0.006 | 8.09E-09 |
| 114 | Insomnia | rs12030482 | A | T | 0.041 | 0.007 | 8.16E-09 |
| 115 | Insomnia | rs1167132 | T | C | 0.035 | 0.006 | 8.73E-09 |
| 116 | Insomnia | rs17367725 | C | T | 0.036 | 0.006 | 9.29E-09 |
| 117 | Insomnia | rs176644 | T | G | 0.035 | 0.006 | 9.49E-09 |
| 118 | Insomnia | rs728017 | G | A | 0.035 | 0.006 | 9.51E-09 |
| 119 | Insomnia | rs2030672 | C | G | 0.034 | 0.006 | 1.10E-08 |
| 120 | Insomnia | rs11119409 | C | T | 0.035 | 0.006 | 1.19E-08 |
| 121 | Insomnia | rs4858708 | T | A | 0.034 | 0.006 | 1.23E-08 |
| 122 | Insomnia | rs5877 | T | C | 0.036 | 0.006 | 1.23E-08 |
| 123 | Insomnia | rs72820274 | A | G | 0.034 | 0.006 | 1.28E-08 |
| 124 | Insomnia | rs11756035 | C | G | 0.051 | 0.009 | 1.29E-08 |
| 125 | Insomnia | rs1731951 | T | A | 0.035 | 0.006 | 1.36E-08 |
| 126 | Insomnia | rs62301574 | G | C | 0.042 | 0.007 | 1.37E-08 |
| 127 | Insomnia | rs7571486 | G | A | 0.039 | 0.007 | 1.40E-08 |
| 128 | Insomnia | rs521484 | G | A | 0.04 | 0.007 | 1.53E-08 |
| 129 | Insomnia | rs4588900 | A | G | 0.033 | 0.006 | 1.57E-08 |
| 130 | Insomnia | rs17083297 | C | A | 0.044 | 0.008 | 1.60E-08 |
| 131 | Insomnia | rs2216427 | C | G | 0.035 | 0.006 | 1.60E-08 |
| 132 | Insomnia | rs910187 | G | A | 0.035 | 0.006 | 1.63E-08 |
| 133 | Insomnia | rs34490907 | C | G | 0.054 | 0.009 | 1.76E-08 |
| 134 | Insomnia | rs10756571 | T | C | 0.036 | 0.006 | 1.80E-08 |
| 135 | Insomnia | rs10955647 | T | G | 0.033 | 0.006 | 1.84E-08 |
| 136 | Insomnia | rs13138995 | A | G | 0.034 | 0.006 | 1.97E-08 |
| 137 | Insomnia | rs16990210 | C | T | 0.046 | 0.008 | 1.97E-08 |
| 138 | Insomnia | rs2364921 | C | T | 0.034 | 0.006 | 2.13E-08 |
| 139 | Insomnia | rs769449 | G | A | 0.046 | 0.008 | 2.13E-08 |
| 140 | Insomnia | rs1567084 | A | G | 0.033 | 0.006 | 2.14E-08 |
| 141 | Insomnia | rs17025198 | A | G | 0.041 | 0.007 | 2.19E-08 |
| 142 | Insomnia | rs6601080 | A | G | 0.035 | 0.006 | 2.21E-08 |
| 143 | Insomnia | rs638746 | A | G | 0.033 | 0.006 | 2.26E-08 |
| 144 | Insomnia | rs1580173 | A | G | 0.033 | 0.006 | 2.28E-08 |
| 145 | Insomnia | rs742760 | A | T | 0.043 | 0.008 | 2.48E-08 |
| 146 | Insomnia | rs11001276 | T | A | 0.038 | 0.007 | 2.52E-08 |
| 147 | Insomnia | rs76145129 | G | T | 0.05 | 0.009 | 2.73E-08 |
| 148 | Insomnia | rs17520265 | G | A | 0.091 | 0.016 | 2.87E-08 |
| 149 | Insomnia | rs647905 | T | C | 0.033 | 0.006 | 2.87E-08 |
| 150 | Insomnia | rs28552587 | A | G | 0.033 | 0.006 | 3.30E-08 |
| 151 | Insomnia | rs62383308 | G | A | 0.06 | 0.011 | 3.98E-08 |
| 152 | Insomnia | rs699844 | A | G | 0.06 | 0.011 | 4.11E-08 |
| 153 | Insomnia | rs6973090 | G | A | 0.038 | 0.007 | 4.31E-08 |
| 154 | Insomnia | rs9964420 | A | C | 0.035 | 0.007 | 4.54E-08 |
| 155 | Insomnia | rs6510033 | G | A | 0.037 | 0.007 | 4.66E-08 |

**Supplementary Table 4.** The summary information for instrumental variables of daytime sleepiness.

| Sort | Phenotype | SNP | effect_allele | other_allele | beta | se | pval |
| --- | --- | --- | --- | --- | --- | --- | --- |
| 1 | Daytime sleepiness | rs2787120 | A | G | 0.00778398 | 0.00137756 | 2.00E-08 |
| 2 | Daytime sleepiness | rs12140153 | G | T | 0.01658 | 0.00179639 | 2.80E-20 |
| 3 | Daytime sleepiness | rs501701 | A | G | 0.0060623 | 0.00103722 | 6.50E-09 |
| 4 | Daytime sleepiness | rs17131124 | C | G | -0.0111768 | 0.00183679 | 1.70E-09 |
| 5 | Daytime sleepiness | rs57746981 | C | T | 0.00680998 | 0.00107584 | 2.20E-10 |
| 6 | Daytime sleepiness | rs825127 | T | G | 0.00591296 | 0.00103275 | 9.50E-09 |
| 7 | Daytime sleepiness | rs4665972 | T | C | 0.0066245 | 0.00105853 | 3.90E-10 |
| 8 | Daytime sleepiness | rs7598712 | G | T | 0.00579387 | 0.00104191 | 2.20E-08 |
| 9 | Daytime sleepiness | rs6741951 | G | A | 0.00681617 | 0.00114107 | 2.70E-09 |
| 10 | Daytime sleepiness | rs11123962 | T | G | -0.00804433 | 0.00103439 | 7.50E-15 |
| 11 | Daytime sleepiness | rs9712275 | C | T | -0.00588405 | 0.0010319 | 1.30E-08 |
| 12 | Daytime sleepiness | rs7607363 | A | G | -0.0060244 | 0.00103841 | 8.00E-09 |
| 13 | Daytime sleepiness | rs13010456 | A | G | 0.00774142 | 0.00105125 | 2.10E-13 |
| 14 | Daytime sleepiness | rs13097760 | A | C | -0.00598755 | 0.00107776 | 3.20E-08 |
| 15 | Daytime sleepiness | rs960986 | C | T | 0.00716034 | 0.00106799 | 1.50E-11 |
| 16 | Daytime sleepiness | rs843372 | C | T | 0.00816368 | 0.00122597 | 2.20E-11 |
| 17 | Daytime sleepiness | rs11942333 | G | A | -0.00605348 | 0.00110327 | 3.80E-08 |
| 18 | Daytime sleepiness | rs13135092 | A | G | -0.0103303 | 0.0018774 | 3.10E-08 |
| 19 | Daytime sleepiness | rs6897863 | A | C | 0.00645716 | 0.00104732 | 7.60E-10 |
| 20 | Daytime sleepiness | rs12153518 | A | G | 0.00670287 | 0.00103166 | 6.80E-11 |
| 21 | Daytime sleepiness | rs6923811 | T | C | 0.00677376 | 0.00110514 | 9.10E-10 |
| 22 | Daytime sleepiness | rs55960940 | T | C | 0.00762668 | 0.00135164 | 2.00E-08 |
| 23 | Daytime sleepiness | rs3122170 | C | A | 0.00950345 | 0.00122688 | 5.60E-15 |
| 24 | Daytime sleepiness | rs62519825 | T | C | -0.0094794 | 0.00162829 | 3.80E-09 |
| 25 | Daytime sleepiness | rs285793 | G | A | 0.00676457 | 0.00103539 | 7.90E-11 |
| 26 | Daytime sleepiness | rs7837226 | A | G | -0.00574201 | 0.00103269 | 2.00E-08 |
| 27 | Daytime sleepiness | rs55818482 | T | C | -0.00973113 | 0.00125942 | 1.40E-14 |
| 28 | Daytime sleepiness | rs1566362 | T | C | 0.00631993 | 0.0010687 | 3.80E-09 |
| 29 | Daytime sleepiness | rs7476897 | G | A | 0.00743007 | 0.00110192 | 2.70E-11 |
| 30 | Daytime sleepiness | rs4765939 | G | C | -0.00627707 | 0.00104601 | 2.00E-09 |
| 31 | Daytime sleepiness | rs1846644 | T | C | -0.0113546 | 0.00104777 | 2.50E-27 |
| 32 | Daytime sleepiness | rs8015449 | A | G | 0.00619154 | 0.00103462 | 1.90E-09 |
| 33 | Daytime sleepiness | rs17356118 | A | G | -0.00765559 | 0.00122087 | 2.60E-10 |
| 34 | Daytime sleepiness | rs886114 | C | T | 0.00604278 | 0.00107429 | 1.90E-08 |
| 35 | Daytime sleepiness | rs11078398 | G | A | 0.00766031 | 0.00124122 | 7.10E-10 |
| 36 | Daytime sleepiness | rs147114641 | C | A | 0.00787842 | 0.00122983 | 1.70E-10 |
| 37 | Daytime sleepiness | rs62055936 | T | A | 0.00819979 | 0.00124114 | 4.40E-11 |
| 38 | Daytime sleepiness | rs2048522 | A | T | 0.00581823 | 0.00105065 | 3.50E-08 |

**Supplementary Table 5.** The summary information for instrumental variables of daytime napping.

| **Sort** | **Phenotype** | **SNP** | **effect_allele** | **other_allele** | **beta** | **se** | **pval** |
| --- | --- | --- | --- | --- | --- | --- | --- |
| 1 | Daytime napping | rs35570980 | G | A | -0.00777089 | 0.00123613 | 1.40E-10 |
| 2 | Daytime napping | rs2786547 | C | T | 0.0107673 | 0.00158775 | 1.00E-11 |
| 3 | Daytime napping | rs4653052 | C | A | -0.00703973 | 0.00128049 | 3.40E-08 |
| 4 | Daytime napping | rs12140153 | G | T | 0.0247147 | 0.00211564 | 2.40E-31 |
| 5 | Daytime napping | rs1931175 | C | G | -0.00776325 | 0.00124756 | 4.60E-10 |
| 6 | Daytime napping | rs12042846 | T | C | -0.00901497 | 0.00159014 | 1.90E-08 |
| 7 | Daytime napping | rs6665690 | A | C | 0.00923907 | 0.00161461 | 6.00E-09 |
| 8 | Daytime napping | rs35039375 | A | G | -0.0136431 | 0.00210973 | 8.70E-11 |
| 9 | Daytime napping | rs200435329 | G | T | -0.00986281 | 0.0014379 | 9.90E-12 |
| 10 | Daytime napping | rs2250377 | A | G | 0.0131561 | 0.00127975 | 1.40E-24 |
| 11 | Daytime napping | rs13033444 | A | G | -0.00974179 | 0.00135018 | 3.60E-13 |
| 12 | Daytime napping | rs75022160 | C | T | 0.00988485 | 0.00176929 | 1.70E-08 |
| 13 | Daytime napping | rs9309116 | C | T | -0.00737914 | 0.00127277 | 3.90E-09 |
| 14 | Daytime napping | rs2202323 | G | C | 0.00758702 | 0.0012578 | 1.20E-09 |
| 15 | Daytime napping | rs350785 | T | C | 0.0126479 | 0.00190975 | 1.50E-11 |
| 16 | Daytime napping | rs11682175 | T | C | -0.0069583 | 0.0012166 | 1.10E-08 |
| 17 | Daytime napping | rs7423968 | A | G | 0.00767586 | 0.00123216 | 4.80E-10 |
| 18 | Daytime napping | rs9287862 | C | T | 0.0122526 | 0.00215162 | 2.00E-08 |
| 19 | Daytime napping | rs62189006 | A | G | 0.0120943 | 0.0020982 | 8.30E-09 |
| 20 | Daytime napping | rs80163246 | T | C | -0.0119129 | 0.00188438 | 1.40E-10 |
| 21 | Daytime napping | rs12992648 | A | G | 0.00765323 | 0.00134659 | 2.40E-08 |
| 22 | Daytime napping | rs7422655 | C | T | 0.00783348 | 0.00137827 | 1.10E-08 |
| 23 | Daytime napping | rs908442 | A | T | 0.0102781 | 0.00123558 | 1.40E-16 |
| 24 | Daytime napping | rs77154532 | A | G | 0.00769324 | 0.0012705 | 1.40E-09 |
| 25 | Daytime napping | rs936944 | G | A | 0.00913528 | 0.00164374 | 3.20E-08 |
| 26 | Daytime napping | rs76824303 | A | C | 0.0117977 | 0.00207749 | 1.70E-08 |
| 27 | Daytime napping | rs4511908 | T | G | -0.00726347 | 0.00128337 | 1.40E-08 |
| 28 | Daytime napping | rs1601440 | C | T | 0.00912653 | 0.00135575 | 1.90E-11 |
| 29 | Daytime napping | rs2699869 | A | C | 0.00678084 | 0.00121774 | 3.10E-08 |
| 30 | Daytime napping | rs253666 | G | A | -0.0081271 | 0.0014309 | 1.80E-08 |
| 31 | Daytime napping | rs1001817 | C | T | 0.00775426 | 0.00121418 | 1.70E-10 |
| 32 | Daytime napping | rs7697461 | A | T | -0.0082009 | 0.00139778 | 6.00E-09 |
| 33 | Daytime napping | rs13150944 | A | G | -0.00860076 | 0.0013218 | 6.80E-11 |
| 34 | Daytime napping | rs4692709 | C | T | 0.00723975 | 0.00122517 | 2.40E-09 |
| 35 | Daytime napping | rs12657723 | C | T | -0.00818319 | 0.00129849 | 2.00E-10 |
| 36 | Daytime napping | rs6452787 | A | G | 0.00772931 | 0.0012169 | 2.00E-10 |
| 37 | Daytime napping | rs2943023 | C | T | 0.00710422 | 0.00123055 | 7.40E-09 |
| 38 | Daytime napping | rs467897 | G | A | 0.00955698 | 0.00130107 | 2.60E-13 |
| 39 | Daytime napping | rs2431108 | T | C | -0.0129827 | 0.00129028 | 7.70E-24 |
| 40 | Daytime napping | rs2099810 | A | G | 0.00769192 | 0.00121447 | 2.80E-10 |
| 41 | Daytime napping | rs10875606 | C | A | 0.00737707 | 0.0013077 | 1.30E-08 |
| 42 | Daytime napping | rs10875622 | G | A | -0.0104621 | 0.0012291 | 1.30E-17 |
| 43 | Daytime napping | rs73817091 | C | T | -0.0165127 | 0.00302224 | 3.80E-08 |
| 44 | Daytime napping | rs3799380 | T | C | 0.00890214 | 0.00151371 | 1.30E-08 |
| 45 | Daytime napping | rs34262487 | C | A | 0.0145107 | 0.00235463 | 1.20E-09 |
| 46 | Daytime napping | rs6919087 | T | G | 0.0107806 | 0.0013114 | 1.00E-16 |
| 47 | Daytime napping | rs9475168 | T | G | 0.00876859 | 0.00147675 | 1.90E-09 |
| 48 | Daytime napping | rs2653349 | A | G | 0.0165942 | 0.00147827 | 3.40E-29 |
| 49 | Daytime napping | rs2143792 | G | A | 0.00713406 | 0.00123401 | 9.10E-09 |
| 50 | Daytime napping | rs1546977 | A | G | -0.00827718 | 0.00122321 | 1.00E-11 |
| 51 | Daytime napping | rs9389556 | C | G | -0.00841517 | 0.00138285 | 9.70E-10 |
| 52 | Daytime napping | rs785145 | T | G | -0.0069889 | 0.00122527 | 1.00E-08 |
| 53 | Daytime napping | rs614987 | A | C | -0.0110217 | 0.00124925 | 7.50E-19 |
| 54 | Daytime napping | rs140506252 | A | T | 0.0227261 | 0.0040886 | 4.30E-08 |
| 55 | Daytime napping | rs9460110 | T | C | -0.00735568 | 0.00125751 | 8.40E-09 |
| 56 | Daytime napping | rs35851551 | A | G | 0.011194 | 0.00202654 | 3.50E-08 |
| 57 | Daytime napping | rs10257273 | A | T | 0.0107366 | 0.00170413 | 8.90E-10 |
| 58 | Daytime napping | rs76257331 | G | A | -0.00867666 | 0.0015218 | 1.20E-08 |
| 59 | Daytime napping | rs13263535 | G | T | 0.00687135 | 0.00121641 | 1.60E-08 |
| 60 | Daytime napping | rs351776 | A | C | -0.00755367 | 0.00122072 | 8.40E-10 |
| 61 | Daytime napping | rs2059639 | C | T | 0.0066823 | 0.0012172 | 2.40E-08 |
| 62 | Daytime napping | rs285815 | T | A | 0.00753009 | 0.00121983 | 5.80E-10 |
| 63 | Daytime napping | rs7814873 | C | T | 0.00720972 | 0.00126385 | 1.50E-08 |
| 64 | Daytime napping | rs10811438 | G | C | 0.00721671 | 0.0012428 | 3.20E-09 |
| 65 | Daytime napping | rs17502738 | T | C | 0.00870459 | 0.00153004 | 2.00E-08 |
| 66 | Daytime napping | rs13284688 | T | C | -0.0150717 | 0.00149894 | 1.70E-23 |
| 67 | Daytime napping | rs295278 | G | A | 0.00787804 | 0.00124238 | 2.20E-10 |
| 68 | Daytime napping | rs971415 | A | G | 0.0110652 | 0.00185108 | 1.20E-09 |
| 69 | Daytime napping | rs4604518 | G | A | 0.00691205 | 0.00122091 | 1.40E-08 |
| 70 | Daytime napping | rs12346996 | T | C | 0.00796106 | 0.00136459 | 5.00E-09 |
| 71 | Daytime napping | rs11258652 | C | A | 0.0104004 | 0.00142918 | 3.70E-13 |
| 72 | Daytime napping | rs224111 | G | A | 0.00798411 | 0.00124912 | 1.60E-10 |
| 73 | Daytime napping | rs10840017 | A | G | 0.00894142 | 0.00148101 | 2.00E-09 |
| 74 | Daytime napping | rs10835420 | T | A | 0.00889802 | 0.00140206 | 1.70E-10 |
| 75 | Daytime napping | rs174541 | T | C | -0.00988214 | 0.00126293 | 4.40E-15 |
| 76 | Daytime napping | rs7932966 | A | T | 0.00872023 | 0.00142689 | 1.10E-09 |
| 77 | Daytime napping | rs530669754 | A | G | 0.0121029 | 0.00189146 | 1.60E-10 |
| 78 | Daytime napping | rs2417268 | A | T | -0.00752263 | 0.00123523 | 1.20E-09 |
| 79 | Daytime napping | rs35011311 | G | T | 0.009082 | 0.00138249 | 4.40E-11 |
| 80 | Daytime napping | rs60222088 | C | A | 0.0112358 | 0.00172293 | 6.40E-11 |
| 81 | Daytime napping | rs11615756 | C | T | -0.0182673 | 0.00123883 | 1.40E-49 |
| 82 | Daytime napping | rs2769916 | G | A | -0.00875328 | 0.00131344 | 1.70E-11 |
| 83 | Daytime napping | rs10149986 | T | G | -0.0109384 | 0.00156608 | 4.40E-12 |
| 84 | Daytime napping | rs2370926 | T | C | 0.00813213 | 0.00126359 | 1.70E-10 |
| 85 | Daytime napping | rs11071755 | G | A | 0.00711401 | 0.00122819 | 5.20E-09 |
| 86 | Daytime napping | rs17158413 | G | A | -0.00925014 | 0.00142955 | 4.40E-11 |
| 87 | Daytime napping | rs10152428 | G | C | -0.00780688 | 0.00136996 | 1.10E-08 |
| 88 | Daytime napping | rs60920123 | G | A | 0.00763778 | 0.00122697 | 4.50E-10 |
| 89 | Daytime napping | rs9939355 | C | T | 0.00726404 | 0.00122438 | 4.50E-09 |
| 90 | Daytime napping | rs378421 | G | A | -0.00697888 | 0.0012384 | 2.40E-08 |
| 91 | Daytime napping | rs11860072 | C | T | 0.00897773 | 0.00123952 | 2.80E-13 |
| 92 | Daytime napping | rs528301822 | A | T | -0.00803435 | 0.00134223 | 2.80E-09 |
| 93 | Daytime napping | rs12451365 | T | C | -0.010628 | 0.00150388 | 1.50E-12 |
| 94 | Daytime napping | rs385199 | A | C | 0.0209131 | 0.0014497 | 7.50E-47 |
| 95 | Daytime napping | rs112520848 | G | C | -0.00702449 | 0.00125191 | 1.80E-08 |
| 96 | Daytime napping | rs3935190 | G | A | -0.00801432 | 0.00122354 | 5.40E-11 |
| 97 | Daytime napping | rs962247 | G | A | 0.00796355 | 0.00122493 | 5.70E-11 |
| 98 | Daytime napping | rs2033103 | C | T | -0.00725728 | 0.00122022 | 2.60E-09 |
| 99 | Daytime napping | rs9965170 | G | A | 0.0136374 | 0.00122874 | 7.80E-29 |
| 100 | Daytime napping | rs17265513 | T | C | -0.00913188 | 0.00151965 | 2.00E-09 |
| 101 | Daytime napping | rs910187 | G | A | 0.00731754 | 0.00125594 | 4.90E-09 |
| 102 | Daytime napping | rs3810484 | A | G | 0.00684147 | 0.001222 | 2.20E-08 |
| 103 | Daytime napping | rs1883048 | T | C | -0.00787143 | 0.00122313 | 1.80E-10 |
| 104 | Daytime napping | rs2284015 | C | G | -0.00771167 | 0.00138662 | 4.10E-08 |

**Supplementary Table 6.** SNPs as instrumental variables in the study of the effect of sleep traits on CRC risk.

| **Exposure** | **SNP** | **Effect  allele** | **Other  allele** | **Exposure** | | | **Outcome** | | |
| --- | --- | --- | --- | --- | --- | --- | --- | --- | --- |
|  |  |  |  | **Beta** | **SE** | **P value** | **Beta** | **SE** | **P value** |
| Chronotype | rs10067113 | C | T | -0.016 | 0.003 | 6.30E-09 | -0.015 | 0.019 | 0.439 |
|  | rs10149448 | A | G | 0.016 | 0.003 | 2.70E-09 | 0.020 | 0.018 | 0.263 |
|  | rs1027742 | A | G | 0.018 | 0.003 | 2.80E-09 | 0.003 | 0.020 | 0.868 |
|  | rs10280205 | T | C | 0.017 | 0.003 | 1.40E-09 | 0.014 | 0.019 | 0.449 |
|  | rs10402849 | C | T | -0.019 | 0.003 | 8.90E-09 | 0.009 | 0.020 | 0.645 |
|  | rs10495976 | A | T | -0.018 | 0.003 | 5.10E-11 | -0.002 | 0.018 | 0.928 |
|  | rs10520176 | T | C | 0.023 | 0.003 | 1.40E-17 | -0.005 | 0.018 | 0.792 |
|  | rs10520176 | T | C | 0.023 | 0.003 | 1.40E-17 | 0.066 | 0.438 | 0.881 |
|  | rs1064213 | G | A | -0.019 | 0.003 | 1.30E-12 | -0.026 | 0.018 | 0.163 |
|  | rs10742179 | A | G | 0.018 | 0.003 | 1.50E-09 | -0.015 | 0.021 | 0.459 |
|  | rs10823233 | T | C | -0.015 | 0.003 | 4.30E-08 | 0.005 | 0.018 | 0.787 |
|  | rs10988239 | C | T | 0.019 | 0.003 | 1.60E-11 | -0.002 | 0.018 | 0.905 |
|  | rs11032362 | G | A | -0.040 | 0.005 | 4.80E-17 | -0.010 | 0.039 | 0.805 |
|  | rs1113295 | T | G | 0.016 | 0.003 | 6.80E-09 | -0.012 | 0.018 | 0.495 |
|  | rs11227452 | G | A | -0.019 | 0.004 | 4.60E-08 | 0.067 | 0.023 | 0.004 |
|  | rs114352914 | G | A | -0.020 | 0.003 | 7.90E-09 | -0.046 | 0.022 | 0.035 |
|  | rs114670539 | C | T | -0.034 | 0.006 | 4.60E-09 | -0.064 | 0.055 | 0.244 |
|  | rs114848860 | A | T | -0.056 | 0.009 | 3.60E-10 | 0.086 | 0.056 | 0.125 |
|  | rs114870822 | G | A | -0.070 | 0.012 | 2.00E-09 | 0.009 | 0.082 | 0.915 |
|  | rs11587758 | G | A | -0.024 | 0.003 | 5.50E-19 | -0.007 | 0.018 | 0.692 |
|  | rs11588913 | G | A | 0.015 | 0.003 | 1.50E-08 | 0.012 | 0.019 | 0.529 |
|  | rs11670534 | C | T | 0.021 | 0.004 | 8.50E-09 | 0.025 | 0.027 | 0.355 |
|  | rs11712056 | T | C | 0.020 | 0.003 | 1.60E-13 | -0.026 | 0.019 | 0.173 |
|  | rs11748798 | G | T | 0.020 | 0.003 | 5.20E-11 | 0.024 | 0.021 | 0.245 |
|  | rs11786306 | G | C | -0.018 | 0.003 | 8.70E-11 | -0.018 | 0.018 | 0.318 |
|  | rs11924337 | G | C | -0.019 | 0.003 | 2.20E-10 | 0.033 | 0.024 | 0.166 |
|  | rs12140153 | G | T | 0.034 | 0.005 | 1.60E-13 | -0.046 | 0.034 | 0.176 |
|  | rs12432176 | C | A | -0.016 | 0.003 | 6.50E-09 | -0.008 | 0.018 | 0.657 |
|  | rs12541362 | A | T | -0.021 | 0.003 | 3.50E-14 | -0.020 | 0.019 | 0.309 |
|  | rs12811046 | A | G | 0.017 | 0.003 | 6.20E-10 | 0.010 | 0.018 | 0.575 |
|  | rs12927162 | A | G | 0.029 | 0.003 | 6.80E-22 | 0.026 | 0.019 | 0.177 |
|  | rs12969848 | C | T | -0.022 | 0.003 | 1.30E-15 | -0.026 | 0.018 | 0.158 |
|  | rs13065394 | G | T | 0.018 | 0.003 | 3.00E-09 | 0.020 | 0.020 | 0.322 |
|  | rs13255030 | A | G | 0.016 | 0.003 | 2.80E-09 | 0.002 | 0.019 | 0.900 |
|  | rs13316611 | G | T | -0.018 | 0.003 | 1.70E-08 | 0.016 | 0.021 | 0.441 |
|  | rs139911 | C | T | 0.023 | 0.003 | 2.30E-17 | -0.030 | 0.018 | 0.106 |
|  | rs140206235 | C | T | -0.055 | 0.010 | 2.30E-08 | -0.006 | 0.121 | 0.961 |
|  | rs1421085 | T | C | -0.028 | 0.003 | 1.50E-23 | 0.008 | 0.018 | 0.661 |
|  | rs149611468 | T | C | 0.078 | 0.013 | 8.50E-10 | 0.083 | 0.062 | 0.179 |
|  | rs17161045 | T | C | 0.021 | 0.003 | 7.20E-14 | -0.004 | 0.020 | 0.832 |
|  | rs17267683 | C | G | 0.020 | 0.003 | 2.70E-10 | -0.005 | 0.021 | 0.830 |
|  | rs17448682 | C | T | -0.022 | 0.003 | 2.20E-12 | -0.022 | 0.021 | 0.310 |
|  | rs17575798 | G | A | 0.023 | 0.003 | 4.20E-11 | 0.005 | 0.023 | 0.831 |
|  | rs17604349 | G | A | 0.026 | 0.004 | 1.70E-13 | -0.005 | 0.021 | 0.803 |
|  | rs1800828 | C | G | 0.018 | 0.003 | 1.70E-08 | 0.005 | 0.022 | 0.808 |
|  | rs1947198 | C | T | -0.024 | 0.004 | 1.80E-09 | -0.002 | 0.024 | 0.945 |
|  | rs1952923 | G | A | 0.017 | 0.003 | 3.60E-08 | -0.019 | 0.021 | 0.378 |
|  | rs197273 | A | G | 0.016 | 0.003 | 1.30E-08 | 0.010 | 0.018 | 0.582 |
|  | rs202157 | C | T | 0.026 | 0.003 | 4.10E-18 | 0.013 | 0.022 | 0.551 |
|  | rs2072727 | T | C | 0.016 | 0.003 | 8.50E-10 | -0.024 | 0.018 | 0.186 |
|  | rs2239626 | T | C | -0.020 | 0.003 | 2.30E-12 | -0.053 | 0.022 | 0.013 |
|  | rs226090 | C | T | 0.021 | 0.004 | 4.10E-09 | -0.011 | 0.024 | 0.637 |
|  | rs2311837 | T | C | -0.015 | 0.003 | 3.60E-08 | -0.020 | 0.018 | 0.265 |
|  | rs231398 | G | A | 0.021 | 0.004 | 8.40E-09 | 0.019 | 0.023 | 0.414 |
|  | rs2422413 | T | C | -0.016 | 0.003 | 3.60E-09 | -0.001 | 0.019 | 0.959 |
|  | rs2518022 | T | C | 0.040 | 0.005 | 4.80E-17 | 0.033 | 0.038 | 0.388 |
|  | rs2653349 | A | G | 0.039 | 0.003 | 5.20E-32 | 0.008 | 0.023 | 0.737 |
|  | rs2712056 | C | T | -0.022 | 0.003 | 5.10E-11 | 0.000 | 0.026 | 0.987 |
|  | rs28380327 | A | T | 0.021 | 0.003 | 2.90E-13 | -0.020 | 0.019 | 0.312 |
|  | rs28458909 | C | T | 0.043 | 0.004 | 2.60E-26 | -0.001 | 0.023 | 0.976 |
|  | rs28634184 | C | T | 0.018 | 0.003 | 2.80E-08 | 0.038 | 0.022 | 0.085 |
|  | rs28647842 | G | C | 0.016 | 0.003 | 2.00E-09 | -0.009 | 0.018 | 0.621 |
|  | rs286805 | A | T | 0.014 | 0.003 | 4.50E-08 | 0.012 | 0.018 | 0.515 |
|  | rs2881955 | C | T | -0.016 | 0.003 | 4.30E-08 | -0.003 | 0.021 | 0.892 |
|  | rs2949923 | A | G | -0.016 | 0.003 | 2.10E-09 | 0.008 | 0.018 | 0.661 |
|  | rs2968511 | C | G | -0.017 | 0.003 | 4.90E-09 | 0.004 | 0.022 | 0.852 |
|  | rs2971970 | T | G | 0.019 | 0.003 | 6.00E-09 | -0.012 | 0.025 | 0.618 |
|  | rs308521 | T | C | 0.020 | 0.003 | 3.70E-13 | 0.042 | 0.019 | 0.031 |
|  | rs3100052 | A | G | 0.017 | 0.003 | 6.90E-10 | 0.031 | 0.018 | 0.092 |
|  | rs3168135 | G | A | 0.021 | 0.003 | 2.90E-11 | 0.002 | 0.020 | 0.933 |
|  | rs34239319 | G | T | -0.028 | 0.004 | 3.90E-10 | -0.033 | 0.034 | 0.322 |
|  | rs34340407 | T | C | -0.018 | 0.003 | 4.30E-09 | 0.006 | 0.021 | 0.787 |
|  | rs34619169 | G | A | -0.017 | 0.003 | 3.80E-09 | -0.018 | 0.021 | 0.372 |
|  | rs35524253 | G | A | -0.016 | 0.003 | 5.80E-09 | 0.019 | 0.019 | 0.321 |
|  | rs359250 | G | T | -0.017 | 0.003 | 9.20E-10 | 0.030 | 0.019 | 0.114 |
|  | rs3760185 | C | T | 0.024 | 0.003 | 2.60E-14 | -0.048 | 0.023 | 0.038 |
|  | rs3808477 | C | T | -0.022 | 0.003 | 2.80E-13 | -0.022 | 0.020 | 0.266 |
|  | rs3808964 | G | T | -0.016 | 0.003 | 2.70E-08 | -0.001 | 0.018 | 0.935 |
|  | rs3816454 | T | G | 0.020 | 0.003 | 4.90E-13 | 0.016 | 0.019 | 0.377 |
|  | rs3850174 | T | A | 0.019 | 0.003 | 6.80E-10 | -0.014 | 0.023 | 0.538 |
|  | rs4141920 | G | A | 0.016 | 0.003 | 4.60E-09 | -0.026 | 0.018 | 0.145 |
|  | rs4237555 | C | T | -0.016 | 0.003 | 6.00E-10 | 0.004 | 0.018 | 0.844 |
|  | rs4239386 | T | A | 0.022 | 0.003 | 1.00E-14 | 0.004 | 0.018 | 0.836 |
|  | rs4246036 | C | T | 0.024 | 0.003 | 2.40E-15 | -0.032 | 0.020 | 0.108 |
|  | rs4321976 | T | C | 0.023 | 0.003 | 3.10E-13 | 0.028 | 0.025 | 0.264 |
|  | rs4339281 | A | G | 0.022 | 0.004 | 3.20E-08 | -0.026 | 0.026 | 0.327 |
|  | rs4714475 | G | T | 0.019 | 0.003 | 1.60E-09 | -0.004 | 0.020 | 0.853 |
|  | rs4729854 | T | A | 0.029 | 0.003 | 3.30E-25 | 0.024 | 0.018 | 0.184 |
|  | rs4784655 | G | C | 0.021 | 0.003 | 1.50E-13 | 0.012 | 0.018 | 0.521 |
|  | rs4822107 | G | A | -0.016 | 0.003 | 9.60E-09 | 0.027 | 0.018 | 0.138 |
|  | rs4838161 | T | G | 0.016 | 0.003 | 2.20E-09 | 0.016 | 0.018 | 0.370 |
|  | rs4936291 | A | G | -0.018 | 0.003 | 3.80E-10 | -0.005 | 0.018 | 0.790 |
|  | rs4948547 | C | A | 0.019 | 0.003 | 1.20E-09 | -0.002 | 0.021 | 0.936 |
|  | rs4949980 | A | G | -0.032 | 0.005 | 2.70E-09 | -0.024 | 0.025 | 0.340 |
|  | rs509476 | T | C | 0.114 | 0.008 | 5.70E-47 | -0.061 | 0.071 | 0.390 |
|  | rs521977 | T | G | 0.016 | 0.003 | 2.10E-08 | 0.005 | 0.021 | 0.826 |
|  | rs56076457 | C | T | -0.016 | 0.003 | 1.30E-09 | -0.004 | 0.019 | 0.818 |
|  | rs56947091 | C | T | -0.019 | 0.003 | 6.00E-13 | -0.011 | 0.018 | 0.563 |
|  | rs57435966 | C | T | 0.055 | 0.005 | 2.60E-30 | 0.007 | 0.026 | 0.799 |
|  | rs59986227 | C | G | -0.018 | 0.003 | 8.30E-09 | -0.012 | 0.021 | 0.573 |
|  | rs60616179 | A | G | 0.038 | 0.006 | 1.10E-10 | 0.023 | 0.030 | 0.452 |
|  | rs6131942 | A | G | -0.018 | 0.003 | 9.40E-11 | -0.008 | 0.019 | 0.658 |
|  | rs61773390 | G | T | -0.034 | 0.003 | 1.20E-23 | -0.025 | 0.021 | 0.245 |
|  | rs62082401 | C | G | -0.026 | 0.003 | 1.60E-14 | 0.010 | 0.022 | 0.652 |
|  | rs62405438 | T | A | -0.020 | 0.004 | 6.30E-09 | 0.007 | 0.022 | 0.752 |
|  | rs62553781 | C | T | 0.050 | 0.007 | 1.20E-11 | -0.066 | 0.083 | 0.426 |
|  | rs6442446 | A | G | 0.017 | 0.003 | 3.10E-09 | 0.031 | 0.020 | 0.122 |
|  | rs6477309 | C | T | -0.017 | 0.003 | 8.00E-10 | 0.013 | 0.020 | 0.524 |
|  | rs6658041 | G | A | -0.015 | 0.003 | 1.70E-08 | -0.001 | 0.019 | 0.938 |
|  | rs6967481 | C | T | -0.021 | 0.003 | 6.90E-15 | -0.013 | 0.019 | 0.487 |
|  | rs698015 | C | T | -0.016 | 0.003 | 8.80E-09 | 0.014 | 0.019 | 0.457 |
|  | rs7148842 | C | T | 0.015 | 0.003 | 1.80E-08 | 0.019 | 0.018 | 0.294 |
|  | rs72632979 | A | G | 0.022 | 0.004 | 1.20E-09 | 0.011 | 0.035 | 0.755 |
|  | rs72720396 | A | G | -0.027 | 0.003 | 8.40E-18 | -0.026 | 0.024 | 0.280 |
|  | rs72829706 | A | G | 0.041 | 0.007 | 5.10E-09 | 0.023 | 0.039 | 0.558 |
|  | rs7304278 | A | G | -0.020 | 0.003 | 2.20E-11 | 0.023 | 0.021 | 0.270 |
|  | rs74357745 | A | G | 0.027 | 0.004 | 8.70E-11 | 0.056 | 0.028 | 0.045 |
|  | rs75120545 | C | T | -0.061 | 0.008 | 1.10E-13 | 0.036 | 0.044 | 0.405 |
|  | rs7547493 | A | G | -0.038 | 0.004 | 3.90E-27 | -0.028 | 0.021 | 0.176 |
|  | rs7602425 | C | T | -0.031 | 0.005 | 8.50E-10 | 0.004 | 0.046 | 0.935 |
|  | rs769066 | T | C | -0.022 | 0.003 | 3.20E-10 | 0.015 | 0.025 | 0.554 |
|  | rs7691121 | C | G | 0.022 | 0.003 | 5.60E-12 | 0.005 | 0.021 | 0.808 |
|  | rs7701529 | A | T | -0.019 | 0.003 | 3.60E-09 | -0.019 | 0.024 | 0.421 |
|  | rs7735794 | G | A | -0.020 | 0.003 | 4.70E-09 | 0.001 | 0.021 | 0.964 |
|  | rs77384811 | G | C | 0.025 | 0.004 | 2.80E-11 | 0.011 | 0.021 | 0.614 |
|  | rs786406 | A | G | -0.024 | 0.003 | 1.90E-16 | -0.025 | 0.019 | 0.183 |
|  | rs7959983 | T | C | -0.020 | 0.003 | 9.50E-13 | -0.021 | 0.019 | 0.264 |
|  | rs7977610 | A | T | 0.016 | 0.003 | 2.10E-08 | -0.015 | 0.020 | 0.457 |
|  | rs80097534 | G | T | 0.028 | 0.005 | 9.30E-10 | -0.006 | 0.027 | 0.819 |
|  | rs812925 | C | G | -0.021 | 0.003 | 6.90E-13 | 0.016 | 0.018 | 0.381 |
|  | rs827749 | T | C | -0.024 | 0.003 | 1.00E-16 | -0.028 | 0.020 | 0.165 |
|  | rs885255 | T | C | 0.017 | 0.003 | 4.40E-09 | 0.003 | 0.019 | 0.875 |
|  | rs9348050 | T | C | 0.017 | 0.003 | 7.50E-10 | -0.027 | 0.018 | 0.146 |
|  | rs9364767 | T | G | 0.016 | 0.003 | 5.40E-09 | -0.009 | 0.019 | 0.645 |
|  | rs9395520 | C | T | -0.023 | 0.003 | 2.10E-15 | -0.009 | 0.019 | 0.634 |
|  | rs9521184 | T | C | 0.015 | 0.003 | 3.00E-08 | 0.013 | 0.018 | 0.489 |
|  | rs9573971 | A | G | 0.073 | 0.007 | 3.60E-22 | 0.040 | 0.050 | 0.429 |
|  | rs957501 | T | A | 0.015 | 0.003 | 3.00E-08 | 0.007 | 0.020 | 0.726 |
|  | rs9597250 | C | A | 0.021 | 0.003 | 6.90E-10 | -0.021 | 0.024 | 0.389 |
|  | rs9636202 | G | A | 0.018 | 0.003 | 7.50E-10 | -0.010 | 0.022 | 0.644 |
|  | rs975025 | C | T | 0.032 | 0.005 | 1.30E-10 | -0.016 | 0.031 | 0.609 |
|  | rs9817740 | A | G | 0.018 | 0.003 | 2.00E-08 | 0.022 | 0.021 | 0.312 |
|  | rs9831488 | A | G | -0.018 | 0.003 | 8.40E-11 | 0.020 | 0.020 | 0.322 |
|  | rs9932577 | C | A | 0.017 | 0.003 | 6.80E-10 | 0.011 | 0.018 | 0.556 |
|  | rs9962650 | C | G | -0.017 | 0.003 | 1.60E-10 | -0.041 | 0.019 | 0.033 |
|  | rs9964420 | C | A | 0.028 | 0.003 | 8.60E-22 | 0.003 | 0.020 | 0.896 |
| Sleep duration | rs10173260 | T | C | -0.013 | 0.002 | 2.90E-08 | -0.020 | 0.018 | 0.279 |
|  | rs10483350 | A | G | -0.017 | 0.003 | 1.50E-09 | 0.013 | 0.022 | 0.572 |
|  | rs10761674 | C | T | 0.012 | 0.002 | 4.20E-08 | 0.011 | 0.018 | 0.565 |
|  | rs1079727 | T | C | -0.018 | 0.003 | 5.30E-09 | -0.023 | 0.023 | 0.319 |
|  | rs10973207 | G | T | -0.020 | 0.003 | 6.00E-11 | -0.012 | 0.021 | 0.554 |
|  | rs11039544 | G | A | 0.018 | 0.003 | 1.90E-09 | -0.002 | 0.027 | 0.932 |
|  | rs112230981 | A | G | 0.032 | 0.005 | 2.20E-09 | -0.093 | 0.054 | 0.089 |
|  | rs113113059 | T | C | 0.016 | 0.003 | 8.40E-09 | -0.009 | 0.024 | 0.716 |
|  | rs11567976 | C | T | -0.013 | 0.002 | 2.10E-08 | 0.013 | 0.018 | 0.476 |
|  | rs11621908 | C | T | 0.024 | 0.004 | 5.60E-09 | -0.007 | 0.038 | 0.860 |
|  | rs11643715 | C | G | -0.014 | 0.002 | 3.20E-08 | -0.002 | 0.022 | 0.943 |
|  | rs11885663 | C | T | -0.016 | 0.003 | 8.60E-10 | 0.006 | 0.023 | 0.795 |
|  | rs12246842 | A | G | 0.013 | 0.002 | 3.90E-09 | 0.022 | 0.018 | 0.233 |
|  | rs12567114 | G | A | -0.015 | 0.003 | 4.30E-09 | -0.004 | 0.021 | 0.856 |
|  | rs12607679 | T | C | 0.020 | 0.003 | 8.30E-15 | 0.016 | 0.022 | 0.452 |
|  | rs12791153 | A | T | -0.024 | 0.004 | 1.90E-08 | -0.001 | 0.034 | 0.968 |
|  | rs13088093 | T | G | -0.016 | 0.002 | 7.00E-12 | -0.007 | 0.020 | 0.730 |
|  | rs13109404 | T | G | 0.031 | 0.004 | 1.40E-12 | -0.039 | 0.076 | 0.612 |
|  | rs147114641 | C | A | 0.016 | 0.003 | 3.10E-09 | -0.049 | 0.069 | 0.482 |
|  | rs151014368 | G | A | -0.016 | 0.003 | 9.10E-09 | -0.015 | 0.021 | 0.465 |
|  | rs1517572 | A | C | -0.015 | 0.002 | 1.50E-10 | 0.019 | 0.019 | 0.317 |
|  | rs1553132 | A | G | -0.015 | 0.003 | 2.50E-08 | -0.037 | 0.021 | 0.076 |
|  | rs17427571 | A | G | 0.014 | 0.002 | 1.30E-08 | -0.014 | 0.019 | 0.446 |
|  | rs174560 | T | C | -0.014 | 0.002 | 2.80E-08 | -0.072 | 0.019 | 0.000 |
|  | rs17732997 | C | G | 0.013 | 0.002 | 1.20E-08 | -0.002 | 0.019 | 0.929 |
|  | rs1776776 | T | C | 0.020 | 0.003 | 4.90E-09 | -0.006 | 0.023 | 0.796 |
|  | rs1939455 | G | T | 0.020 | 0.004 | 1.20E-08 | -0.056 | 0.033 | 0.090 |
|  | rs205024 | C | T | -0.014 | 0.002 | 3.90E-09 | 0.011 | 0.018 | 0.536 |
|  | rs2072727 | T | C | 0.013 | 0.002 | 7.90E-09 | -0.024 | 0.018 | 0.186 |
|  | rs2079070 | C | G | 0.018 | 0.003 | 7.50E-12 | 0.025 | 0.023 | 0.271 |
|  | rs2192528 | A | G | 0.013 | 0.002 | 2.70E-09 | 0.016 | 0.018 | 0.373 |
|  | rs2231265 | A | G | -0.015 | 0.003 | 2.70E-08 | 0.005 | 0.024 | 0.841 |
|  | rs269054 | T | A | -0.014 | 0.002 | 2.10E-09 | 0.009 | 0.019 | 0.630 |
|  | rs2696429 | G | A | 0.017 | 0.003 | 4.00E-10 | -0.058 | 0.033 | 0.078 |
|  | rs2717076 | C | T | -0.018 | 0.002 | 3.10E-15 | -0.006 | 0.019 | 0.765 |
|  | rs3027234 | C | T | 0.015 | 0.003 | 2.30E-08 | 0.009 | 0.024 | 0.713 |
|  | rs3095508 | C | A | 0.015 | 0.002 | 3.10E-11 | -0.011 | 0.018 | 0.565 |
|  | rs34354917 | C | A | 0.014 | 0.003 | 3.90E-08 | 0.004 | 0.021 | 0.830 |
|  | rs35531607 | T | C | -0.013 | 0.002 | 1.50E-08 | -0.003 | 0.018 | 0.879 |
|  | rs35662245 | T | A | -0.015 | 0.002 | 1.40E-09 | 0.017 | 0.019 | 0.373 |
|  | rs365663 | A | G | 0.015 | 0.002 | 1.00E-10 | 0.008 | 0.018 | 0.659 |
|  | rs374153 | C | T | 0.018 | 0.003 | 9.10E-09 | 0.036 | 0.022 | 0.108 |
|  | rs4592416 | A | G | -0.015 | 0.002 | 9.30E-11 | 0.001 | 0.018 | 0.949 |
|  | rs465700 | C | G | 0.023 | 0.004 | 1.40E-09 | -0.020 | 0.027 | 0.448 |
|  | rs4767550 | A | G | -0.014 | 0.002 | 6.30E-10 | -0.003 | 0.018 | 0.872 |
|  | rs4841498 | C | T | 0.014 | 0.002 | 1.20E-09 | 0.049 | 0.018 | 0.007 |
|  | rs55658675 | C | T | 0.013 | 0.002 | 2.00E-08 | -0.007 | 0.019 | 0.725 |
|  | rs56372231 | C | T | -0.017 | 0.002 | 2.20E-12 | 0.010 | 0.019 | 0.608 |
|  | rs61985058 | C | T | -0.019 | 0.003 | 1.30E-08 | -0.045 | 0.031 | 0.148 |
|  | rs62120041 | T | C | 0.026 | 0.005 | 9.60E-09 | -0.013 | 0.033 | 0.693 |
|  | rs6575005 | T | C | 0.016 | 0.003 | 4.40E-09 | -0.030 | 0.022 | 0.183 |
|  | rs7198661 | T | C | 0.013 | 0.002 | 3.90E-09 | -0.016 | 0.019 | 0.395 |
|  | rs73219758 | G | A | 0.016 | 0.002 | 5.60E-11 | -0.021 | 0.021 | 0.326 |
|  | rs7556815 | G | A | -0.041 | 0.003 | 1.30E-49 | 0.010 | 0.022 | 0.655 |
|  | rs7644809 | T | C | 0.013 | 0.002 | 1.60E-08 | 0.012 | 0.018 | 0.523 |
|  | rs7806045 | T | C | 0.015 | 0.003 | 1.40E-08 | -0.014 | 0.020 | 0.497 |
|  | rs7915425 | T | C | 0.019 | 0.003 | 2.00E-10 | 0.011 | 0.025 | 0.648 |
|  | rs8038326 | A | G | 0.016 | 0.003 | 2.80E-10 | -0.016 | 0.020 | 0.428 |
|  | rs915416 | C | G | 0.019 | 0.002 | 9.90E-15 | -0.012 | 0.020 | 0.533 |
|  | rs9345234 | A | C | -0.013 | 0.002 | 1.80E-08 | -0.016 | 0.018 | 0.390 |
|  | rs9382445 | T | C | 0.015 | 0.002 | 4.80E-10 | -0.005 | 0.019 | 0.770 |
|  | rs9937053 | G | A | 0.017 | 0.002 | 1.20E-13 | 0.008 | 0.018 | 0.646 |
| Insomnia | rs1015438 | A | A | 0.058 | 0.008 | 2.51E-14 | 0.018 | 0.021 | 0.411 |
|  | rs1031654 | C | A | 0.051 | 0.007 | 3.88E-12 | 0.004 | 0.023 | 0.847 |
|  | rs1038093 | T | C | 0.039 | 0.006 | 2.47E-10 | 0.015 | 0.019 | 0.431 |
|  | rs10756571 | T | T | 0.036 | 0.006 | 1.80E-08 | -0.021 | 0.019 | 0.269 |
|  | rs10758593 | G | A | 0.036 | 0.006 | 4.90E-09 | 0.009 | 0.018 | 0.624 |
|  | rs10761240 | G | A | 0.043 | 0.006 | 2.12E-12 | 0.009 | 0.018 | 0.635 |
|  | rs10800992 | T | T | 0.042 | 0.006 | 3.84E-12 | 0.039 | 0.018 | 0.036 |
|  | rs10898940 | A | C | 0.034 | 0.006 | 8.09E-09 | 0.004 | 0.018 | 0.814 |
|  | rs10944696 | G | A | 0.038 | 0.007 | 7.99E-09 | -0.014 | 0.019 | 0.465 |
|  | rs10947428 | C | C | 0.068 | 0.007 | 9.06E-21 | -0.005 | 0.026 | 0.861 |
|  | rs10947690 | G | G | 0.047 | 0.007 | 4.04E-12 | -0.020 | 0.022 | 0.365 |
|  | rs10955647 | T | T | 0.033 | 0.006 | 1.84E-08 | 0.017 | 0.018 | 0.338 |
|  | rs11090039 | A | A | 0.039 | 0.007 | 1.82E-09 | -0.002 | 0.019 | 0.920 |
|  | rs11119409 | C | T | 0.035 | 0.006 | 1.19E-08 | -0.018 | 0.018 | 0.330 |
|  | rs11149313 | A | G | 0.040 | 0.007 | 2.38E-09 | -0.005 | 0.019 | 0.786 |
|  | rs113851554 | T | T | 0.206 | 0.206 | 1.56E-51 | 0.018 | 0.036 | 0.610 |
|  | rs1147852 | A | A | 0.039 | 0.006 | 9.94E-10 | 0.004 | 0.020 | 0.822 |
|  | rs11588755 | G | A | 0.035 | 0.006 | 5.14E-09 | 0.012 | 0.018 | 0.512 |
|  | rs11605348 | G | A | 0.045 | 0.006 | 7.01E-13 | -0.018 | 0.019 | 0.329 |
|  | rs116466468 | T | C | 0.044 | 0.007 | 2.11E-10 | -0.017 | 0.020 | 0.393 |
|  | rs1167132 | T | T | 0.035 | 0.006 | 8.73E-09 | 0.019 | 0.019 | 0.320 |
|  | rs11756035 | C | C | 0.051 | 0.009 | 1.29E-08 | -0.010 | 0.033 | 0.760 |
|  | rs11803128 | G | G | 0.041 | 0.006 | 6.85E-11 | -0.018 | 0.019 | 0.336 |
|  | rs118166957 | T | T | 0.068 | 0.008 | 1.95E-16 | 0.001 | 0.029 | 0.978 |
|  | rs12030482 | A | A | 0.041 | 0.007 | 8.16E-09 | 0.021 | 0.023 | 0.357 |
|  | rs12251016 | T | T | 0.039 | 0.006 | 3.89E-10 | 0.011 | 0.019 | 0.578 |
|  | rs12310246 | A | A | 0.045 | 0.007 | 4.74E-11 | -0.037 | 0.024 | 0.115 |
|  | rs12520974 | C | C | 0.036 | 0.006 | 1.69E-09 | 0.019 | 0.018 | 0.287 |
|  | rs12605642 | T | T | 0.035 | 0.006 | 2.13E-09 | -0.007 | 0.018 | 0.708 |
|  | rs12666306 | A | G | 0.042 | 0.006 | 2.24E-12 | -0.007 | 0.018 | 0.712 |
|  | rs1289939 | C | C | 0.041 | 0.007 | 6.00E-09 | 0.005 | 0.020 | 0.802 |
|  | rs12912299 | C | T | 0.043 | 0.006 | 4.42E-13 | 0.017 | 0.018 | 0.359 |
|  | rs12983032 | G | A | 0.043 | 0.006 | 1.07E-11 | -0.008 | 0.018 | 0.643 |
|  | rs12991815 | C | G | 0.040 | 0.006 | 3.02E-11 | 0.024 | 0.018 | 0.196 |
|  | rs13010288 | G | T | 0.060 | 0.009 | 9.26E-12 | 0.000 | 0.031 | 0.999 |
|  | rs13135092 | G | G | 0.089 | 0.011 | 2.53E-16 | -0.081 | 0.068 | 0.234 |
|  | rs13138995 | A | G | 0.034 | 0.006 | 1.97E-08 | -0.015 | 0.019 | 0.438 |
|  | rs1530938 | A | G | 0.036 | 0.006 | 8.82E-10 | 0.022 | 0.018 | 0.215 |
|  | rs1536053 | C | T | 0.038 | 0.006 | 6.04E-09 | 0.034 | 0.020 | 0.089 |
|  | rs1567084 | A | G | 0.033 | 0.006 | 2.14E-08 | 0.012 | 0.018 | 0.495 |
|  | rs1580173 | A | A | 0.033 | 0.006 | 2.28E-08 | -0.048 | 0.018 | 0.008 |
|  | rs1620977 | A | G | 0.052 | 0.007 | 2.27E-14 | -0.001 | 0.021 | 0.976 |
|  | rs16903122 | T | T | 0.055 | 0.007 | 9.04E-16 | 0.025 | 0.021 | 0.234 |
|  | rs16990210 | C | C | 0.046 | 0.008 | 1.97E-08 | 0.015 | 0.025 | 0.554 |
|  | rs17005118 | A | A | 0.042 | 0.007 | 6.13E-10 | -0.016 | 0.021 | 0.438 |
|  | rs17025198 | A | A | 0.041 | 0.007 | 2.19E-08 | -0.031 | 0.021 | 0.126 |
|  | rs17083297 | C | A | 0.044 | 0.008 | 1.60E-08 | -0.029 | 0.041 | 0.482 |
|  | rs17223714 | A | G | 0.046 | 0.007 | 2.44E-10 | -0.027 | 0.020 | 0.182 |
|  | rs1731951 | T | A | 0.035 | 0.006 | 1.36E-08 | 0.011 | 0.018 | 0.527 |
|  | rs17367725 | C | T | 0.036 | 0.006 | 9.29E-09 | 0.000 | 0.020 | 0.986 |
|  | rs17520265 | G | A | 0.091 | 0.016 | 2.87E-08 | -0.067 | 0.065 | 0.300 |
|  | rs17643634 | C | T | 0.060 | 0.008 | 1.34E-13 | -0.024 | 0.026 | 0.344 |
|  | rs176644 | T | T | 0.035 | 0.006 | 9.49E-09 | -0.009 | 0.018 | 0.603 |
|  | rs1927902 | T | C | 0.053 | 0.007 | 1.15E-14 | 0.014 | 0.020 | 0.484 |
|  | rs2030672 | C | C | 0.034 | 0.006 | 1.10E-08 | 0.009 | 0.018 | 0.625 |
|  | rs2089358 | C | T | 0.041 | 0.007 | 2.75E-10 | 0.004 | 0.020 | 0.826 |
|  | rs214934 | T | A | 0.038 | 0.006 | 3.16E-09 | 0.010 | 0.019 | 0.615 |
|  | rs2216427 | C | G | 0.035 | 0.006 | 1.60E-08 | -0.011 | 0.020 | 0.570 |
|  | rs2221119 | C | C | 0.036 | 0.006 | 2.00E-09 | -0.035 | 0.018 | 0.051 |
|  | rs224029 | C | C | 0.039 | 0.006 | 2.51E-10 | -0.028 | 0.019 | 0.135 |
|  | rs2364921 | C | C | 0.034 | 0.006 | 2.13E-08 | -0.014 | 0.018 | 0.444 |
|  | rs2388840 | G | A | 0.037 | 0.006 | 1.37E-09 | -0.050 | 0.020 | 0.010 |
|  | rs2389631 | C | C | 0.040 | 0.006 | 2.03E-10 | 0.005 | 0.018 | 0.772 |
|  | rs2431108 | C | C | 0.053 | 0.006 | 7.83E-17 | -0.037 | 0.020 | 0.065 |
|  | rs2598293 | T | T | 0.035 | 0.006 | 2.48E-09 | -0.015 | 0.018 | 0.426 |
|  | rs2838787 | G | A | 0.036 | 0.006 | 7.65E-09 | 0.026 | 0.019 | 0.157 |
|  | rs28552587 | A | G | 0.033 | 0.006 | 3.30E-08 | 0.013 | 0.019 | 0.496 |
|  | rs28582096 | G | A | 0.054 | 0.007 | 1.74E-13 | -0.023 | 0.022 | 0.304 |
|  | rs28611339 | T | T | 0.058 | 0.009 | 8.46E-11 | -0.031 | 0.031 | 0.308 |
|  | rs314281 | C | C | 0.043 | 0.006 | 6.03E-13 | 0.010 | 0.018 | 0.575 |
|  | rs3184470 | G | A | 0.038 | 0.006 | 9.73E-10 | 0.011 | 0.018 | 0.528 |
|  | rs324017 | A | C | 0.039 | 0.007 | 1.61E-09 | 0.015 | 0.019 | 0.430 |
|  | rs34490907 | C | G | 0.054 | 0.009 | 1.76E-08 | 0.018 | 0.030 | 0.556 |
|  | rs34967082 | A | A | 0.035 | 0.006 | 4.34E-09 | 0.031 | 0.019 | 0.090 |
|  | rs35110063 | A | A | 0.039 | 0.006 | 8.82E-11 | 0.041 | 0.018 | 0.025 |
|  | rs35322724 | A | A | 0.049 | 0.006 | 3.75E-16 | -0.012 | 0.018 | 0.522 |
|  | rs35539975 | A | G | 0.042 | 0.007 | 4.49E-09 | 0.032 | 0.020 | 0.104 |
|  | rs3774751 | G | T | 0.041 | 0.006 | 7.32E-12 | 0.021 | 0.018 | 0.252 |
|  | rs4502882 | C | T | 0.039 | 0.006 | 7.96E-10 | 0.013 | 0.020 | 0.498 |
|  | rs4588900 | A | A | 0.033 | 0.006 | 1.57E-08 | -0.013 | 0.018 | 0.464 |
|  | rs4592425 | T | T | 0.040 | 0.006 | 4.31E-10 | 0.002 | 0.020 | 0.908 |
|  | rs4664299 | C | C | 0.041 | 0.007 | 4.95E-09 | -0.012 | 0.024 | 0.606 |
|  | rs4702 | G | A | 0.048 | 0.006 | 6.78E-16 | 0.019 | 0.018 | 0.285 |
|  | rs4709655 | C | T | 0.054 | 0.009 | 3.09E-09 | 0.026 | 0.036 | 0.479 |
|  | rs4767645 | G | T | 0.037 | 0.006 | 6.47E-10 | -0.007 | 0.018 | 0.718 |
|  | rs4788203 | G | A | 0.035 | 0.006 | 6.32E-09 | 0.034 | 0.018 | 0.062 |
|  | rs4858708 | T | T | 0.034 | 0.006 | 1.23E-08 | -0.011 | 0.018 | 0.557 |
|  | rs492858 | C | T | 0.066 | 0.011 | 3.46E-09 | -0.008 | 0.036 | 0.820 |
|  | rs4981170 | G | G | 0.054 | 0.008 | 7.33E-13 | 0.001 | 0.025 | 0.981 |
|  | rs521484 | G | A | 0.040 | 0.007 | 1.53E-08 | 0.014 | 0.023 | 0.552 |
|  | rs524859 | G | A | 0.044 | 0.006 | 1.48E-12 | -0.033 | 0.019 | 0.091 |
|  | rs55772859 | A | A | 0.042 | 0.006 | 4.82E-11 | -0.046 | 0.019 | 0.016 |
|  | rs55972276 | A | A | 0.073 | 0.009 | 4.19E-17 | 0.024 | 0.030 | 0.425 |
|  | rs5877 | T | C | 0.036 | 0.006 | 1.23E-08 | -0.004 | 0.020 | 0.839 |
|  | rs6019663 | T | C | 0.040 | 0.007 | 6.47E-10 | 0.005 | 0.020 | 0.797 |
|  | rs60565673 | G | G | 0.043 | 0.006 | 1.59E-12 | 0.021 | 0.019 | 0.260 |
|  | rs6119267 | G | G | 0.060 | 0.006 | 2.32E-20 | 0.042 | 0.018 | 0.022 |
|  | rs61921611 | C | C | 0.044 | 0.006 | 7.84E-12 | -0.032 | 0.019 | 0.094 |
|  | rs62068188 | T | C | 0.049 | 0.008 | 1.18E-09 | 0.004 | 0.023 | 0.868 |
|  | rs62158170 | A | G | 0.066 | 0.007 | 1.20E-19 | 0.019 | 0.022 | 0.389 |
|  | rs62264767 | A | C | 0.065 | 0.008 | 1.63E-14 | -0.003 | 0.022 | 0.892 |
|  | rs62301574 | G | C | 0.042 | 0.007 | 1.37E-08 | -0.001 | 0.024 | 0.972 |
|  | rs62383308 | G | A | 0.060 | 0.011 | 3.98E-08 | -0.052 | 0.035 | 0.138 |
|  | rs62429521 | A | A | 0.051 | 0.008 | 1.78E-09 | 0.023 | 0.022 | 0.299 |
|  | rs638746 | A | G | 0.033 | 0.006 | 2.26E-08 | -0.046 | 0.019 | 0.015 |
|  | rs6465151 | T | T | 0.056 | 0.009 | 1.90E-09 | -0.059 | 0.029 | 0.044 |
|  | rs647905 | T | T | 0.033 | 0.006 | 2.87E-08 | 0.008 | 0.018 | 0.679 |
|  | rs6510033 | G | G | 0.037 | 0.007 | 4.66E-08 | 0.013 | 0.022 | 0.565 |
|  | rs6562066 | T | C | 0.039 | 0.006 | 1.38E-10 | -0.014 | 0.018 | 0.439 |
|  | rs6589988 | G | G | 0.038 | 0.006 | 4.70E-09 | -0.024 | 0.021 | 0.255 |
|  | rs6601080 | A | A | 0.035 | 0.006 | 2.21E-08 | 0.015 | 0.019 | 0.435 |
|  | rs66674044 | T | T | 0.060 | 0.009 | 2.18E-12 | -0.053 | 0.027 | 0.049 |
|  | rs6702604 | G | G | 0.037 | 0.006 | 1.30E-09 | -0.009 | 0.018 | 0.601 |
|  | rs671985 | G | A | 0.038 | 0.006 | 2.79E-10 | -0.002 | 0.018 | 0.910 |
|  | rs6734957 | G | G | 0.042 | 0.007 | 1.82E-09 | 0.001 | 0.022 | 0.978 |
|  | rs6756610 | C | G | 0.037 | 0.006 | 1.14E-09 | 0.004 | 0.019 | 0.810 |
|  | rs6808140 | T | T | 0.039 | 0.006 | 5.35E-11 | -0.012 | 0.018 | 0.494 |
|  | rs6888135 | A | A | 0.038 | 0.006 | 1.21E-10 | -0.018 | 0.018 | 0.328 |
|  | rs694786 | C | C | 0.044 | 0.006 | 1.97E-13 | 0.024 | 0.018 | 0.192 |
|  | rs6967168 | G | G | 0.044 | 0.007 | 1.39E-10 | -0.014 | 0.020 | 0.475 |
|  | rs6973090 | G | A | 0.038 | 0.007 | 4.31E-08 | -0.039 | 0.021 | 0.066 |
|  | rs699844 | A | G | 0.060 | 0.011 | 4.11E-08 | 0.032 | 0.042 | 0.445 |
|  | rs701394 | G | G | 0.036 | 0.006 | 6.83E-09 | 0.011 | 0.019 | 0.581 |
|  | rs7040224 | A | G | 0.037 | 0.006 | 4.24E-09 | 0.040 | 0.020 | 0.048 |
|  | rs7044885 | G | G | 0.041 | 0.006 | 5.67E-12 | 0.000 | 0.018 | 0.990 |
|  | rs715338 | A | A | 0.041 | 0.006 | 7.85E-12 | 0.044 | 0.019 | 0.019 |
|  | rs7214267 | G | A | 0.044 | 0.006 | 5.09E-13 | 0.004 | 0.018 | 0.825 |
|  | rs72657797 | C | T | 0.056 | 0.008 | 1.52E-12 | 0.035 | 0.028 | 0.203 |
|  | rs728017 | G | G | 0.035 | 0.006 | 9.51E-09 | 0.000 | 0.018 | 0.991 |
|  | rs72820274 | A | A | 0.034 | 0.006 | 1.28E-08 | -0.018 | 0.018 | 0.314 |
|  | rs72899452 | T | T | 0.074 | 0.012 | 1.00E-09 | 0.019 | 0.030 | 0.536 |
|  | rs7402939 | C | C | 0.036 | 0.006 | 5.19E-09 | 0.021 | 0.019 | 0.254 |
|  | rs742760 | A | T | 0.043 | 0.008 | 2.48E-08 | -0.022 | 0.024 | 0.367 |
|  | rs7475916 | G | G | 0.037 | 0.006 | 6.70E-09 | 0.006 | 0.019 | 0.738 |
|  | rs7571486 | G | A | 0.039 | 0.007 | 1.40E-08 | 0.019 | 0.021 | 0.348 |
|  | rs76145129 | G | T | 0.050 | 0.009 | 2.73E-08 | -0.028 | 0.030 | 0.340 |
|  | rs769449 | G | A | 0.046 | 0.008 | 2.13E-08 | -0.067 | 0.025 | 0.009 |
|  | rs77641763 | T | T | 0.071 | 0.009 | 6.53E-15 | -0.003 | 0.023 | 0.913 |
|  | rs79693059 | G | G | 0.073 | 0.011 | 1.61E-11 | 0.040 | 0.038 | 0.290 |
|  | rs8076183 | C | T | 0.038 | 0.006 | 2.75E-10 | -0.021 | 0.018 | 0.253 |
|  | rs8180457 | C | C | 0.056 | 0.008 | 1.12E-11 | -0.035 | 0.026 | 0.172 |
|  | rs8180817 | G | C | 0.049 | 0.006 | 1.83E-16 | -0.019 | 0.018 | 0.308 |
|  | rs823247 | C | T | 0.037 | 0.006 | 5.25E-10 | 0.042 | 0.018 | 0.022 |
|  | rs830716 | C | C | 0.045 | 0.007 | 8.68E-12 | -0.015 | 0.018 | 0.415 |
|  | rs871994 | A | A | 0.035 | 0.006 | 5.50E-09 | -0.027 | 0.019 | 0.153 |
|  | rs908668 | T | T | 0.050 | 0.007 | 1.41E-11 | 0.031 | 0.025 | 0.205 |
|  | rs910187 | G | A | 0.035 | 0.006 | 1.63E-08 | 0.046 | 0.019 | 0.016 |
|  | rs9527083 | G | A | 0.076 | 0.006 | 1.61E-32 | 0.006 | 0.019 | 0.760 |
|  | rs9889282 | C | C | 0.042 | 0.006 | 4.70E-12 | -0.014 | 0.019 | 0.469 |
|  | rs9931543 | T | C | 0.048 | 0.007 | 1.11E-12 | 0.021 | 0.021 | 0.319 |
|  | rs9964420 | A | A | 0.035 | 0.007 | 4.54E-08 | 0.003 | 0.020 | 0.896 |
| Daytime sleepiness | rs11123962 | T | G | -0.008 | 0.001 | 7.50E-15 | -0.024 | 0.018 | 0.192 |
|  | rs11942333 | G | A | -0.006 | 0.001 | 3.80E-08 | 0.008 | 0.021 | 0.692 |
|  | rs12140153 | G | T | 0.017 | 0.002 | 2.80E-20 | -0.046 | 0.034 | 0.176 |
|  | rs12153518 | A | G | 0.007 | 0.001 | 6.80E-11 | 0.006 | 0.018 | 0.721 |
|  | rs13010456 | A | G | 0.008 | 0.001 | 2.10E-13 | -0.009 | 0.019 | 0.644 |
|  | rs13097760 | A | C | -0.006 | 0.001 | 3.20E-08 | 0.029 | 0.019 | 0.133 |
|  | rs13135092 | A | G | -0.010 | 0.002 | 3.10E-08 | -0.081 | 0.068 | 0.234 |
|  | rs147114641 | C | A | 0.008 | 0.001 | 1.70E-10 | -0.049 | 0.069 | 0.482 |
|  | rs1566362 | T | C | 0.006 | 0.001 | 3.80E-09 | 0.000 | 0.018 | 0.996 |
|  | rs17131124 | C | G | -0.011 | 0.002 | 1.70E-09 | 0.020 | 0.029 | 0.496 |
|  | rs17356118 | A | G | -0.008 | 0.001 | 2.60E-10 | 0.018 | 0.025 | 0.455 |
|  | rs1846644 | T | C | -0.011 | 0.001 | 2.50E-27 | 0.000 | 0.018 | 0.982 |
|  | rs2048522 | A | T | 0.006 | 0.001 | 3.50E-08 | 0.035 | 0.018 | 0.055 |
|  | rs2787120 | A | G | 0.008 | 0.001 | 2.00E-08 | 0.006 | 0.023 | 0.791 |
|  | rs285793 | G | A | 0.007 | 0.001 | 7.90E-11 | -0.009 | 0.019 | 0.645 |
|  | rs3122170 | C | A | 0.010 | 0.001 | 5.60E-15 | 0.002 | 0.024 | 0.929 |
|  | rs4665972 | T | C | 0.007 | 0.001 | 3.90E-10 | 0.044 | 0.019 | 0.019 |
|  | rs4765939 | G | C | -0.006 | 0.001 | 2.00E-09 | 0.024 | 0.020 | 0.231 |
|  | rs501701 | A | G | 0.006 | 0.001 | 6.50E-09 | 0.001 | 0.018 | 0.956 |
|  | rs55818482 | T | C | -0.010 | 0.001 | 1.40E-14 | -0.004 | 0.024 | 0.884 |
|  | rs55960940 | T | C | 0.008 | 0.001 | 2.00E-08 | 0.024 | 0.022 | 0.279 |
|  | rs57746981 | C | T | 0.007 | 0.001 | 2.20E-10 | 0.001 | 0.019 | 0.959 |
|  | rs62055936 | T | A | 0.008 | 0.001 | 4.40E-11 | -0.061 | 0.032 | 0.062 |
|  | rs62519825 | T | C | -0.009 | 0.002 | 3.80E-09 | -0.051 | 0.026 | 0.053 |
|  | rs6741951 | G | A | 0.007 | 0.001 | 2.70E-09 | -0.013 | 0.021 | 0.549 |
|  | rs6897863 | A | C | 0.006 | 0.001 | 7.60E-10 | 0.002 | 0.018 | 0.914 |
|  | rs6923811 | T | C | 0.007 | 0.001 | 9.10E-10 | 0.009 | 0.023 | 0.696 |
|  | rs7476897 | G | A | 0.007 | 0.001 | 2.70E-11 | -0.003 | 0.022 | 0.898 |
|  | rs7598712 | G | T | 0.006 | 0.001 | 2.20E-08 | -0.031 | 0.018 | 0.090 |
|  | rs7607363 | A | G | -0.006 | 0.001 | 8.00E-09 | -0.037 | 0.018 | 0.042 |
|  | rs7837226 | A | G | -0.006 | 0.001 | 2.00E-08 | -0.010 | 0.018 | 0.583 |
|  | rs8015449 | A | G | 0.006 | 0.001 | 1.90E-09 | 0.003 | 0.018 | 0.860 |
|  | rs825127 | T | G | 0.006 | 0.001 | 9.50E-09 | 0.013 | 0.018 | 0.469 |
|  | rs843372 | C | T | 0.008 | 0.001 | 2.20E-11 | -0.022 | 0.020 | 0.272 |
|  | rs886114 | C | T | 0.006 | 0.001 | 1.90E-08 | 0.018 | 0.020 | 0.360 |
|  | rs960986 | C | T | 0.007 | 0.001 | 1.50E-11 | 0.019 | 0.018 | 0.294 |
|  | rs9712275 | C | T | -0.006 | 0.001 | 1.30E-08 | -0.027 | 0.018 | 0.146 |
| Daytime napping | rs1001817 | C | T | 0.008 | 0.001 | 1.70E-10 | -0.008 | 0.018 | 0.653 |
|  | rs10149986 | T | G | -0.011 | 0.002 | 4.40E-12 | -0.002 | 0.026 | 0.926 |
|  | rs10152428 | G | C | -0.008 | 0.001 | 1.10E-08 | -0.021 | 0.019 | 0.290 |
|  | rs10257273 | A | T | 0.011 | 0.002 | 8.90E-10 | 0.037 | 0.022 | 0.087 |
|  | rs10811438 | G | C | 0.007 | 0.001 | 3.20E-09 | 0.018 | 0.019 | 0.346 |
|  | rs10835420 | T | A | 0.009 | 0.001 | 1.70E-10 | -0.003 | 0.025 | 0.892 |
|  | rs10840017 | A | G | 0.009 | 0.001 | 2.00E-09 | -0.017 | 0.025 | 0.506 |
|  | rs10875606 | C | A | 0.007 | 0.001 | 1.30E-08 | 0.007 | 0.020 | 0.718 |
|  | rs10875622 | G | A | -0.010 | 0.001 | 1.30E-17 | -0.009 | 0.019 | 0.632 |
|  | rs11071755 | G | A | 0.007 | 0.001 | 5.20E-09 | 0.040 | 0.019 | 0.041 |
|  | rs112520848 | G | C | -0.007 | 0.001 | 1.80E-08 | -0.016 | 0.019 | 0.414 |
|  | rs11258652 | C | A | 0.010 | 0.001 | 3.70E-13 | -0.019 | 0.020 | 0.352 |
|  | rs11615756 | C | T | -0.018 | 0.001 | 1.40E-49 | -0.003 | 0.018 | 0.873 |
|  | rs11682175 | T | C | -0.007 | 0.001 | 1.10E-08 | 0.010 | 0.018 | 0.590 |
|  | rs11860072 | C | T | 0.009 | 0.001 | 2.80E-13 | -0.018 | 0.019 | 0.336 |
|  | rs12042846 | T | C | -0.009 | 0.002 | 1.90E-08 | -0.016 | 0.024 | 0.503 |
|  | rs12140153 | G | T | 0.025 | 0.002 | 2.40E-31 | -0.046 | 0.034 | 0.176 |
|  | rs12346996 | T | C | 0.008 | 0.001 | 5.00E-09 | -0.043 | 0.021 | 0.046 |
|  | rs12451365 | T | C | -0.011 | 0.002 | 1.50E-12 | 0.002 | 0.021 | 0.915 |
|  | rs12657723 | C | T | -0.008 | 0.001 | 2.00E-10 | 0.012 | 0.019 | 0.523 |
|  | rs12992648 | A | G | 0.008 | 0.001 | 2.40E-08 | 0.024 | 0.021 | 0.245 |
|  | rs13033444 | A | G | -0.010 | 0.001 | 3.60E-13 | -0.033 | 0.020 | 0.101 |
|  | rs13150944 | A | G | -0.009 | 0.001 | 6.80E-11 | 0.014 | 0.021 | 0.486 |
|  | rs13263535 | G | T | 0.007 | 0.001 | 1.60E-08 | -0.012 | 0.018 | 0.527 |
|  | rs13284688 | T | C | -0.015 | 0.001 | 1.70E-23 | 0.001 | 0.025 | 0.961 |
|  | rs140506252 | A | T | 0.023 | 0.004 | 4.30E-08 | 0.021 | 0.057 | 0.713 |
|  | rs1546977 | A | G | -0.008 | 0.001 | 1.00E-11 | -0.012 | 0.018 | 0.492 |
|  | rs1601440 | C | T | 0.009 | 0.001 | 1.90E-11 | -0.006 | 0.021 | 0.777 |
|  | rs17158413 | G | A | -0.009 | 0.001 | 4.40E-11 | 0.019 | 0.023 | 0.409 |
|  | rs17265513 | T | C | -0.009 | 0.002 | 2.00E-09 | 0.003 | 0.020 | 0.889 |
|  | rs174541 | T | C | -0.010 | 0.001 | 4.40E-15 | -0.057 | 0.018 | 0.002 |
|  | rs17502738 | T | C | 0.009 | 0.002 | 2.00E-08 | -0.001 | 0.023 | 0.968 |
|  | rs1883048 | T | C | -0.008 | 0.001 | 1.80E-10 | -0.021 | 0.018 | 0.245 |
|  | rs1931175 | C | G | -0.008 | 0.001 | 4.60E-10 | -0.022 | 0.018 | 0.225 |
|  | rs2033103 | C | T | -0.007 | 0.001 | 2.60E-09 | 0.025 | 0.018 | 0.177 |
|  | rs2099810 | A | G | 0.008 | 0.001 | 2.80E-10 | 0.015 | 0.018 | 0.404 |
|  | rs2202323 | G | C | 0.008 | 0.001 | 1.20E-09 | -0.012 | 0.019 | 0.547 |
|  | rs224111 | G | A | 0.008 | 0.001 | 1.60E-10 | 0.021 | 0.018 | 0.252 |
|  | rs2250377 | A | G | 0.013 | 0.001 | 1.40E-24 | -0.023 | 0.020 | 0.252 |
|  | rs2284015 | C | G | -0.008 | 0.001 | 4.10E-08 | -0.030 | 0.019 | 0.104 |
|  | rs2370926 | T | C | 0.008 | 0.001 | 1.70E-10 | -0.008 | 0.022 | 0.705 |
|  | rs2431108 | T | C | -0.013 | 0.001 | 7.70E-24 | -0.037 | 0.020 | 0.065 |
|  | rs253666 | G | A | -0.008 | 0.001 | 1.80E-08 | -0.007 | 0.021 | 0.731 |
|  | rs2653349 | A | G | 0.017 | 0.001 | 3.40E-29 | 0.008 | 0.023 | 0.737 |
|  | rs2699869 | A | C | 0.007 | 0.001 | 3.10E-08 | 0.001 | 0.018 | 0.972 |
|  | rs2769916 | G | A | -0.009 | 0.001 | 1.70E-11 | 0.009 | 0.019 | 0.617 |
|  | rs2786547 | C | T | 0.011 | 0.002 | 1.00E-11 | 0.008 | 0.023 | 0.727 |
|  | rs285815 | T | A | 0.008 | 0.001 | 5.80E-10 | -0.010 | 0.019 | 0.578 |
|  | rs2943023 | C | T | 0.007 | 0.001 | 7.40E-09 | -0.010 | 0.018 | 0.586 |
|  | rs295278 | G | A | 0.008 | 0.001 | 2.20E-10 | -0.012 | 0.018 | 0.498 |
|  | rs34262487 | C | A | 0.015 | 0.002 | 1.20E-09 | 0.069 | 0.045 | 0.125 |
|  | rs35011311 | G | T | 0.009 | 0.001 | 4.40E-11 | 0.007 | 0.021 | 0.737 |
|  | rs350785 | T | C | 0.013 | 0.002 | 1.50E-11 | 0.010 | 0.030 | 0.731 |
|  | rs351776 | A | C | -0.008 | 0.001 | 8.40E-10 | -0.021 | 0.018 | 0.258 |
|  | rs35851551 | A | G | 0.011 | 0.002 | 3.50E-08 | 0.001 | 0.039 | 0.971 |
|  | rs35851551 | A | G | 0.011 | 0.002 | 3.50E-08 | 1.965 | 0.917 | 0.032 |
|  | rs3799380 | T | C | 0.009 | 0.002 | 1.30E-08 | 0.048 | 0.026 | 0.071 |
|  | rs3810484 | A | G | 0.007 | 0.001 | 2.20E-08 | -0.005 | 0.019 | 0.804 |
|  | rs385199 | A | C | 0.021 | 0.001 | 7.50E-47 | -0.058 | 0.032 | 0.072 |
|  | rs3935190 | G | A | -0.008 | 0.001 | 5.40E-11 | 0.013 | 0.018 | 0.481 |
|  | rs4604518 | G | A | 0.007 | 0.001 | 1.40E-08 | 0.013 | 0.018 | 0.495 |
|  | rs4653052 | C | A | -0.007 | 0.001 | 3.40E-08 | 0.023 | 0.019 | 0.227 |
|  | rs467897 | G | A | 0.010 | 0.001 | 2.60E-13 | -0.026 | 0.019 | 0.178 |
|  | rs4692709 | C | T | 0.007 | 0.001 | 2.40E-09 | 0.018 | 0.019 | 0.331 |
|  | rs60222088 | C | A | 0.011 | 0.002 | 6.40E-11 | -0.019 | 0.022 | 0.396 |
|  | rs60920123 | G | A | 0.008 | 0.001 | 4.50E-10 | -0.011 | 0.019 | 0.553 |
|  | rs614987 | A | C | -0.011 | 0.001 | 7.50E-19 | 0.032 | 0.019 | 0.096 |
|  | rs62189006 | A | G | 0.012 | 0.002 | 8.30E-09 | 0.012 | 0.028 | 0.674 |
|  | rs6452787 | A | G | 0.008 | 0.001 | 2.00E-10 | 0.014 | 0.018 | 0.439 |
|  | rs6919087 | T | G | 0.011 | 0.001 | 1.00E-16 | 0.002 | 0.020 | 0.927 |
|  | rs73817091 | C | T | -0.017 | 0.003 | 3.80E-08 | 0.052 | 0.059 | 0.376 |
|  | rs7422655 | C | T | 0.008 | 0.001 | 1.10E-08 | 0.021 | 0.020 | 0.298 |
|  | rs7423968 | A | G | 0.008 | 0.001 | 4.80E-10 | -0.003 | 0.018 | 0.870 |
|  | rs75022160 | C | T | 0.010 | 0.002 | 1.70E-08 | 0.009 | 0.031 | 0.769 |
|  | rs76257331 | G | A | -0.009 | 0.002 | 1.20E-08 | 0.018 | 0.025 | 0.482 |
|  | rs7697461 | A | T | -0.008 | 0.001 | 6.00E-09 | 0.004 | 0.020 | 0.855 |
|  | rs77154532 | A | G | 0.008 | 0.001 | 1.40E-09 | 0.010 | 0.019 | 0.589 |
|  | rs7814873 | C | T | 0.007 | 0.001 | 1.50E-08 | -0.010 | 0.019 | 0.606 |
|  | rs785145 | T | G | -0.007 | 0.001 | 1.00E-08 | 0.002 | 0.018 | 0.911 |
|  | rs7932966 | A | T | 0.009 | 0.001 | 1.10E-09 | -0.010 | 0.022 | 0.657 |
|  | rs80163246 | T | C | -0.012 | 0.002 | 1.40E-10 | -0.010 | 0.027 | 0.696 |
|  | rs908442 | A | T | 0.010 | 0.001 | 1.40E-16 | -0.008 | 0.019 | 0.681 |
|  | rs910187 | G | A | 0.007 | 0.001 | 4.90E-09 | 0.046 | 0.019 | 0.016 |
|  | rs9287862 | C | T | 0.012 | 0.002 | 2.00E-08 | 0.000 | 0.030 | 0.993 |
|  | rs9309116 | C | T | -0.007 | 0.001 | 3.90E-09 | -0.038 | 0.018 | 0.038 |
|  | rs9389556 | C | G | -0.008 | 0.001 | 9.70E-10 | 0.043 | 0.022 | 0.049 |
|  | rs9460110 | T | C | -0.007 | 0.001 | 8.40E-09 | -0.002 | 0.020 | 0.905 |
|  | rs9475168 | T | G | 0.009 | 0.001 | 1.90E-09 | 0.010 | 0.021 | 0.625 |
|  | rs962247 | G | A | 0.008 | 0.001 | 5.70E-11 | 0.004 | 0.018 | 0.833 |
|  | rs971415 | A | G | 0.011 | 0.002 | 1.20E-09 | 0.019 | 0.023 | 0.412 |
|  | rs9939355 | C | T | 0.007 | 0.001 | 4.50E-09 | 0.017 | 0.019 | 0.377 |

**Supplementary Table 7.** Manually detected potential pleiotropy in the PhenoScanner database.

| **SNP** | **effect_allele** | **other_allele** | **beta** | **se** | **pval** | **Potential pleiotropic trait** |
| --- | --- | --- | --- | --- | --- | --- |
| Chronotype to CRC |  |  |  |  |  |  |
| rs72720396 | A | G | -0.0271261 | 0.00318521 | 8.40E-18 | Alcohol usually taken with meals/Average weekly beer plus cider intake |
| rs11712056 | T | C | 0.0200896 | 0.00270384 | 1.60E-13 | Ulcerative colitis/  Average weekly beer plus cider intake |
| rs17161045 | T | C | 0.0206395 | 0.0028029 | 7.20E-14 | Pack years adult smoking as proportion of life span exposed to smoking/Pack years of smoking preview only |
| rs1421085 | T | C | -0.0275621 | 0.00274075 | 1.50E-23 | Average weekly beer plus cider intake |
| rs12140153 | G | T | 0.0340109 | 0.00468803 | 1.60E-13 | Body mass index |
| rs1064213 | G | A | -0.019141 | 0.00268602 | 1.30E-12 | Body mass index |
| rs11712056 | T | C | 0.0200896 | 0.00270384 | 1.60E-13 | Body mass index |
| rs4241964 | T | G | -0.020735 | 0.00270449 | 1.10E-14 | Body mass index |
| rs9395520 | C | T | -0.0231272 | 0.00291912 | 2.10E-15 | Body mass index |
| rs62405438 | T | A | -0.0201979 | 0.00350204 | 6.30E-09 | Body mass index |
| rs4321976 | T | C | 0.0232959 | 0.00324034 | 3.10E-13 | Body mass index |
| rs3808477 | C | T | -0.0215613 | 0.00299116 | 2.80E-13 | Body mass index |
| Sleep duration to CRC |  |  |  |  |  |  |
| rs9937053 | G | A | 0.0169348 | 0.00229041 | 1.20E-13 | Average weekly beer plus cider intake |
| rs2192528 | A | G | 0.0133687 | 0.0022692 | 2.70E-09 | Body mass index |
| rs13109404 | T | G | 0.0312035 | 0.00440829 | 1.40E-12 | Body mass index |
| rs34731055 | C | T | -0.0194603 | 0.00294778 | 3.70E-11 | Body mass index |
| rs4841498 | C | T | 0.0139954 | 0.00226922 | 1.20E-09 | Body mass index |
| rs13088093 | T | G | -0.0162722 | 0.00240235 | 7.00E-12 | Body mass index |
| rs73219758 | G | A | 0.0164012 | 0.00249526 | 5.60E-11 | Body mass index |
| rs12246842 | A | G | 0.0133949 | 0.00227425 | 3.90E-09 | Body mass index |
| rs4592416 | A | G | -0.0146828 | 0.0022701 | 9.30E-11 | Body mass index |
| rs9937053 | G | A | 0.0169348 | 0.00229041 | 1.20E-13 | Body mass index/Body mass index in smokers/  Obesity body mass index |
| Daytimesleepiness of CRC |  |  |  |  |  |  |
| rs2787120 | A | G | 0.00778398 | 0.00137756 | 2.00E-08 | Body mass index |
| rs12140153 | G | T | 0.01658 | 0.00179639 | 2.80E-20 | Body mass index |
| rs57746981 | C | T | 0.00680998 | 0.00107584 | 2.20E-10 | Body mass index |
| rs6741951 | G | A | 0.00681617 | 0.00114107 | 2.70E-09 | Body mass index |
| rs9712275 | C | T | -0.00588405 | 0.0010319 | 1.30E-08 | Body mass index |
| rs7607363 | A | G | -0.0060244 | 0.00103841 | 8.00E-09 | Body mass index |
| rs960986 | C | T | 0.00716034 | 0.00106799 | 1.50E-11 | Body mass index |
| rs13135092 | A | G | -0.0103303 | 0.0018774 | 3.10E-08 | Body mass index |
| rs4665972 | T | C | 0.0066245 | 0.00105853 | 3.90E-10 | Alcohol intake frequency/Alcohol intake versus 10 years previously |
| rs11123962 | T | G | -0.00804433 | 0.00103439 | 7.50E-15 | Alcohol intake versus 10 years previously/Ever smoked/Past tobacco smoking/  Smoking status: previous |
| rs13135092 | A | G | -0.0103303 | 0.0018774 | 3.10E-08 | Crohns disease/Alcohol intake frequency |
| rs62055936 | T | A | 0.00819979 | 0.00124114 | 4.40E-11 | Alcohol intake frequency |
| Daytimenapping of CRC |  |  |  |  |  |  |
| rs2786547 | C | T | 0.0107673 | 0.00158775 | 1.00E-11 | Body mass index |
| rs12140153 | G | T | 0.0247147 | 0.00211564 | 2.40E-31 | Body mass index |
| rs1931175 | C | G | -0.00776325 | 0.00124756 | 4.60E-10 | Body mass index |
| rs2250377 | A | G | 0.0131561 | 0.00127975 | 1.40E-24 | Body mass index |
| rs76824303 | A | C | 0.0117977 | 0.00207749 | 1.70E-08 | Body mass index |
| rs1001817 | C | T | 0.00775426 | 0.00121418 | 1.70E-10 | Body mass index |
| rs10257273 | A | T | 0.0107366 | 0.00170413 | 8.90E-10 | Body mass index |
| rs378421 | G | A | -0.00697888 | 0.0012384 | 2.40E-08 | Body mass index/Crohns disease/  Alcohol intake frequency |
| rs6452787 | A | G | 0.00772931 | 0.0012169 | 2.00E-10 | Alcohol intake frequency |
| rs224111 | G | A | 0.00798411 | 0.00124912 | 1.60E-10 | Crohns disease |
| Insomnia of CRC |  |  |  |  |  |  |
| rs13135092 | G | A | 0.089 | 0.011 | 2.53E-16 | Crohns disease/Alcohol intake frequency/Body mass index |
| rs1620977 | A | G | 0.052 | 0.007 | 2.27E-14 | Body mass index |
| rs694786 | C | T | 0.044 | 0.006 | 1.97E-13 | Body mass index |
| rs11605348 | G | A | 0.045 | 0.006 | 7.01E-13 | Body mass index/Alcohol intake frequency |
| rs524859 | G | A | 0.044 | 0.006 | 1.48E-12 | Body mass index |
| rs66674044 | T | A | 0.06 | 0.009 | 2.18E-12 | Body mass index |
| rs1031654 | C | A | 0.051 | 0.007 | 3.88E-12 | Body mass index |
| rs3774751 | G | T | 0.041 | 0.006 | 7.32E-12 | Body mass index |
| rs1064939 | A | T | 0.13 | 0.02 | 2.16E-10 | Body mass index |
| rs12251016 | T | A | 0.039 | 0.006 | 3.89E-10 | Body mass index |
| rs4502882 | C | T | 0.039 | 0.006 | 7.96E-10 | Body mass index |
| rs62264767 | A | C | 0.065 | 0.008 | 1.63E-14 | Ever smoked/Past tobacco smoking/Smoking status: previous |
| rs224029 | C | T | 0.039 | 0.006 | 2.51E-10 | Crohns disease |
| rs1536053 | C | T | 0.038 | 0.006 | 6.04E-09 | Body mass index |
| rs4788203 | G | A | 0.035 | 0.006 | 6.32E-09 | Body mass index |
| rs11119409 | C | T | 0.035 | 0.006 | 1.19E-08 | Body mass index |
| rs72820274 | A | G | 0.034 | 0.006 | 1.28E-08 | Body mass index/Alcohol intake versus 10 years previously |
| rs769449 | G | A | 0.046 | 0.008 | 2.13E-08 | Body mass index |
| rs742760 | A | T | 0.043 | 0.008 | 2.48E-08 | Body mass index |
| rs176644 | T | G | 0.035 | 0.006 | 9.49E-09 | Ever smoked |
| rs5877 | T | C | 0.036 | 0.006 | 1.23E-08 | Alcohol usually taken with meals |

**Supplementary Table 8.** Heterogeneity test and pleiotropy test after removing SNPs with potential pleiotropy.

| **Exposure/Outcome** | **MR-IVW** | | | **MR-Egger** | | | **MR-Egger intercept** | | | **MR-PRESSO** |
| --- | --- | --- | --- | --- | --- | --- | --- | --- | --- | --- |
|  | **Q** | **Q_df** | **Q_pval** | **Q** | **Q_df** | **Q_pval** | **Intercept** | **SE** | ***P*** val | **Global test Pval** |
| Chronotype/CRC | 112.89 | 112 | 0.46 | 112.76 | 111 | 0.44 | 2.09E-03 | 5.69E-03 | 0.71 |  |
| Sleep duration/CRC | 57.70 | 49 | 0.18 | 55.81 | 48 | 0.20 | -0.014 | 0.011 | 0.21 |  |
| Insomnia/CRC | 133.83 | 112 | 0.08 | 132.37 | 111 | 0.08 | -7.99E-03 | 7.22E-03 | 0.27 |  |
| Daytime sleepiness/CRC | 26.89 | 32 | 0.72 | 26.37 | 31 | 0.70 | -0.11 | 0.015 | 0.48 |  |
| Daytime napping/CRC | 81.31 | 72 | 0.21 | 81.23 | 71 | 0.19 | -2.46E-03 | 9.51E-03 | 0.80 |  |

*No SNPs with potential pleiotropy were identifie

**Supplementary Figure S1 Forest plot (A), sensitivity analysis (B), scatter plot (C) , funnel plot (D) and density (E) of the causal effect of Sleep duration on CRC risk。**

1. **(B)**

**
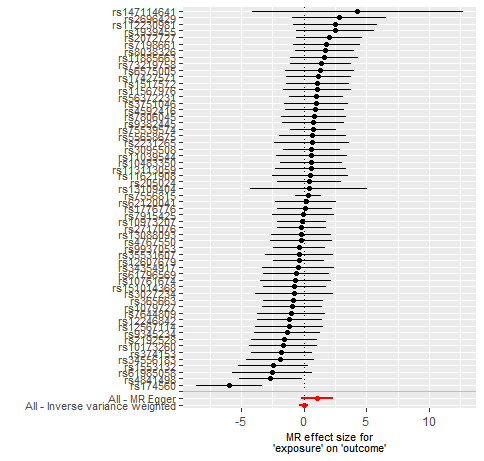

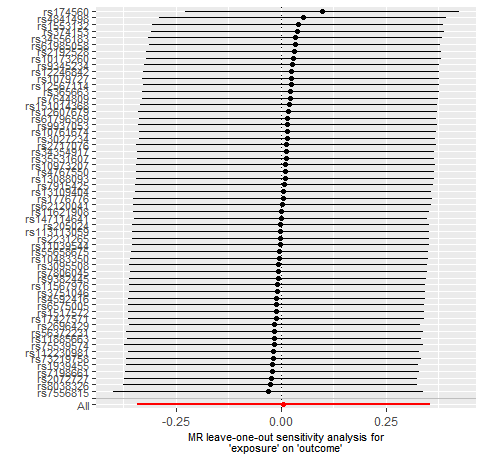
**

**(C) (D)**

**
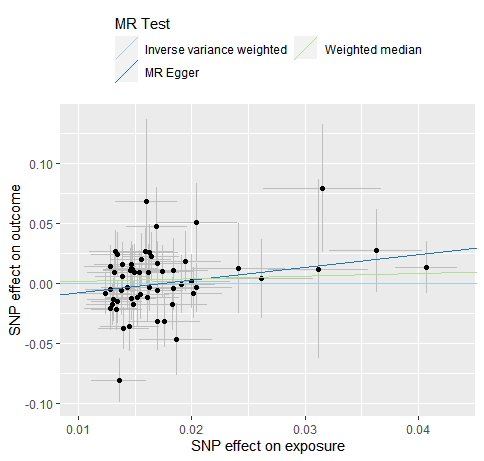

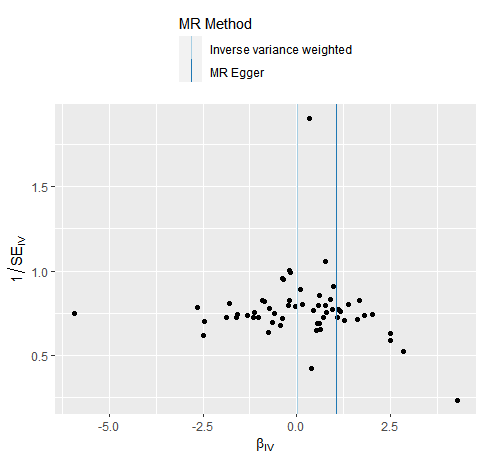
**

**(E)**

**
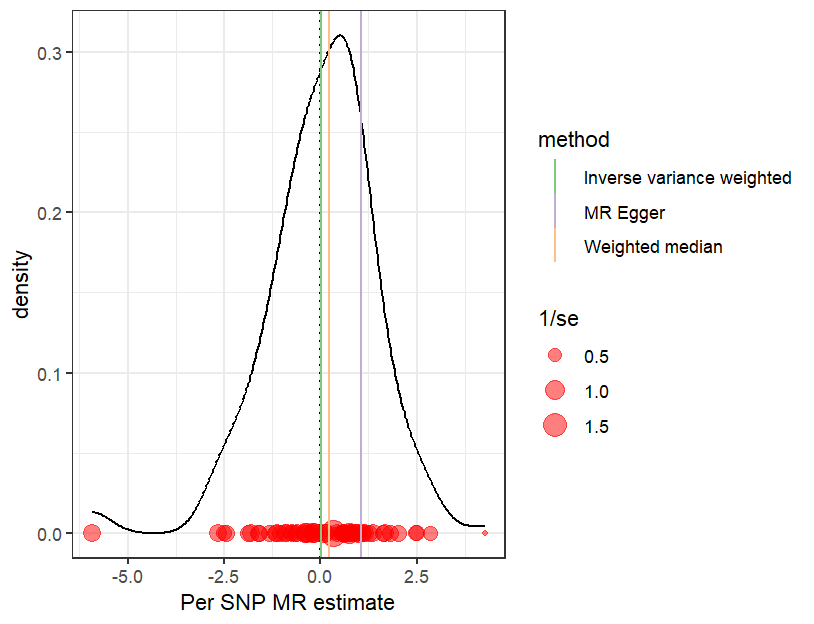
**

**Supplementary Figure S2 Forest plot (A), sensitivity analysis (B), scatter plot (C) , funnel plot (D) and density (E) of the causal effect of Insomnia on CRC risk。**

**(A) (B)**

**
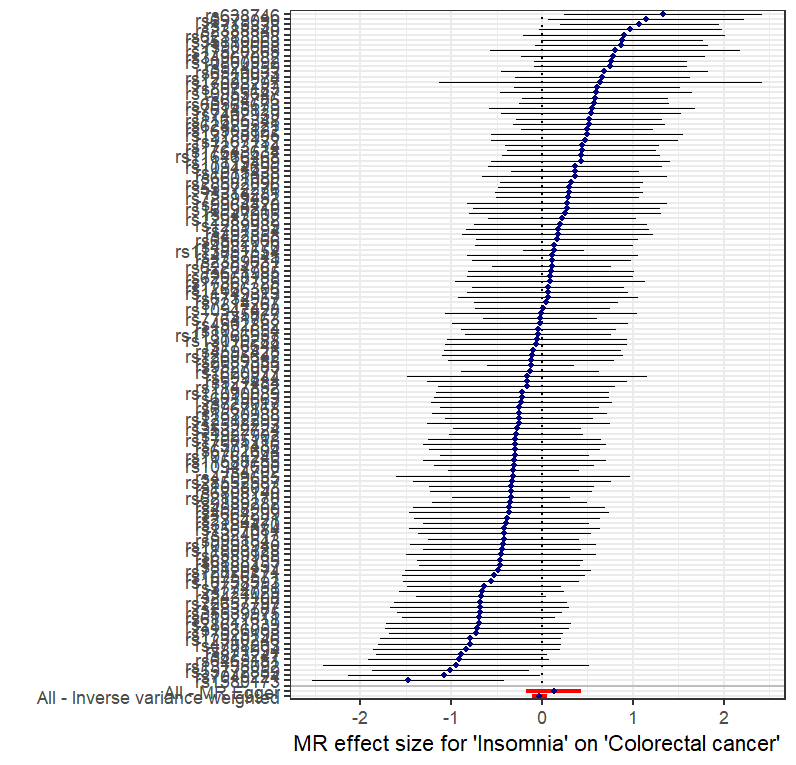

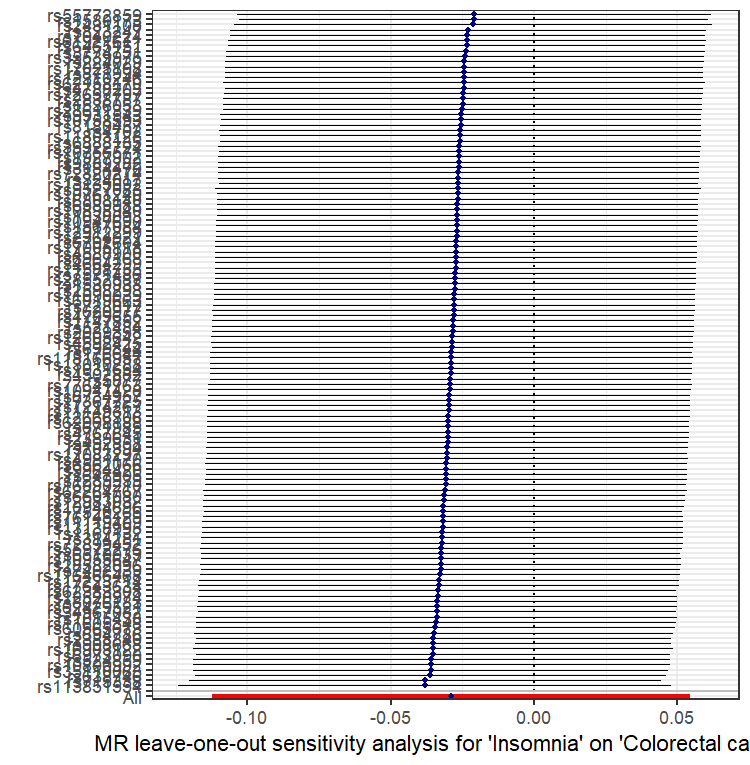
**

**(C)**

**
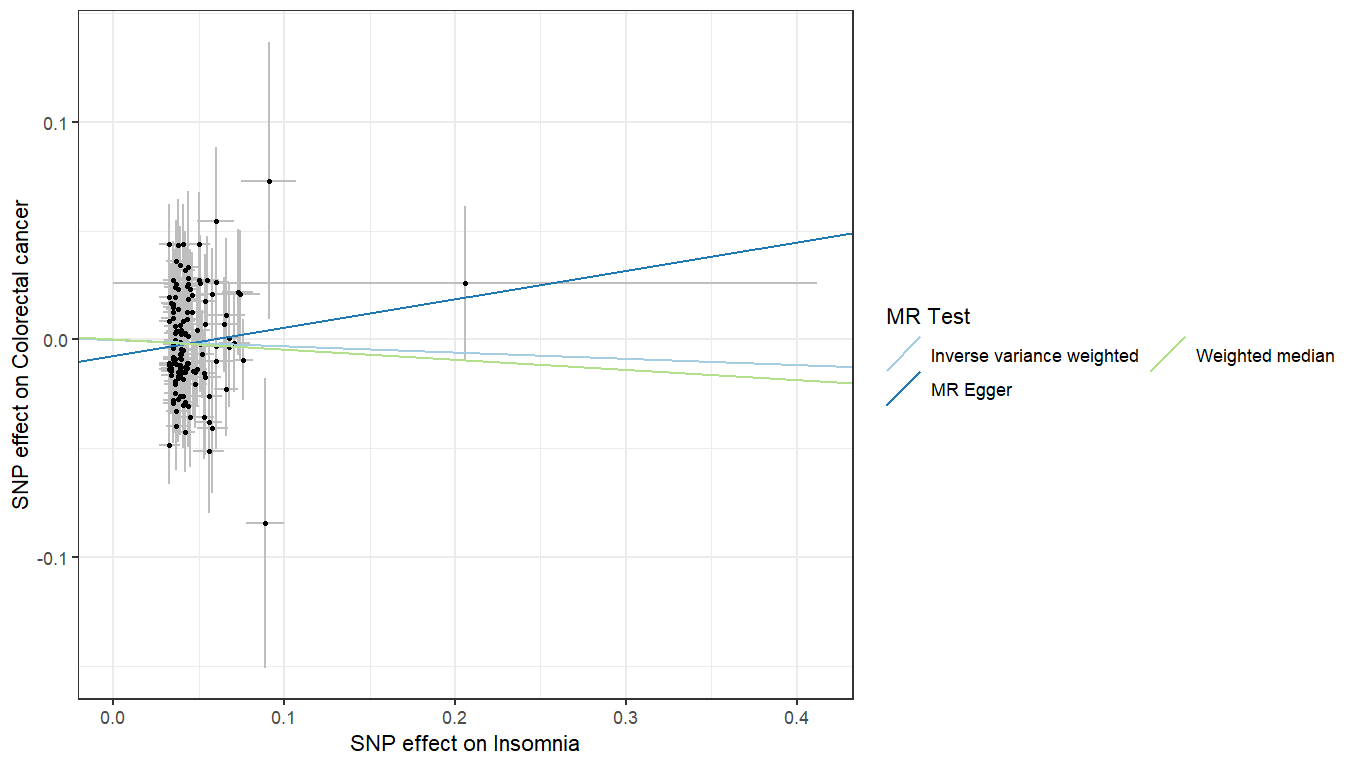
**

**(D)**

**
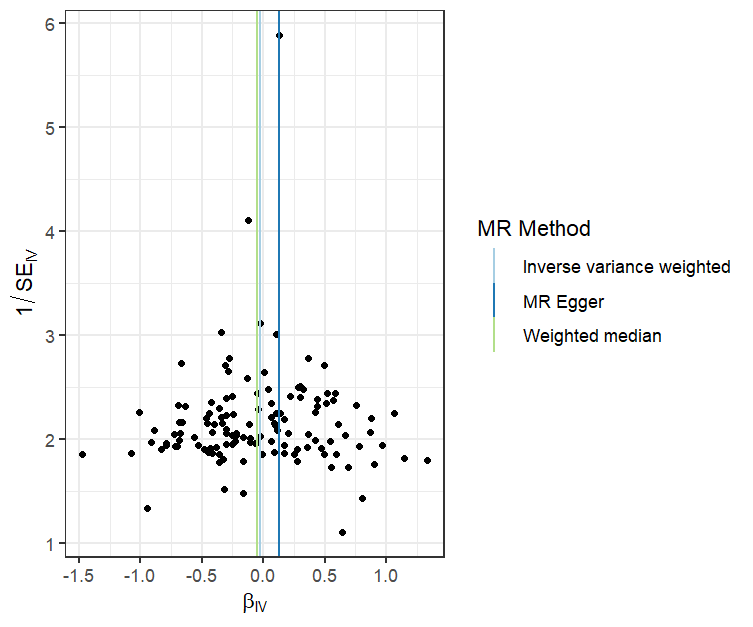
**

**(E)**

**
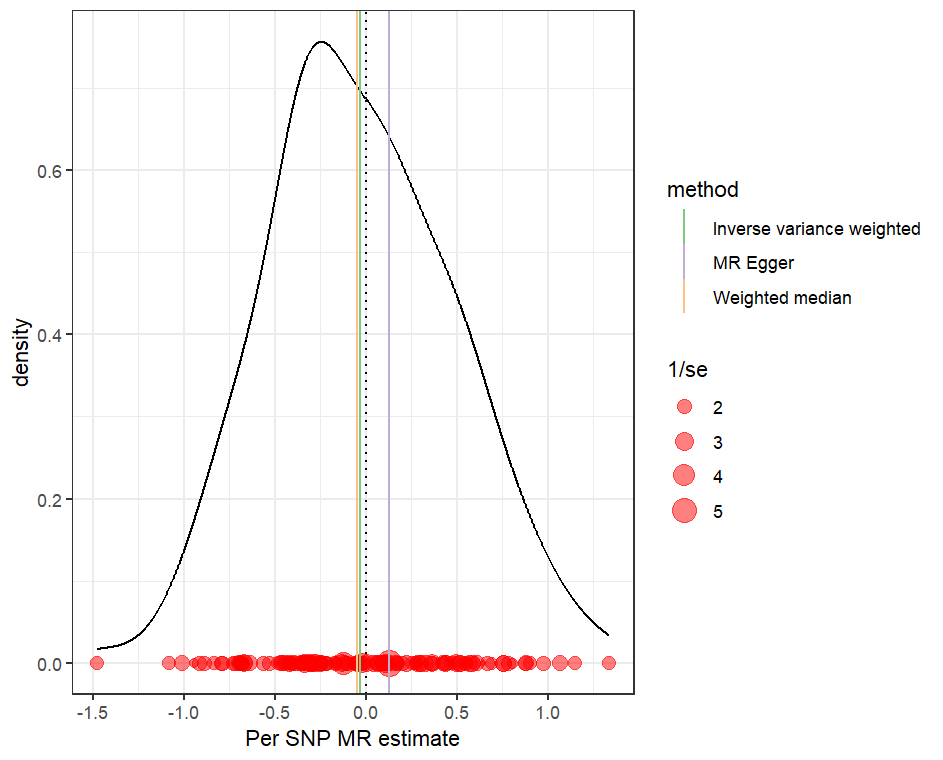
**

**Supplementary Figure S3 Forest plot (A), sensitivity analysis (B), scatter plot (C) , funnel plot (D) and density (E) of the causal effect of Daytime sleepiness on CRC risk。**

**(A) (B)**


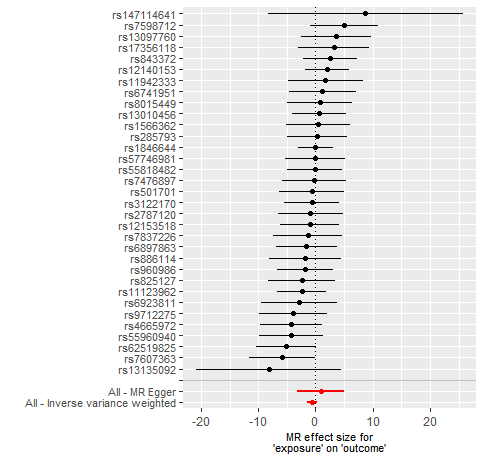

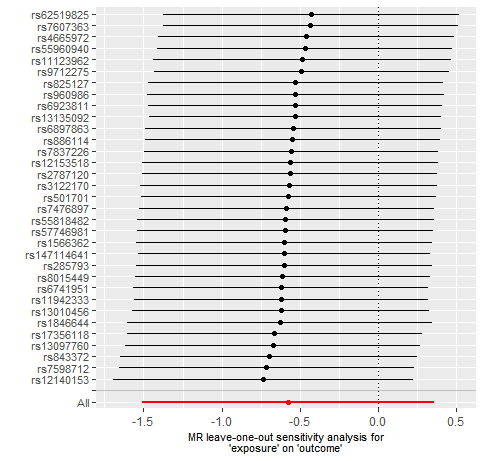


**(C) (D)**


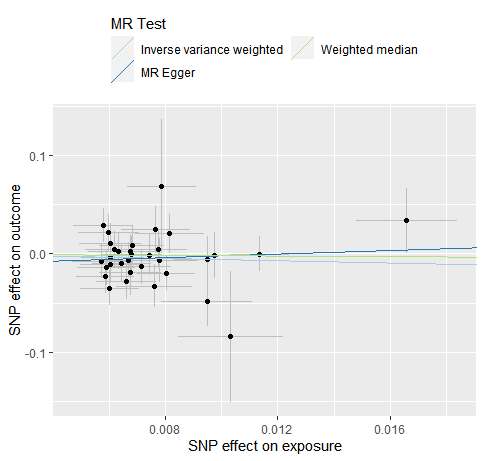

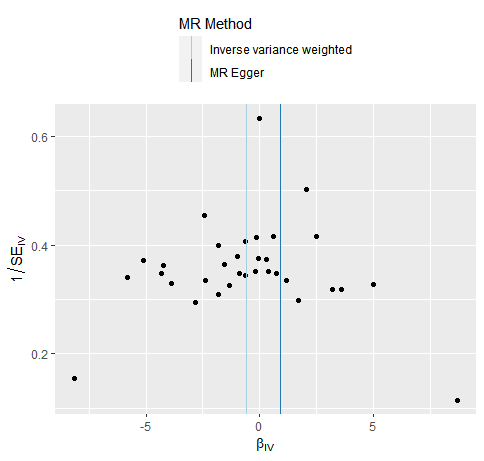


**(E)**

**
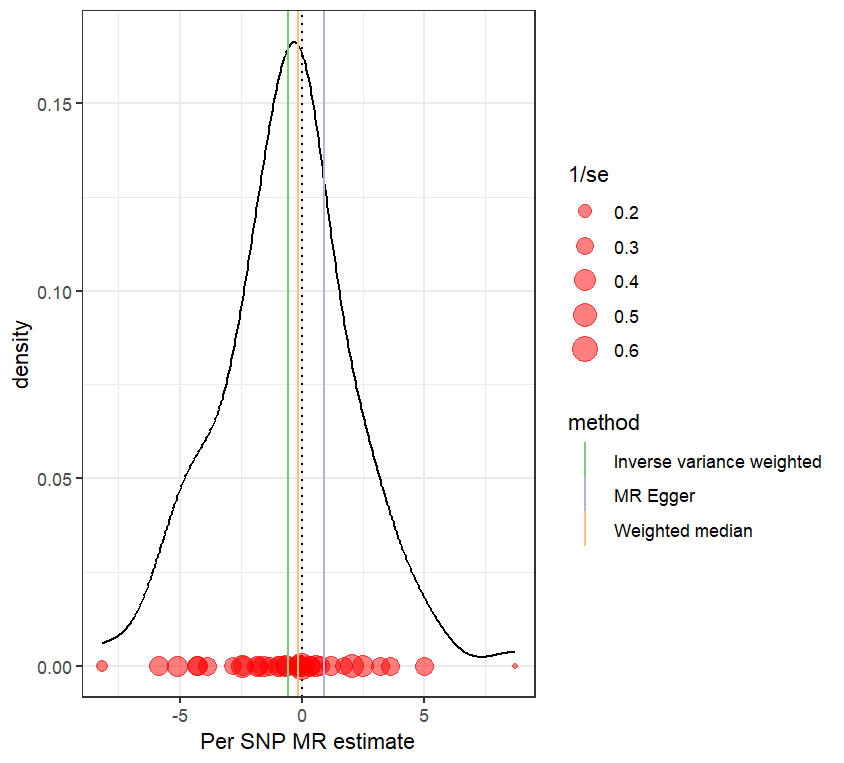
**

**Supplementary Figure S4 Forest plot (A), sensitivity analysis (B), scatter plot (C) , funnel plot (D) and density (E) of the causal effect of Daytime napping on CRC risk。**

1. **(B)**


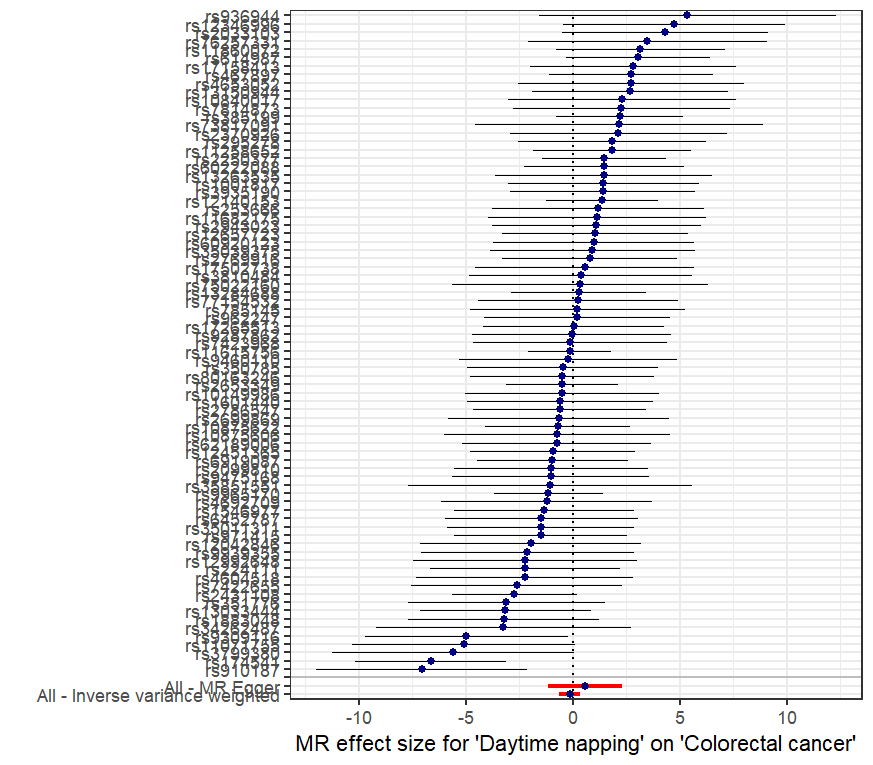

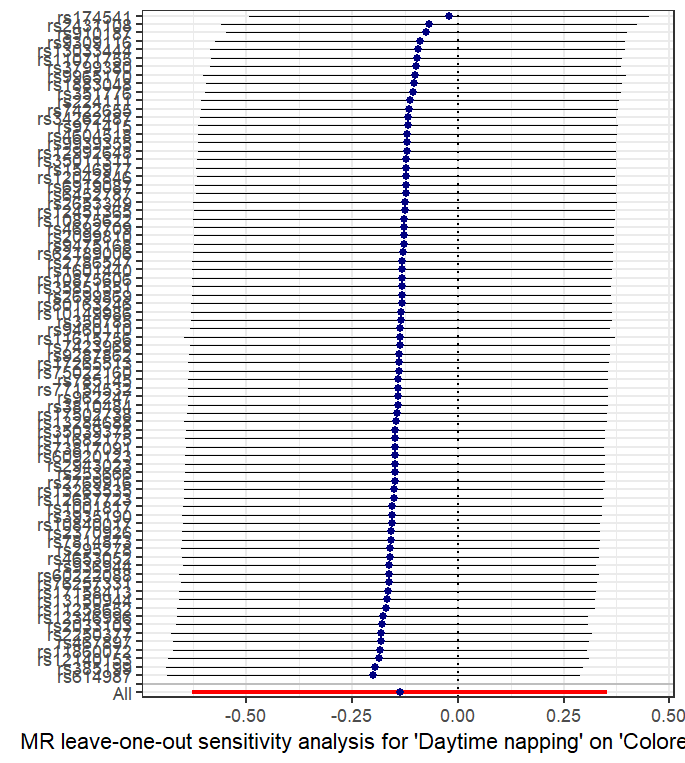


**(C)**


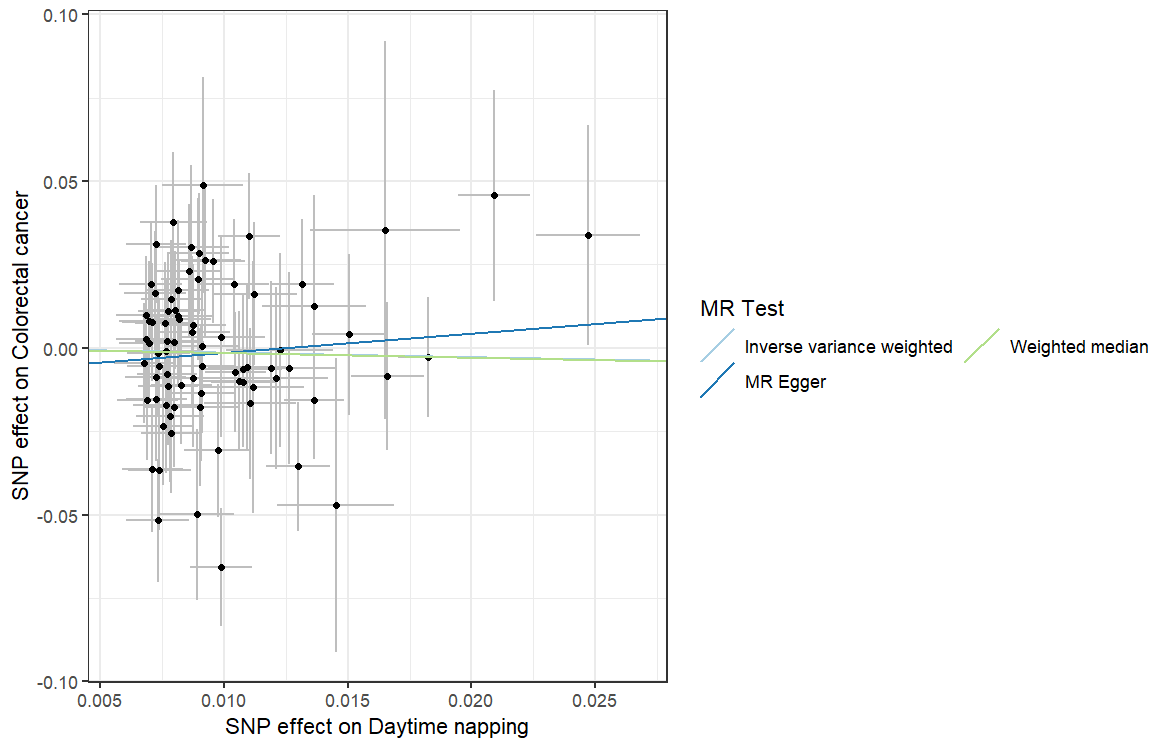


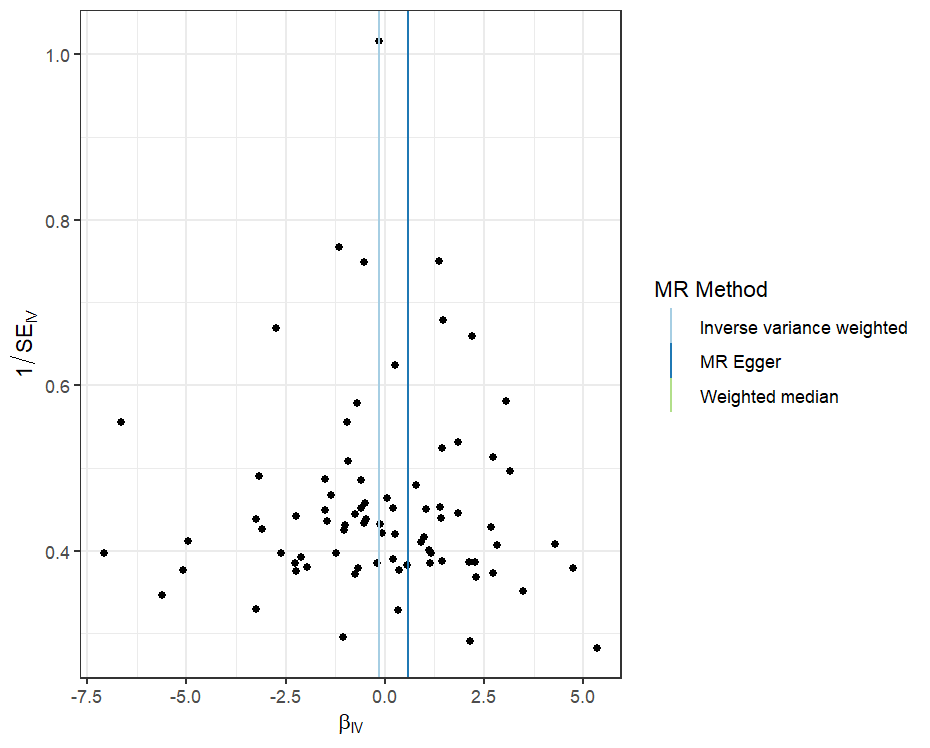


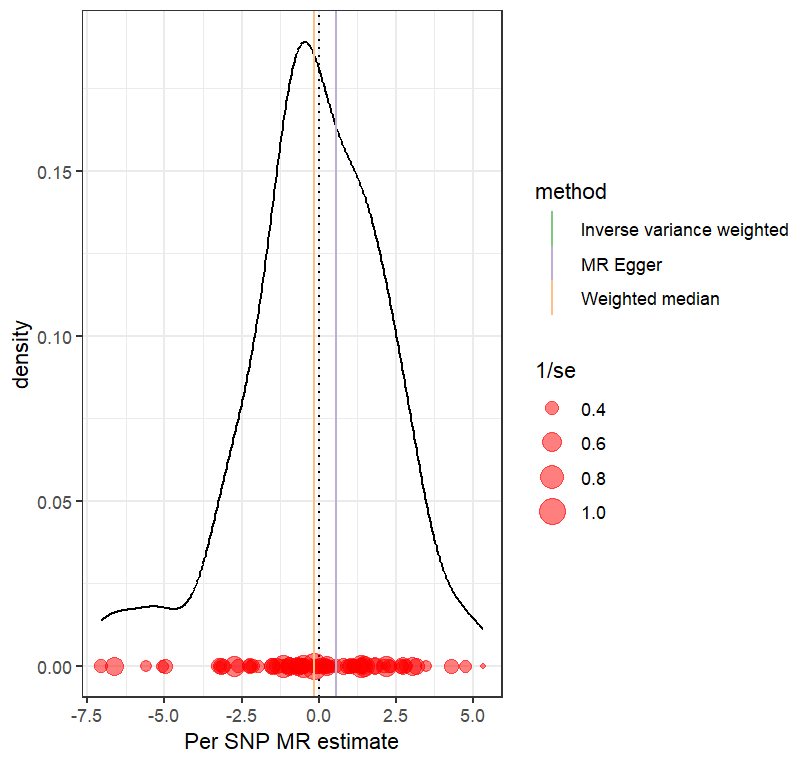


**Supplementary Figure S5 Forest plot (A), sensitivity analysis (B), scatter plot (C) , funnel plot (D) and density (E) of the causal effect of Chronotype on CRC risk after removing SNPs with potential pleiotropy。**


**
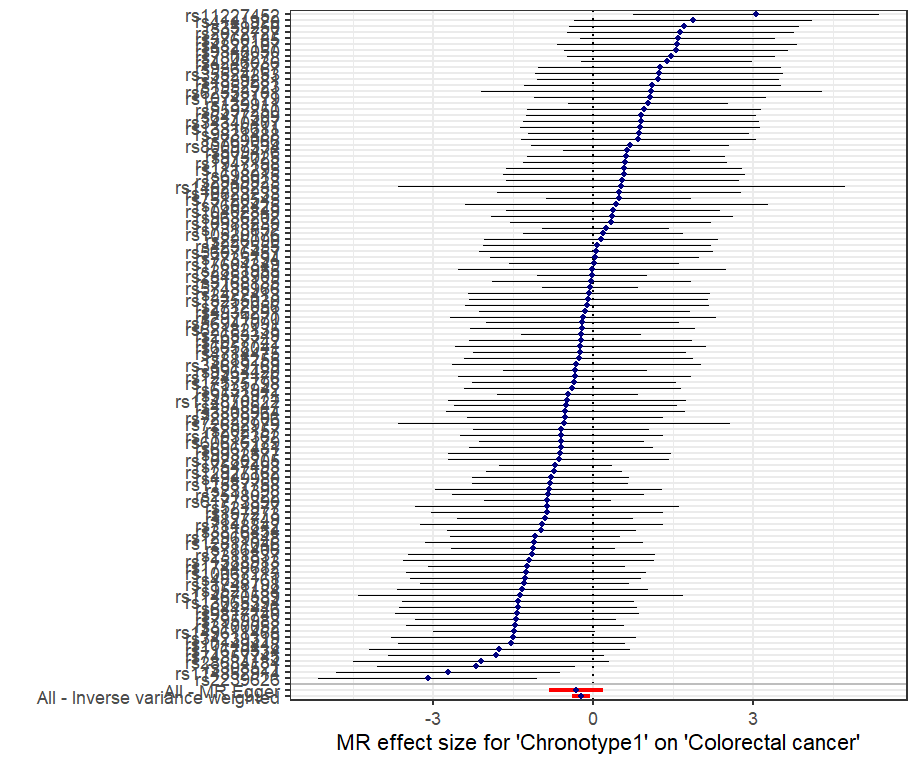
**


**
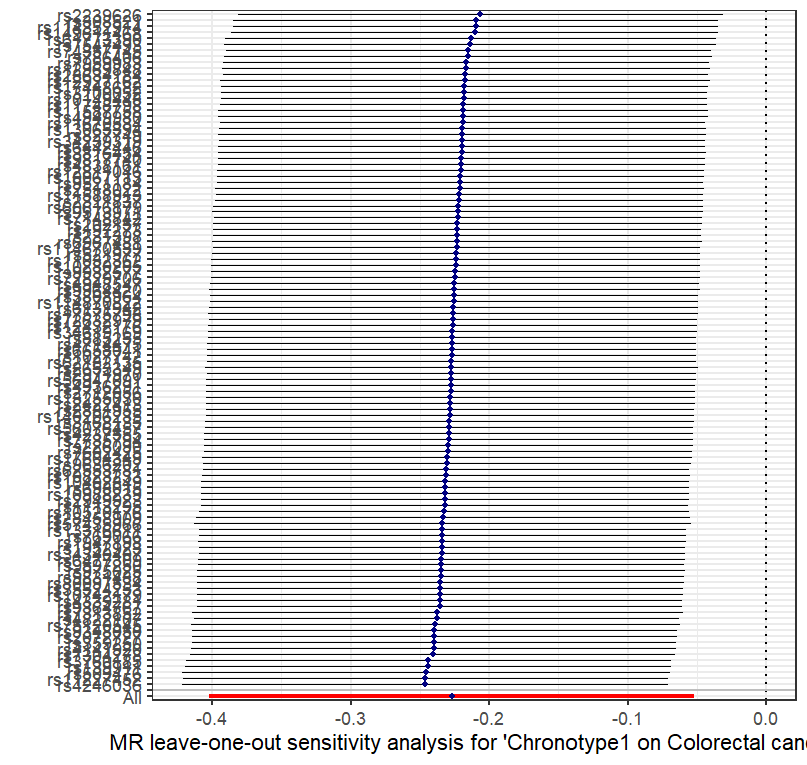
**

**(C)**

**
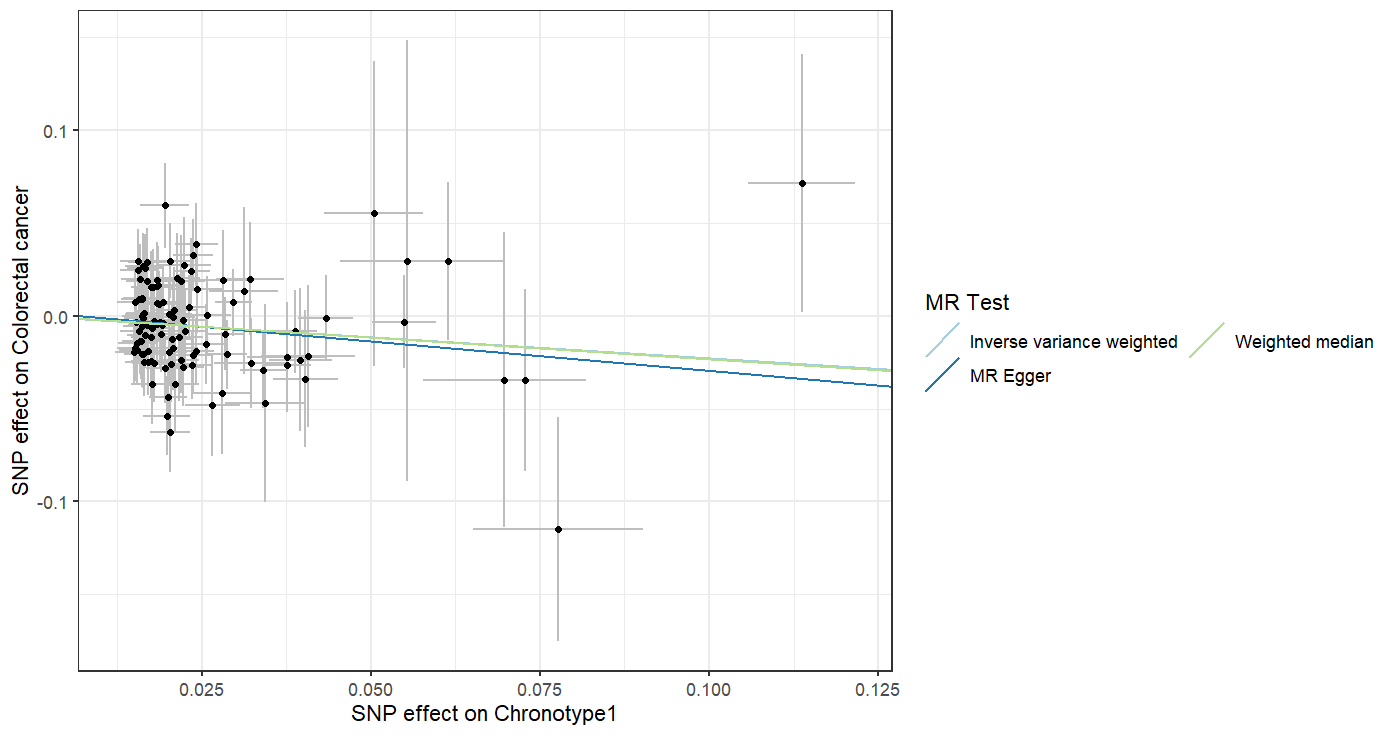
**

**(D)**

**
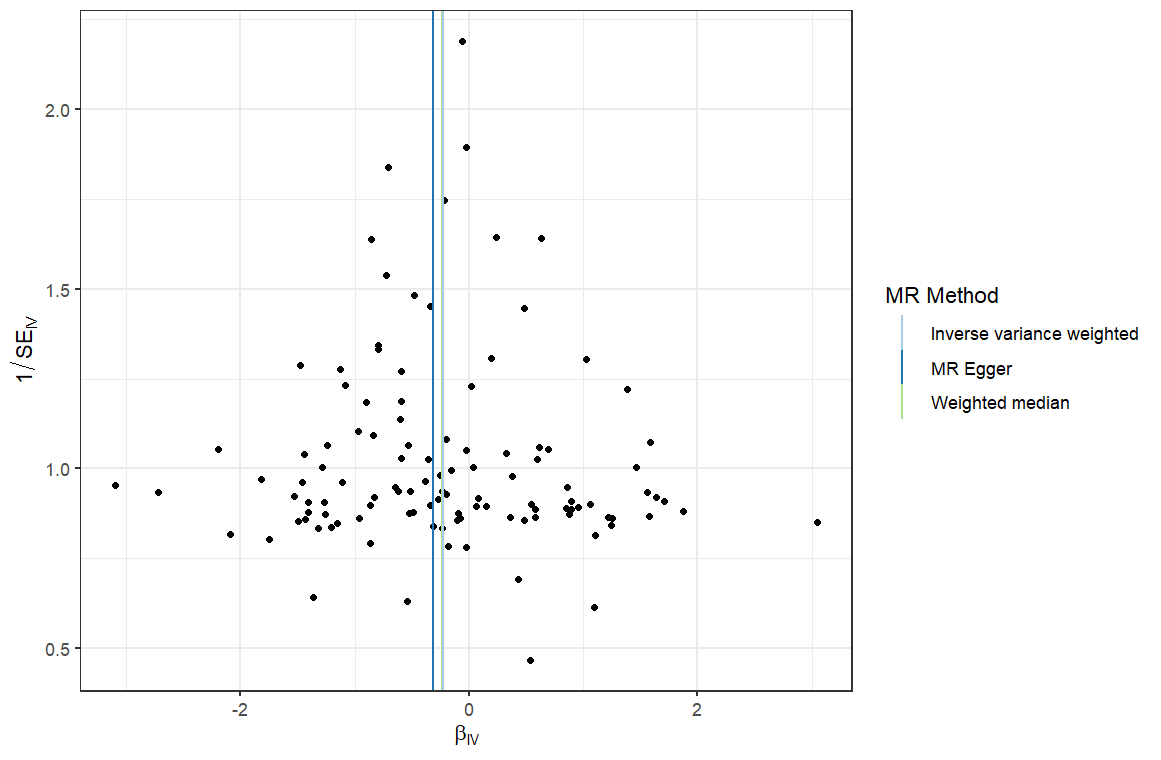
**

**(E)**

**
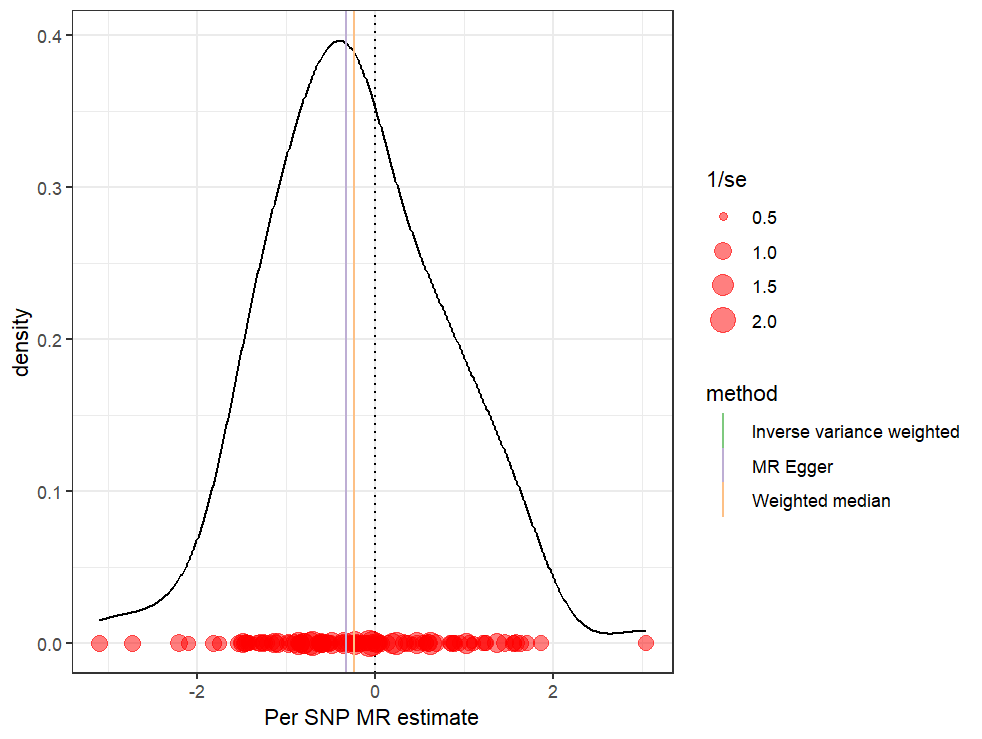
**

**Supplementary Figure S6 Forest plot (A), sensitivity analysis (B), scatter plot (C), funnel plot (D) and density (E) of the causal effect of Sleep duration on CRC risk after removing SNPs with potential pleiotropy。**

**(A) (B)**

**
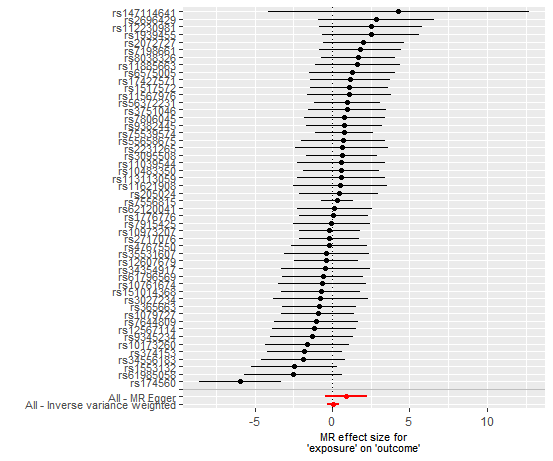

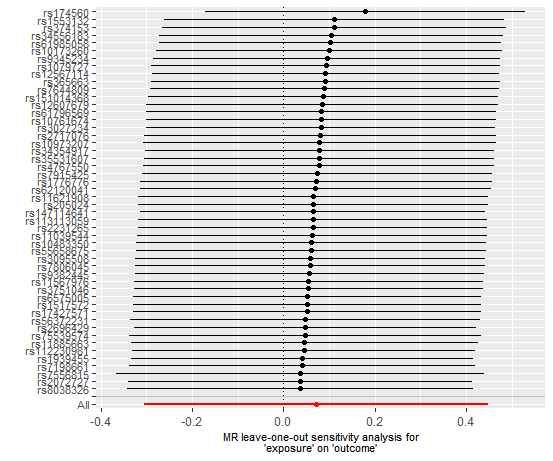
**

1. **(D)**

**
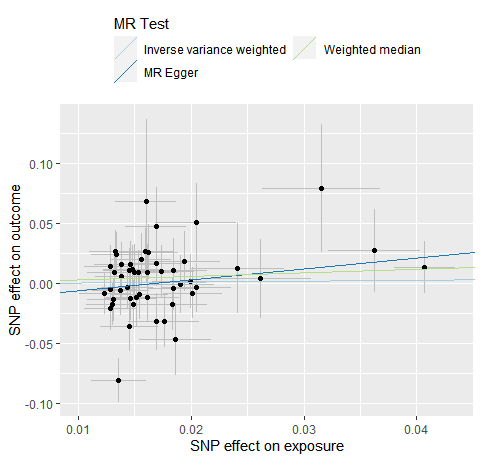

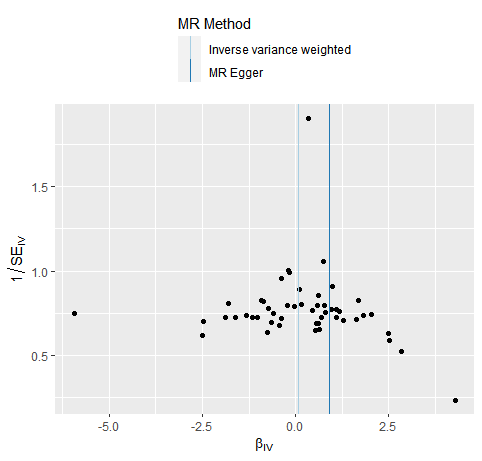
**

**(E)**

**
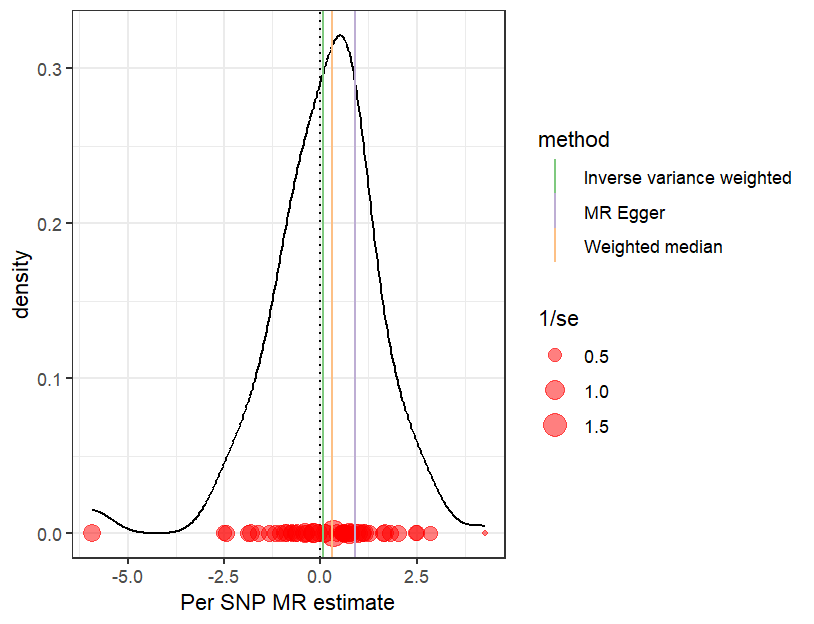
**

**Supplementary Figure S7 Forest plot (A), sensitivity analysis (B), scatter plot (C) , funnel plot (D) and density (E) of the causal effect of Insomnia on CRC risk after removing SNPs with potential pleiotropy。**

**(A)**

**
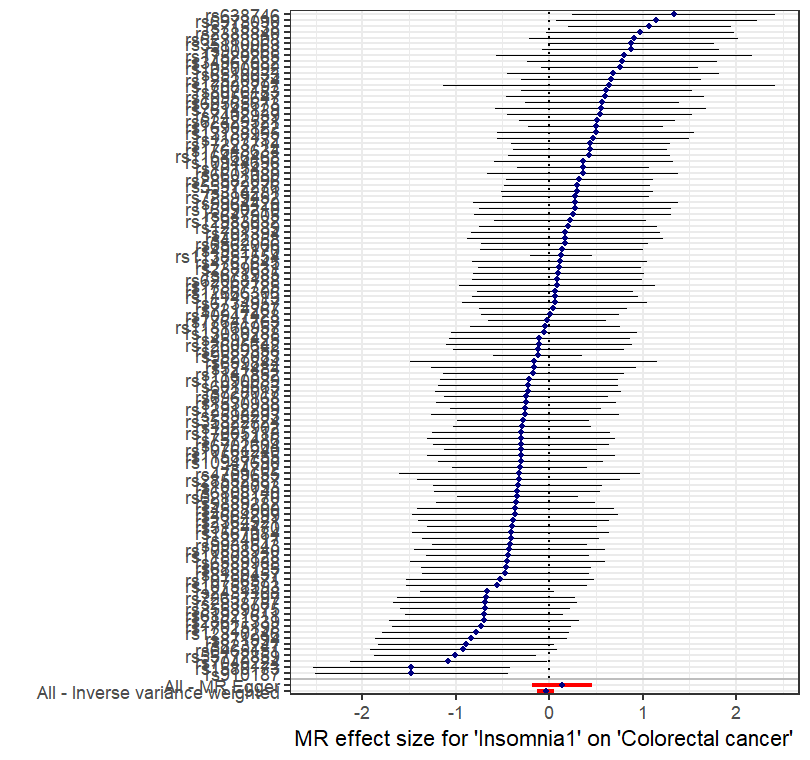
**

**(B)**

**
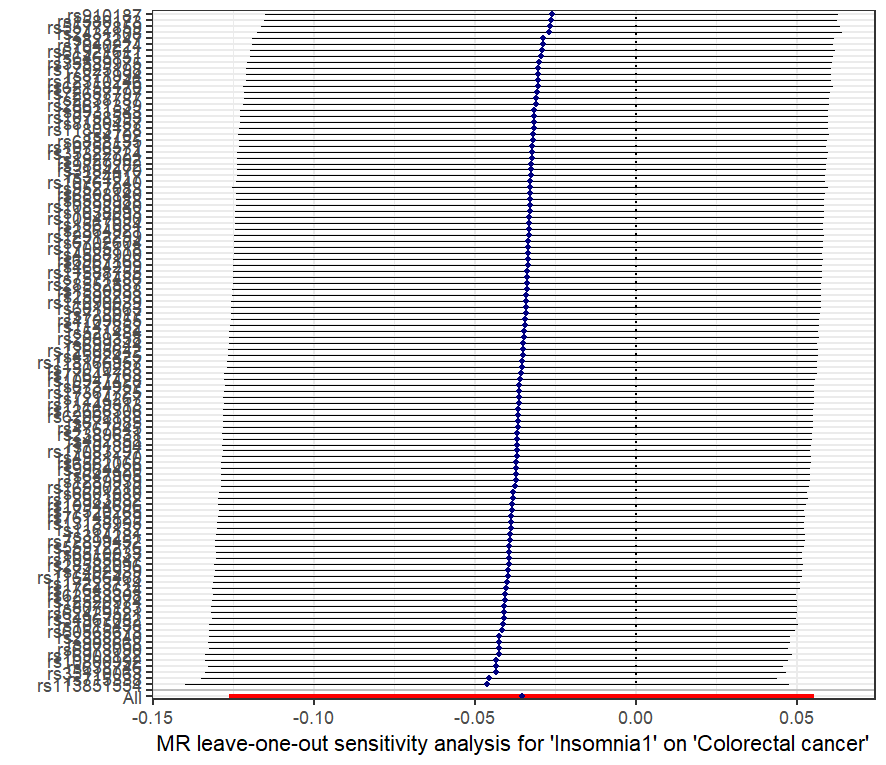
**

**(C)**

**
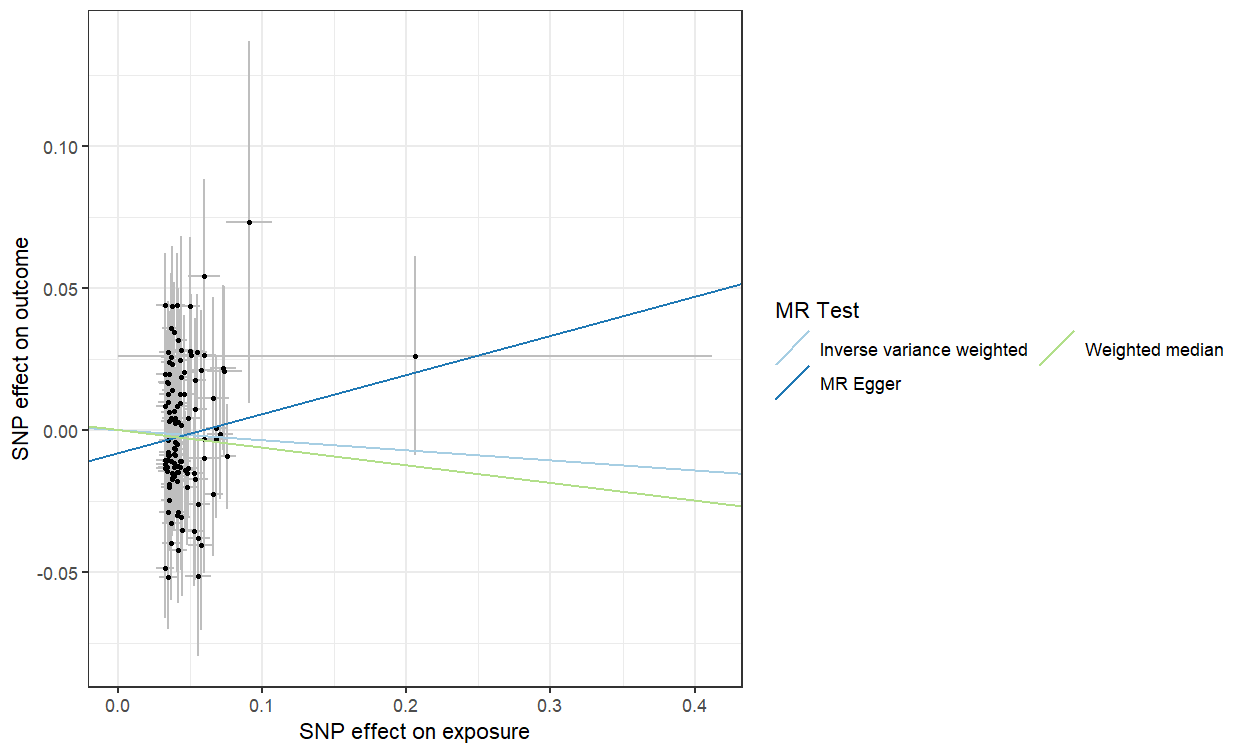
**

**(D)**

**
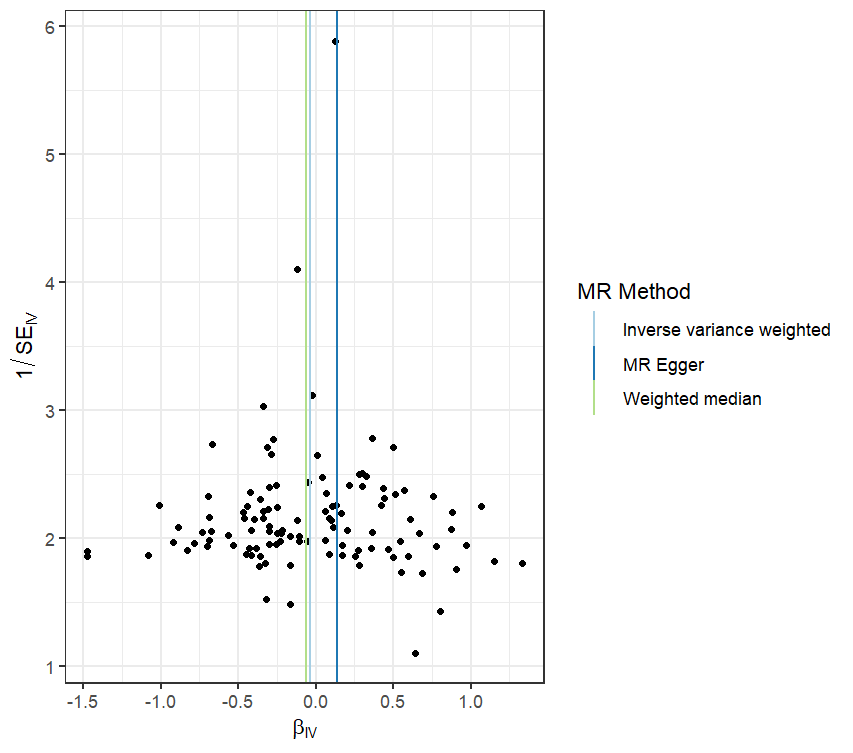
**

**(E)**

**
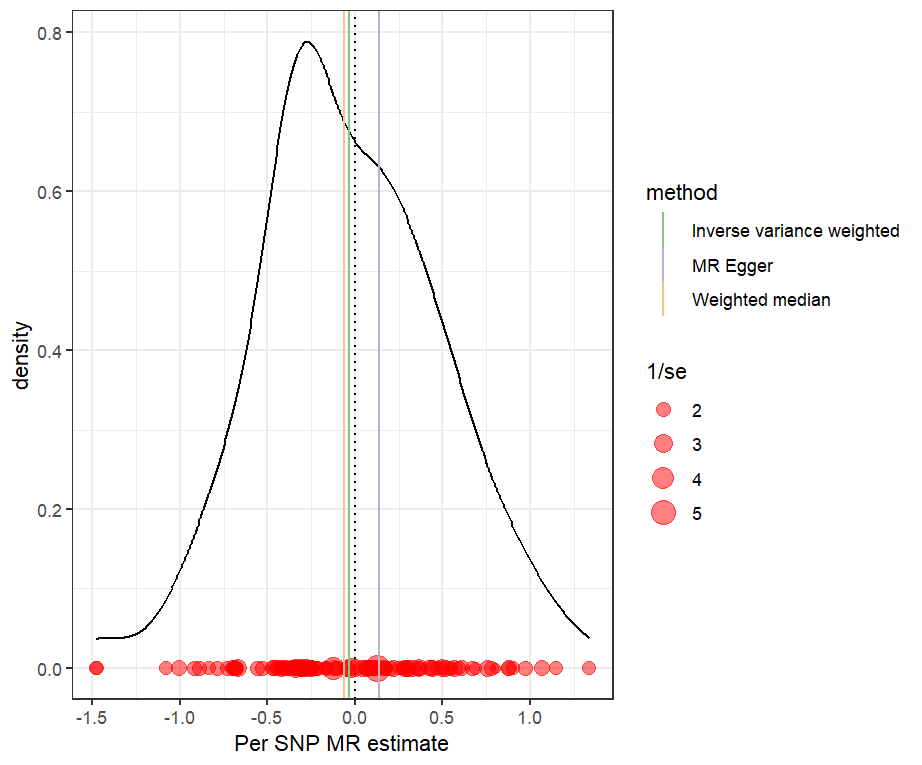
**

**Supplementary Figure S8 Forest plot (A), sensitivity analysis (B), scatter plot (C), funnel plot (D) and density (E) of the causal effect of Daytime sleepiness on CRC risk after removing SNPs with potential pleiotropy。**

**(A) (B)**

**
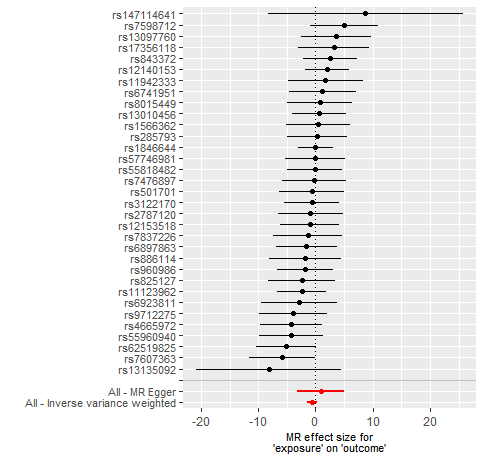

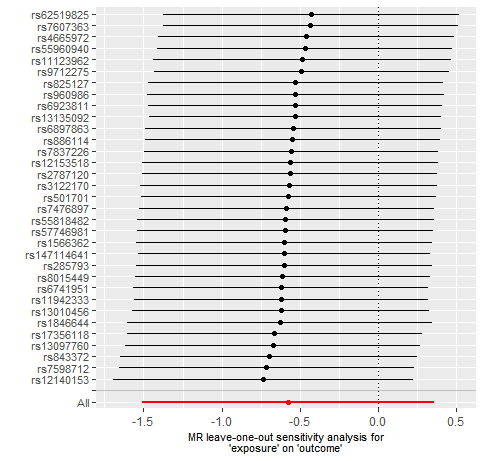
**

**(C) (D)**

**
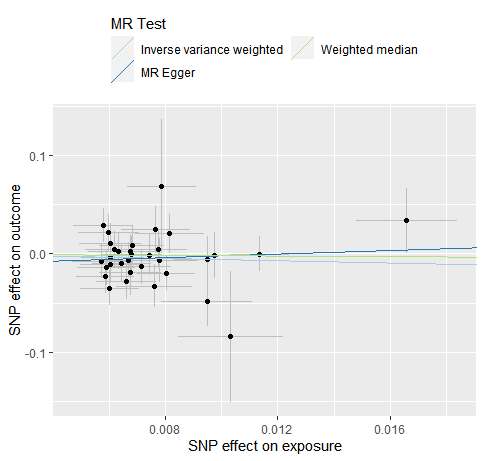

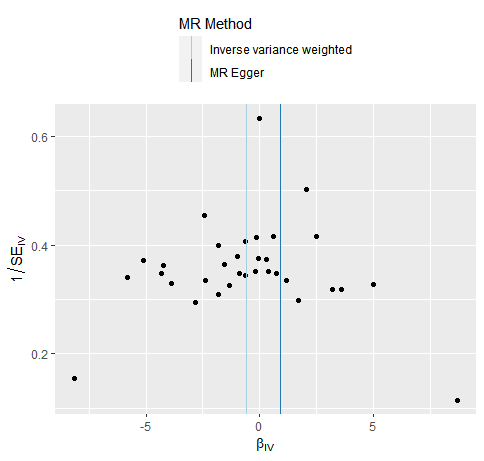
**

**(E)**

**
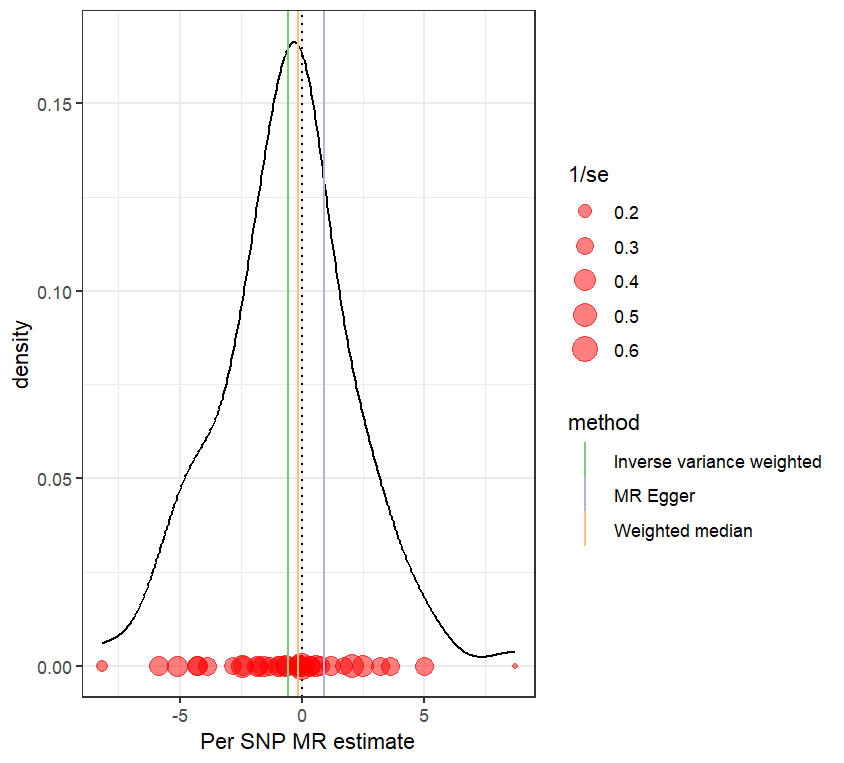
**

**Supplementary Figure S9 Forest plot (A), sensitivity analysis (B), scatter plot (C), funnel plot (D) and density (E) of the causal effect of Daytime napping on CRC risk after removing SNPs with potential pleiotropy。**

**(A)
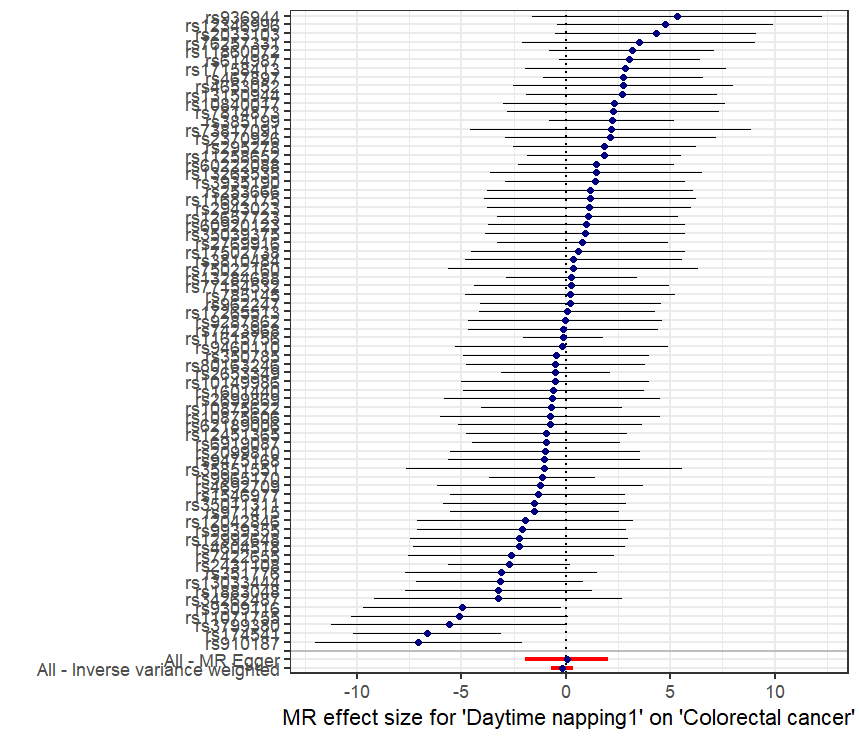
**

**(B)**

**
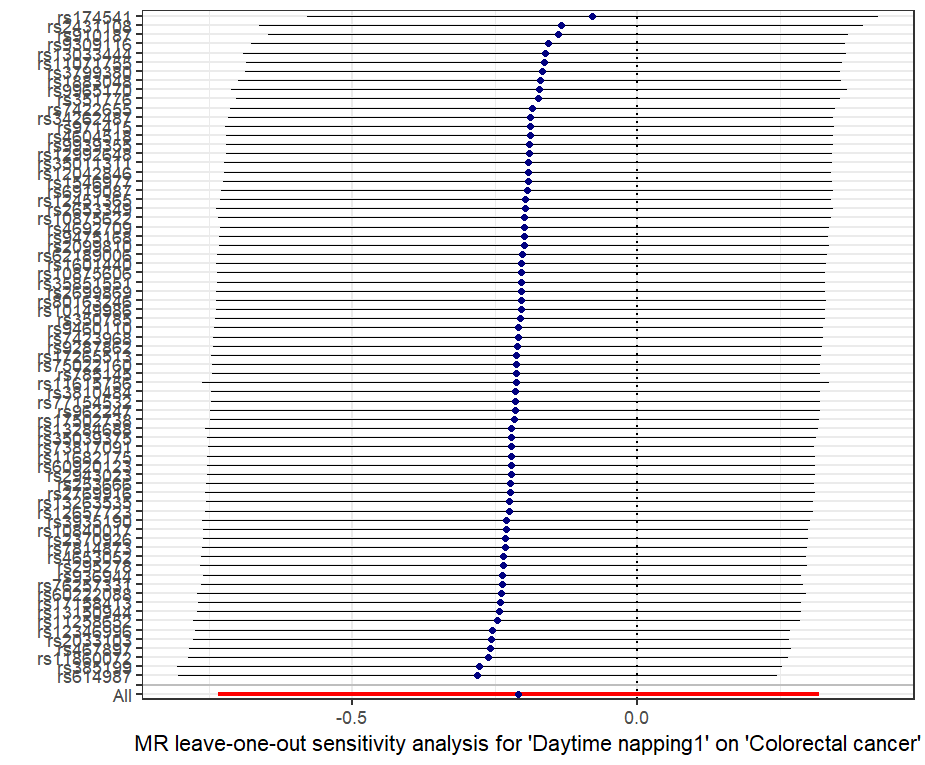
**

**(C) (D)**

**
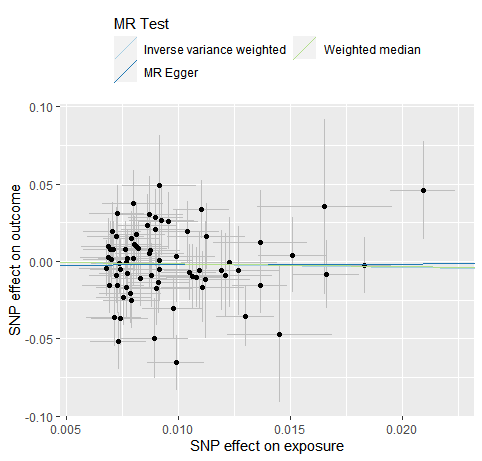

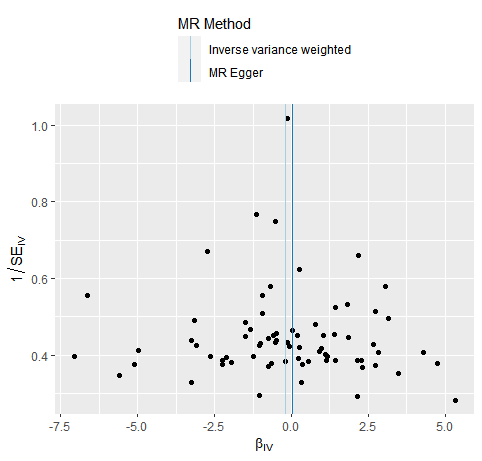
**

**(E)**

**
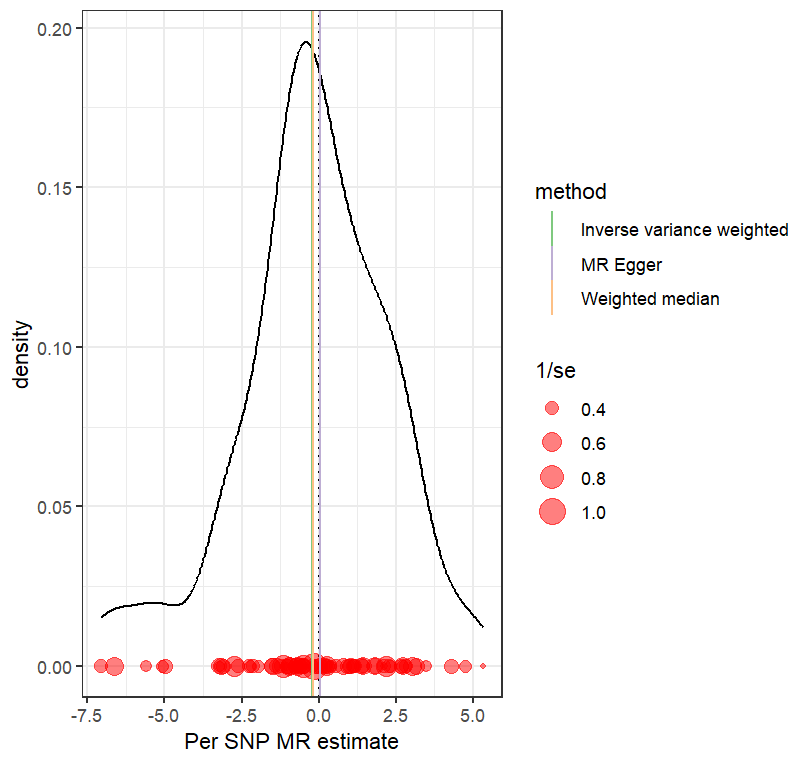
**
